# 15-LOX-catalytic bias towards ether-(alkenyl)-ETE-PEs oxidation bestows selectivity of PRO-ferroptotic cell death signaling

**DOI:** 10.1038/s41467-026-71869-z

**Published:** 2026-06-17

**Authors:** Yulia Y. Tyurina, Karolina Mikulska-Ruminska, Vladimir A. Tyurin, Brian A. Kleiboeker, Alexander A. Kapralov, Ayumi Hashimoto, Louis J. Sparvero, Haider H. Dar, Mert Akdogan, Kazuhiro Yamada, Jinming Zhao, Taha Kelestemur, Ecem Saritas, Sviatlana N. Samovich, Theodore R. Holman, Yuri L. Bunimovich, Yulia Nefedova, Dmitry I. Gabrilovich, Sally E. Wenzel, Ivet Bahar, Valerian E. Kagan, Hülya Bayır

**Affiliations:** 1 Department of Environmental and Occupational Health, School of Public Health, Pittsburgh, PA USA; 2 Center for Free Radical and Antioxidant Health, University of Pittsburgh, Pittsburgh, PA USA; 3 Institute of Physics, Faculty of Physics, Astronomy and Informatics, Nicolaus Copernicus University in Torun, Torun, Poland; 4 AstraZeneca, ICC, Early Oncology R&D, Gaithersburg, MD USA; 5 Department of Respiratory Medicine, Graduate School of Medicine, Osaka Metropolitan University, Osaka, Japan; 6 Department of Pediatrics, Division of Critical Care and Hospital Medicine, Children’s Redox Health Center, Vagelos College of Physicians and Surgeons, Columbia University Irving Medical Center, New York, NY USA; 7 Department of Chemistry and Biochemistry University of California Santa Cruz, Santa Cruz, CA USA; 8 Department of Dermatology, University of Pittsburgh School of Medicine, Pittsburgh, PA USA; 9 The Wistar Institute, Philadelphia, PA USA; 10 Laufer Center for Physical and Quantitative Biology, Stony Brook University, New York, NY USA; 11 Department of Biochemistry & Cell Biology, Renaissance School of Medicine, and Department of Chemistry, College of Arts & Sciences, Stony Brook University, Stony Brook, NY USA

**Keywords:** Phospholipids, Lipidomics

## Abstract

Ether (alkyl/alkenyl) phospholipids, particularly phosphatidylethanolamine (PE) and phosphatidylcholine (PC), are broadly represented in membranes, but their physiological functions are poorly characterized. The antioxidant role of plasmalogens realized via oxidation of *sn*-1 vinyl bond has been associated with anti-ferroptotic regulatory function. Alternatively, peroxidation of polyunsaturated fatty acid (PUFA) in *sn*-2-position of alkenyl-PEs can be pro-ferroptotic. Since 15-LOXs generate 15-HpETE-PEs as ferroptotic signals, we explored alkyl/alkenyl-ETE-PE as substrates of enzymatic peroxidation. Using redox lipidomics, biochemical, biophysical, genetic approaches, and molecular dynamics simulations, we established that both isoforms of 15-LOX (15-LOX-1 and 15-LOX-2) selectively oxidize alkyl/alkenyl-ETE-PE (but not alkyl/alkenyl-ETE-PC), forming 15-HpETE-PEs, triggering ferroptotic death, independently of the vinyl bond. We showed that LOX-catalyzed peroxidation rate of *sn*-1 vinyl bond is ~500-fold lower than *sn*-2-ETE-PE, thus excluding the antioxidant role of plasmalogens in ferroptosis. We showed 15-LOX-driven production of *sn*-1-alkenyl-*sn*-2-15-HpETE-PE acts as pathogenic factor in acute/chronic diseases: asthma, cancer, brain trauma, skin UVB-injury. Thus, 15-LOX-catalyzed bias towards oxidation of alkenyl-ETE-PE may represent a new therapeutic target.

## Abstract (web summary)

New functions and (patho)physiological role of broadly represented in membrane ether phospholipids as substrates of 15-lipoxygenases have been established in health and several acute/chronic diseases: asthma, cancer, brain trauma, skin UVB-injury.

## Introduction

Ether (alkyl/alkenyl) phospholipids, a subtype of glycerophospholipids with one chain being *sn-1*-alkenyl ether (as opposed to *sn-1-*acyl ester in diacyl-phospholipids), evolved early in anaerobic bacteria^1^ and persisted during eons of evolution, suggesting that they are indispensable for the functions of different cells and tissues^2^. Yet, their specific biochemical and physiological roles remain poorly understood, although their different phase behavior and packing density in biomembranes, as well as potential antioxidant propensities, have been documented^3^. Lately, studies of alkyl/alkenyl-phospholipids have focused on their significant roles not only in life but also in death, particularly in a type of regulated cell death called ferroptosis^4–6^, which uniquely utilizes robust phospholipid peroxidation as the mechanism of plasma membrane destruction.

Harmonized life of cell communities is maintained via timely elimination and clearance of irreparably damaged cells by programs of regulated death^7^. Among these, ferroptotic death is initiated in cells with dysregulated redox metabolism. The ferroptotic program utilizes robust membrane-perturbing lipid peroxidation (LPO) as an executioner of cells with uncontrolled pro-oxidant activity of iron and deficiency of thiols^8^. One of the central unresolved mechanistic questions in ferroptosis is whether LPO is induced: (i) enzymatically, (ii) by non-enzymatic chemical radical reactions, or (iii) by both of these processes.

Selectivity and specificity of LPO characteristic of ferroptosis favor its enzymatic nature, whereas LPO’s high sensitivity to a broad array of structurally diverse radical scavengers is interpreted as evidence of chemical mechanisms^9^. Of the myriad polyunsaturated phospholipid substrates available in biomembranes, only a few phosphatidylethanolamine (PE) species undergo massive peroxidation in ferroptosis^10^. Universally, polyunsaturated fatty acid (PUFA) containing PE, particularly *sn-2*-eicosatetraenoyl-(arachidonoyl, ETE)-PEs (ETE-PE) are oxidatively modified, yielding 15-HOO-ETE-PE (15-HpETE-PE) as the primary product^10^.

Two types of ETE-PE, differing only in the way the hydrophobic chains are attached to their glycerol backbone, are found in biomembranes. When the *sn*-1 hydrophobic chain is attached via an ether bond, an ether lipid is formed (alkyl/acyl-, alkenyl/acyl-PEs), while an ester bond attachment produces so called diacyl lipids (diacyl-PEs)^11,12^. Both forms commonly contain a non-oxidizable alcohol or acyl group in the *sn*-1 position, and a readily oxidizable PUFA, most frequently ETE acid, in the *sn*-2 position^12^. Intracellularly, alkenyl/acyl-PEs localize predominantly to the inner leaflet of the plasma membrane^13^, a site of potentially high “strategic” importance due to their participation in LPO as part of ferroptotic programs penultimately leading to plasma membrane injury. However, the role and mechanisms of alkenyl/acyl-*sn*-2-ETE-PE peroxidation in ferroptosis have not been fully understood, partly because of an accepted “antioxidant” reputation of alkenyl-PLs^14–16^. Recently, extensive use of genetic approaches established that the oxidizable alkenyl-*sn*-2-PUFA-phospholipids are pro-ferroptotic^4,6,17^. In contrast, another group of reports has shown peroxisomal and ether-phospholipid *deficiencies* to increase cellular vulnerability to ferroptosis^5,18^. Despite these controversies, there is a concordant opinion on the importance of alkenyl/acyl-PE peroxidation in the regulation and execution of ferroptosis^4–6^. It is possible that the use of stochastic models of random non-enzymatic LPO initiation, rather than specific enzymatic mechanisms, was responsible, at least in part, for these inconsistent results.

Structural work established that alkenyl/acyl-PEs are more densely packed in membranes than diacyl-PE^3^. These structural features of ether-PEs may reflect on their enzymatic transformations, particularly their conversions into oxygenated species. Given the established participation of 15-lipoxygenases (15-LOXs) in the initiation of ferroptosis^19,20^, we hypothesized that 15-LOX-dependent oxidation of acyl-PE and alkenyl-PEs is distinctly different and dictates relative contributions of these species to the execution of ferroptosis.

In the current work, we utilized biochemical, biophysical, genetic, cell, tissue, and animal models, along with extensive molecular dynamics simulations, and discovered that alkenyl/acyl-PE/diacyl-PE ratios define cellular susceptibility to ferroptosis mediated by preferential oxidation of the alkenyl species by 15-LOXs.

## Results

### Abundance and distribution of polyunsaturated diacyl- and alkenyl-phospholipids in cells and tissues

**Fig. 1:**
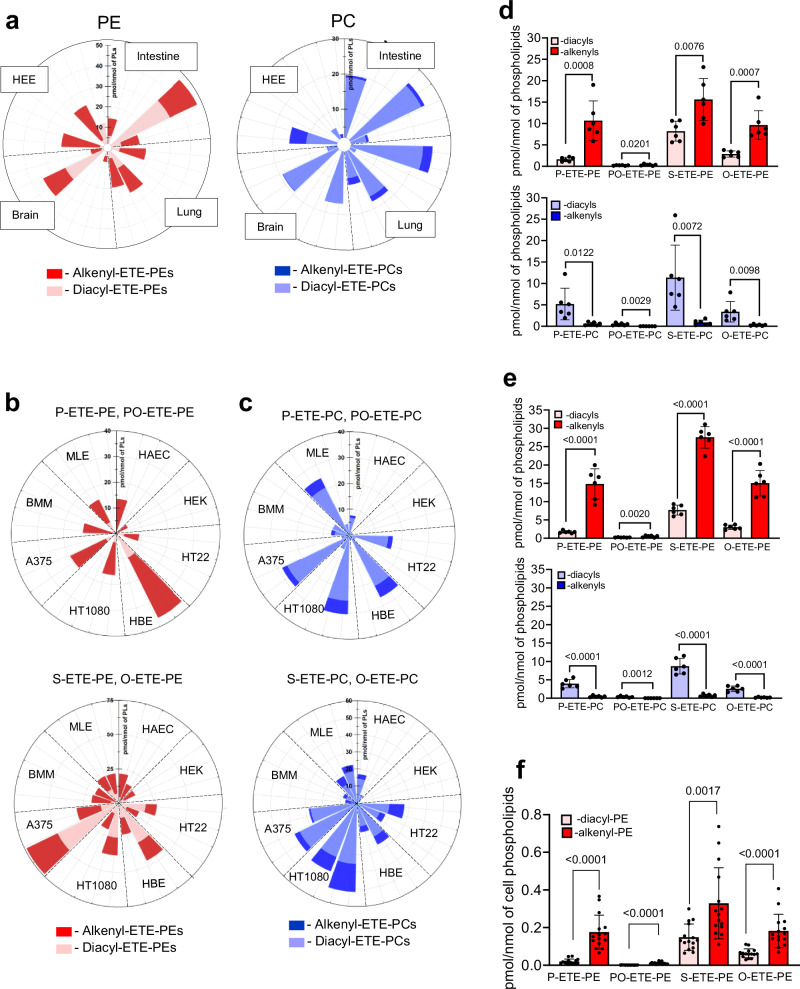
Distribution of eicosatetraenoyl (ETE) diacyl- and alkenyl-phospholipids in cells and tissues. **a** Content of diacyl- and alkenyl- ETE-PE (left) and ETE-PC (right) species in tissues. Human epidermal explants (HEE) (*n* = 3 biological samples from three different donors), mouse brain (*n* = 5 biological samples from five different mice), mouse intestine (*n* = 4 biological samples from four different mice), mouse lung (*n* = 5 biological samples from five different mice). Content of ETE-diacyl and alkenyl PE (**b**) and ETE-diacyl and alkenyl PC (**c**) containing palmitic (P) or palmitoleic (PO) (upper panel) and stearic (S) or oleic (O) (lower panel) fatty acids in *sn-1* positions in cells. *n* = 3 biological samples. HAEC – Human Airway Epithelial Cells (*n* = 3, three cultures from three different donors were used); HEK – Human Epidermal Keratinocytes (*n* = 3 biological samples); HT22 – Mouse Hippocampal Neuronal cells (*n* = 3 biological samples); HBE – Human Bronchial Epithelial Cells (*n* = 3 biological samples); HT1080 – Human epithelial cells derived from connective tissue from a patient with Fibrosarcoma (*n* = 3 biological samples); A375 – Human Melanoma cells (*n* = 4 biological samples); BMM – Bone Marrow Macrophages (*n* = 3 biological samples isolated from three different mice); MLE – Mouse Lung Epithelial Cells (*n* = 4 biological samples). Content of ETE- diacyl and alkenyl PE (upper panels) and PC (lower panels) species in cytosol (**d**), mitochondria (**e**), and plasma membrane (**f**) of HAECs. Mean ± SD, *n* = 5 (five HAEC cultures, each from different donors). For plasma membrane, three HAEC cultures, each from a different donor, were used, *n* = 5-6 biological replicates for each HAEC culture. Unpaired two-sided Student’s *t*-test. Cytosol: Diacyls vs alkenyls: *P*-ETE-PE (*t* = 7.702, *p* = 0.0008, df = 10), PO-ETE-PE (*t* = 2.760, *p* = 0.0201, df = 10), S-ETE-PE (*t* = 15.070, *p* = 0.0076, df=10), O-ETE-PE (*t* = 8.358, *p* = 0.0007, df = 10); P-ETE-PC (*t* = 7.396, *p* = 0.0122, df = 10), PO-ETE-PC (*t* = 4.479, *p* = 0.0029, df = 10), S-ETE-PC (*t* = 9.043, *p* = 0.0072, df = 10), O-ETE-PC (*t* = 7.582, *p* = 0.0098, df = 10). Mitochondria: Diacyls vs alkenyls: P-ETE-PE (*t* = 4.742, *p* < 0.0001, df = 10), PO-ETE-PE (*t* = 1.582, *p* = 0.0020, df = 10), S-ETE-PE (*t* = 3.330, *p* < 0.0001, df=10), O-ETE-PE (*t* = 4.821, *p* < 0.0001, df=10); P-ETE-PC (*t* = 3.050, *p* < 0.0001, df = 10), PO-ETE-PC (*t* = 3.917, *p* = 0.0012, df = 10), S-ETE-PC (*t* = 3.365, *p* < 0.0001, df = 10), O-ETE-PC (*t* = 3.181, *p* < 0.0001, df = 10). Plasma membrane: Diacyls vs alkenyls: P-ETE-PE (*t* = 6.622, *p* < 0.0001, df = 28), PO-ETE-PE (*t* = 6.136, *p* < 0.0001, df = 28), S-ETE-PE (*t* = 3.461, *p* = 0.0017, df = 32), O-ETE-PE (*t* = 5.051, *p* < 0.0001, df = 28). Source data are provided as a Source data file.

To get initial insight into the relationship between pro-ferroptotic PUFA-PE and LPO, we assessed the levels of readily oxidizable ETE acid-containing alkenyl/acyl- and diacyl-PEs in several tissues (Fig. 1a and Supplementary Fig. 1a) and cell types (Fig. 1b and Supplementary Fig. 1b). Alkenyl-PEs were the major polyunsaturated PE species in most examined tissues and cells, compared with the corresponding diacyl PE species (Fig. 1a, b). In contrast, polyunsaturated phosphatidylcholines (PCs) were mainly represented by diacyl-species (Fig. 1a, c). The ratios of ETE-containing alkenyl- to diacyl-phospholipids differed dramatically across various types of tissues, cells, and organelles, ranging from ~0.02 to ~16.0 (Supplementary Figs. 1 and 2).

To explore the subcellular distribution of PUFA-PE levels, ETE-containing alkenyl/acyl- and diacyl-PEs were assessed in several cell compartments of human airway epithelial cells (HAEC) and human bronchoalveolar (HBE) cells. In HAECs, alkenyl-S-ETE-PE were predominant species in the cytosol (Fig. 1d and Supplementary Fig. 1c) and mitochondria (Fig. 1e and Supplementary Fig. 1d). Because HAECs were derived from human subjects and therefore available only in limited amounts, we employed a highly sensitive, specific and quantitative protocol using fluorescamine-labeling of PE species^21^ to assess ETE-PE levels in the plasma membrane of HAECs. We found that the contents of alkenyl-ETE-PE species were significantly higher compared to their respective diacyl-ETE-PEs (Fig. 1f and Supplementary Fig. 1e). Among all ETE-PEs, alkenyl-ETE-PE species – palmitoyl-(P)-ETE-PE, palmitoleoyl- (PO)-ETE-PE, stearoyl-(S)-ETE-PE, and oleoyl-(O)-ETE-PE – were predominant in plasma membrane of HAECs.

Furthermore, we isolated mitochondria, endoplasmic reticulum (ER) and plasma membrane fractions from the more readily available HBE cells by density gradient centrifugation and performed analysis of diacyl- and alkenyl-ETE-PE as well as diacyl- and alkenyl-ETE-PC species. The distribution patterns of diacyl-ETE-PE and alkenyl-ETE-PE species were similar across plasma membrane, ER, and mitochondria. Among these species, alkenyl-P-ETE-PE and diacyl-S-ETE-PE were the predominant ones (Supplementary Fig. 2a, d–f). No significant differences were observed in the levels of diacyl-PO-ETE- and alkenyl-PO-ETE-PE as well as diacyl-O-ETE- and alkenyl-O-ETE-PE species. Similarly, using the protocol with fluorescamine-labelling, we found that in HBE cells, the levels of alkenyl-ETE-PE species were higher compared to corresponding diacyl-ETE-PE (except for one species with stearic acid in the *sn*-1 position (Supplementary Fig. 2b). This is in line with prior reports showing that PE species are predominantly localized to the cytosolic leaflet of the plasma membrane^13,22,23^. Analysis of PC species in fractions isolated from HBE cells revealed that diacyl-ETE-PC species constituted the major ETE-PC species in plasma membrane, ER, and mitochondria (Supplementary Fig. 2c–f). Among PE species, highly unsaturated ETE-containing alkenyl-PE species were present in the plasma membrane. The results of these assessments are consistent with published data reflecting the distribution of alkenyl-phospholipids in different biomembranes^24,25^.

### 15-LOX catalyzes selective oxidation of diacyl-ETE- and alkenyl-ETE-phospholipids in biochemical models

**Fig. 2:**
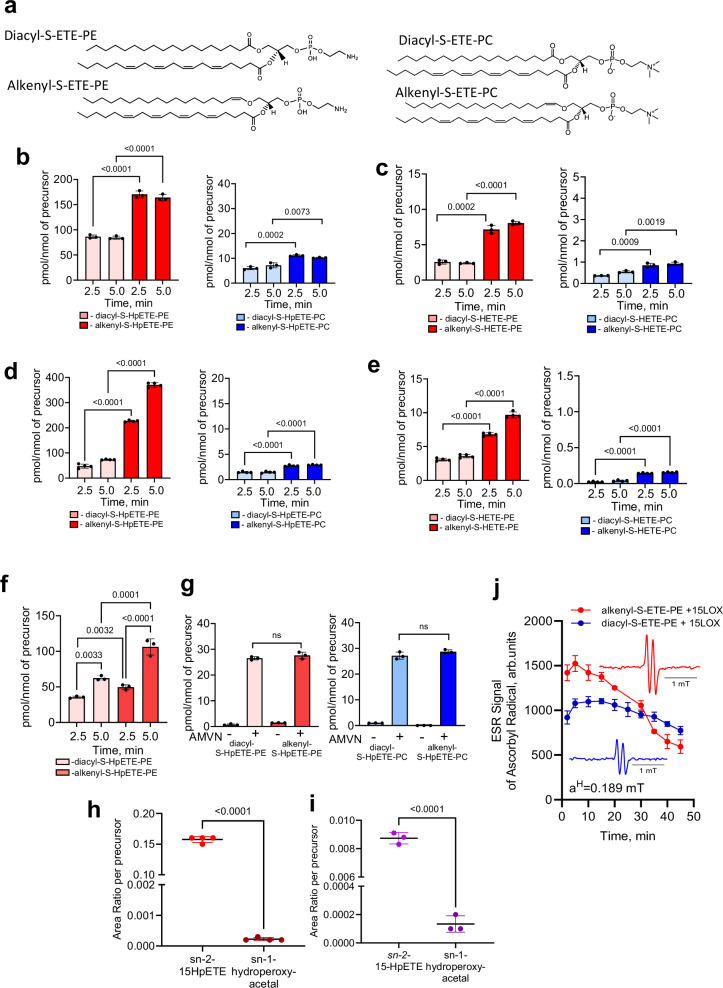
Oxidation of ETE-phospholipids catalyzed by 15-LOXs in biochemical models. **a** Structures of diacyl-S-ETE-PE, diacyl-S-ETE-PC, alkenyl-S-ETE-PE and alkenyl-S-ETE-PC. Content of 15-HpETE- and 15-HETE- PEs (left) and PCs (right) formed in 15-LOX-1 (**b**, **c**) and 15LOX-2 (**d**, **e**) driven reactions in dioleoyl-phosphatidylcholine (DOPC) liposomes. Mean ± SD, *n* = 3 independent experiments, unpaired two-sided Student’s *t*-test. 15-LOX-1/15-HpETE-phospholipids, diacyls vs alkenyls: S-HpETE-PE (*t* = 18.96, *p* < 0.0001, df = 4), S-HpETE-PC (*t *= 12.73, *p* = 0.0002, df = 4) at 2.5 min and S-HpETE-PE (*t* = 19.70, *p* < 0.0001, df = 4), S-HpETE-PC (*t* = 5.044, *p* = 0.0073, df = 4) at 5 min. 15-HETE-phospholipids, diacyls vs alkenyls: S-HETE-PE (*t* = 12.39, *p* = 0.0002, df = 4) and S-HETE-PC (*t* = 8.760, *p* = 0.0009, df = 4) at 2.5 min and S-HETE-PE (*t* = 36.01, *p* < 0.0001, df = 4) and S-HETE-PC (*t* = 7.309, *p* = 0.0019, df = 4) at 5 min. 15-LOX-2/15-HpETE-phospholipids, diacyls vs alkenyls: S-HpETE-PE (*t* = 40.60, *p* < 0.0001, df = 6) and S-HpETE-PC (*t* = 13.52, *p* < 0.0001, df = 6) at 2.5 min and S-HpETE-PE (*t* = 65.09, *p* < 0.0001, df = 6) and S-HpETE-PC (*t* = 16.82, *p* < 0.0001, df = 6) at 5 min. 15-HETE-phospholipids, diacyls vs alkenyls: S-HETE-PE (*t* = 23.03, *p* < 0.0001, df = 6) and S-HETE-PC (*t* = 33.94, *p* < 0.0001, df = 6) at 2.5 min and S-HETE-PE (*t* = 24.07, *p* < 0.0001, df = 6) and S-HETE-PC (*t* = 21.02, *p* < 0.0001, df = 6) at 5 min. **f** Content of S-HpETE-PEs formed in 15-LOX-2 driven reaction in liposomes prepared from HT22 lipids. Mean ± SD, *n* = 3 independent experiments, one-way ANOVA (*F* = 73.01, *p* < 0.0001, df = 11). **g** Content of S-HpETE-PEs (left) and S-HpETE-PCs (right) formed in the presence of 2,2’-azobis (2,4-dimethylvaleronitrile) (AMVN) in DOPC liposomes. Mean ± SD, *n* = 3 independent experiments, unpaired two-sided Student’s *t*-test. Level of alkenyl-S-15-HpETE-PE and hydroperoxyacetal-S-ETE-PE generated by 15-LOX-2 (**h**) and AMVN (**i**) in model systems. Unpaired two-sided Student’s *t*-test, 15-LOX-2: Mean ± SD, *n* = 4 independent samples, (*t* = 62.91, *p* < 0.0001, df = 6), AMVN: Mean ± SD, *n* = 3 independent samples (*t* = 36.49, *p* < 0.0001, df = 4). **j** Typical ESR spectra (inserts) and time-course of ascorbyl radicals generated by 15-LOX in the presence of alkenyl-S-ETE-PE (red) or diacyl-S-ETE-PE (blue) in DOPC liposomes. Ascorbyl radical EPR spectra shown are recorded at 2 min. Mean ± SD, *n* = 3 independent experiments. Source data are provided as a Source data file.

Given the important role of 15-LOX-catalyzed peroxidation of ETE-PE to 15-HpETE-PE in the initiation of ferroptosis, we examined the effectiveness of this reaction for: (i) alkenyl-S-ETE-PE vs alkenyl-S-ETE-PC and (ii) alkenyl-S-ETE-PE vs diacyl-S-ETE-PE species in biochemical model systems (Fig. 2a). We observed high selectivity (high peroxidation rate) for alkenyl-S-ETE-PE with both 15-LOX isoforms, 15-LOX-1 (Fig. 2b, c) and 15-LOX-2 (Fig. 2d, e). Assuming that this selectivity may be related to enzymatic mechanisms of phospholipid peroxidation, we directly compared 15-LOX-1 (Fig. 2b, c) and 15-LOX-2 (Fig. 2d, e) catalyzed peroxidation of alkenyl-S-ETE-PE vs diacyl-S-ETE-PE and diacyl-S-ETE-PC vs alkenyl-S-ETE-PC. Hydroperoxy- and hydroxy- derivatives of alkenyl-S-ETE-PE and diacyl-S-ETE-PE formed in 15-LOX-1 (Supplementary Fig. 3) and 15-LOX-2-driven reactions were identified by MS^2^ analysis (Supplementary Figs. 4a–d). The rate of alkenyl-S-ETE PE oxidation was ~5 times higher compared with that of diacyl-S-ETE-PE. Oxidation of either diacyl-S-ETE-PC or alkenyl-S-ETE-PC was very slow (Fig. 2b–e). We further examined whether this higher peroxidation effectiveness towards alkenyl-S-ETE-PE is observed in mixtures of phospholipids. To this end, we prepared liposomes using natural lipids obtained from HT22 cells. Similar to the results above, 15-LOX-2 induced oxidation of alkenyl-S-ETE-PE almost two times more effectively than diacyl-S-ETE-PE in these liposomes (Fig. 2f). This preferential peroxidation of alkenyl-S-ETE-PE was not observed when peroxidation of phospholipids was initiated non-enzymatically by a lipophilic initiator of peroxyl radicals, 2,2′-azobis (2,4-dimethylvaleronitrile) (AMVN) (Fig. 2g).

Considering that 15-LOX derived peroxyl radicals can attack the vinyl bond of alkenyl-PE^26^ and cause its oxidation, resulting in the generation of hydroperoxyacetal-containing species^27^, we compared the rates of oxidative modification of the vinyl bond in the *sn*-1 position with peroxidation of *sn*-2 ETE in the same alkenyl-S-ETE-PE. We employed enzymatic (15-LOX catalyzed) and non-enzymatic (AMVN-dependent) inducers of peroxidation. With both triggers, peroxidation of ETE in the *sn*-2 position of PE occurred at a ~500-fold higher rate than oxidation of the vinyl bond (Fig. 2h, i) as assessed by the accumulation of S-15-HpETE-PE (*sn*-2-hydroperoxy, OOH) and 1-OOH-S-ETE-PE (*sn*-1-hydroperoxyacetal) (Supplementary Fig. 4e). Furthermore, the rate of *sn*-2-ETE-PE peroxidation induced by AMVN was not affected by the presence of the *sn*-1 vinyl group and was approximately the same in alkenyl-S-ETE-PE and diacyl-S-ETE-PE (Fig. 2g). Together, these results suggest that sacrificial antioxidant capacities of the *sn*-1 vinyl ether are unlikely to override *sn*-2 PUFA peroxidation in response to enzymatic or non-enzymatic oxidation of plasmalogens.

To further test 15-LOX effectiveness towards alkenyl-S-ETE-PE oxidation, we employed an independent technique, electron spin resonance (ESR) spectroscopy. We examined the formation of lipid peroxyl radicals by 15-LOX either from alkenyl-S-ETE-PE or diacyl-S-ETE-PE by assessing the formation of readily ESR-detectable one-electron oxidation intermediates of ascorbate—semidehydroascorbyl radicals^28^. 15-LOX oxidation of either alkenyl-S-ETE-PE or diacyl-S-ETE-PE generates carbon-centered radicals, which in aerobic conditions are converted into peroxyl radicals^29^; which can readily oxidize ascorbate to its radicals^30^. We found that incubation of 15-LOX in the presence of ETE-PEs and ascorbate triggered the formation of semidehydroascorbyl radical ESR signals with the hyperfine splitting of the doublet signal of a^H^ = 0.189 mT (Fig. 2j). The magnitude of radical signal was significantly higher in the presence of alkenyl-S-ETE-PE than diacyl-S-ETE-PE. Time-course monitoring of the reaction showed a much faster disappearance of the characteristic ESR signals in the presence of alkenyl-S-ETE-PE than diacyl-S-ETE-PE, indicating a substantially lower rate of semidehydroascorbyl radical generation with diacyl-S-ETE-PE (Fig. 2j). These results further suggest that alkenyl-S-ETE PE—rather than diacyl-S-ETE-PE—is a preferred substrate for 15-LOX.

### Molecular dynamics simulations of 15-LOX interactions with alkenyl-S-ETE-PE vs alkenyl-S-ETE-PC

**Fig. 3:**
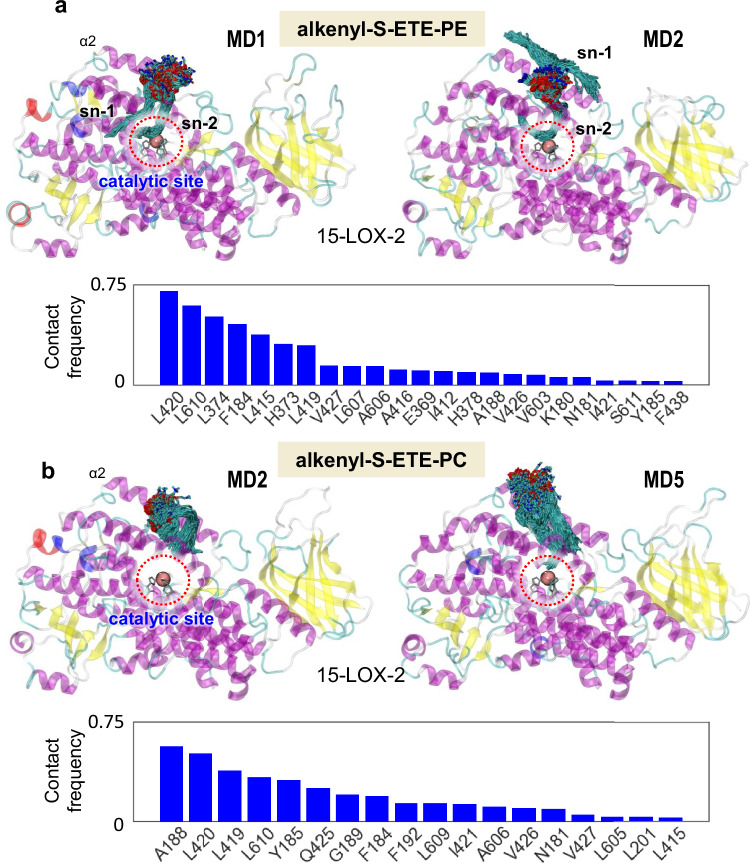
15-LOX interactions with alkenyl-ETE-PE vs alkenyl-ETE-PC observed in all-atom MD simulations. 15-LOX-2 structures are displayed with conformational variability of **a** alkenyl-S-ETE-PE or **b** alkenyl-S-ETE-PC exemplified on two MD runs each (MD1 (*upper left panel a*) and MD2 (upper *right panel a*) for alkenyl-S-ETE-PE and MD2 (*upper left panel b)* and MD5 (*upper right panel b*) for alkenyl-S-ETE-PC). Substrate conformations are displayed every 1 ns from 100 to 300 ns of MD simulations. The first period was omitted due to the significant changes in alkenyl-S-ETE-PC systems. The most frequent contacts observed in the last 100 ns of five independent MD runs (MD1-MD5), maintained by the region rich in double bonds (C5-C15 in sn-2 acyl chain) in alkenyl-S-ETE-phospholipids, are shown on the bar plots (see *lower panels in (a) and (b)*). Residue contact frequency is normalized to the total number of analyzed frames.

We further performed structural studies aimed at elucidating the molecular mechanisms responsible for the selectivity and specificity of alkenyl-ETE-PE peroxidation. We conducted five independent all-atom molecular dynamics (MD) simulations with either alkenyl-S-ETE-PE or alkenyl-S-ETE-PC bound to 15-LOX-2. Their initial positions were determined using molecular docking simulations (Supplementary Fig. 5, the insets). The highest affinity poses were bound to the catalytic site through their *sn*-2 chains with proximate energy −7.3 kcal/mol. The behavior of alkenyl-S-ETE-PLs, however, differed drastically in MD simulations when 15-LOX-2 was allowed to undergo conformational changes. The *sn*-2 chain of alkenyl-S-ETE-PEs constantly formed stable interactions with the hydrophobic core composed of L420, L610, L415, L374, F184, V419, L373, and the catalytic residue H373 in all MD runs (bar plot in Fig. 3a). This kept the oxidizable *sn*-2 chain in a conformation conducive of peroxidation (Supplementary Movies 1-2). The non-oxidizable *sn*-1 chain remained near the α2 helix, but occasionally, it could also enter the catalytic site (see Fig. 3a, MD1, and full trajectory in Supplementary Movie 2). However, this did not affect the *sn*-2 chain interactions with the catalytic site, as we showed previously for diacyl-S-ETE-PEs^31^. In contrast, alkenyl-PCs almost immediately left the catalytic site, subsequently remaining at the entrance of the enzyme (Fig. 3b and Supplementary Movies 3–4), interacting with A188, L420, L419, L610, and Y185 (bar plot in Fig. 3b). The final conformations of alkenyl-S-ETE-PLs after 300 ns of independent MD runs are presented in Supplementary Fig. 5. Overall, this computational modeling confirmed our experimental observations that alkenyl-S-ETE-PCs, in contrast to alkenyl-S-ETE-PEs, do not undergo peroxidation by 15-LOX-2. Even though they were placed in the catalytic center, alkenyl-S-ETE-PCs did not maintain sustained contact with the catalytic site. Moreover, the *sn*-1 chain in alkenyl-S-ETE-PEs, unlike diacyl-S-ETE-PEs (which have been thoroughly studied and described in our previous work^32^), did not compete with the *sn*-2 chain for occupancy of the catalytic site, thus explaining why the oxidation rate for alkenyl-S-ETE-PEs is higher than for diacyl-S-ETE-PEs.

### MD simulations demonstrate that PEBP1 binding has no significant effect on the interaction of alkenyl-ETE-PE with 15-LOX-2

Our previous work established that 15-LOX-catalyzed peroxidation of diacyl-S-ETE-PE was stimulated by PEBP1^31,32^. To examine the effect of PEBP1 on the oxidation of alkenyl-S-ETE-PEs, we further conducted eight MD simulations of alkenyl-S-ETE-PE bound to the 15-LOX-2/PEBP1 complex and compared them with our previous findings for diacyl-S-ETE-PE^31^. Surprisingly, simulations implied no significant role of PEBP1 in modulating alkenyl-S-ETE-PE binding at the catalytic site Supplementary Figs. 5 and 6 and Supplementary Movies 5–6), as the *sn*-1 acyl chain of alkenyl-S-ETE-PE (in *brown*), in contrast to that of diacyl-S-ETE-PE, did not interact with PEBP1 (*red dashed lines*), but instead formed stable interactions with 15-LOX-2 aligning along the α2 helix as observed in both PEBP1-bound and -unbound forms (Supplementary Fig. 6). Out of 8 MD runs in the presence of PEBP1, alkenyl-S-ETE-PE had no interactions with PEBP1 in 6 runs, while its *sn*-2 chain was stably bound to the catalytic site in all 8 runs.

The inability of alkenyl-S-ETE-PE to form stable interactions with the PEBP1 binding site likely stems from the absence of the polar ester linkage present at the *sn*-1 position in diacyl-S-ETE-PE. In the latter, the ester linkage engages L609 and L605, stabilizing the central region of the lipid backbone (see Supplementary Fig. 7a, *snapshot* **2,** *red oval*), thereby enabling the *sn*-1 chain to insert into a hydrophobic cavity within PEBP1 while the *sn*-2 chain binds to the catalytic site of 15-LOX-2. This insertion is facilitated by the interactions with polar residues, including F149 from 15-LOX-2 and F192 and H145 from PEBP1 (Supplementary Fig. 7a, *snapshot* **3,** *blue oval*). At this stage, the lipid headgroup begins to interact with H145 and S142 from PEBP1 (Supplementary Fig. 7a, *snapshot* **4,** *yellow oval*), further anchoring the molecule within the complex, providing a stable diacyl-S-ETE-PE bound pose compared to its unbound form (−7.2 kcal/mol vs −5.2 kcal/mol; Supplementary Fig. 7b, *top panel*). This mechanism additionally prevents the *sn*-1 chain of diacyl-S-ETE-PE from entering the catalytic site to destabilize the productive conformation of the *sn*-2 chain^31^.

Overall, the contribution of PEBP1 appears dispensable for alkenyl-S-ETE-PE, likely due to the conformational constraints imposed by the vinyl ether linkage at the *sn*-1 chain, which reduces its local flexibility and therefore minimally interferes with the *sn*-2 chain that assumes its catalytically competent orientation. Notably, both the spatial configuration of the *sn*-2 chain and the binding affinity (~5.3 ± 0.3 kcal/mol) were consistently retained across the studied systems, independently of PEBP1 (Supplementary Fig. 7b), and closely resembled those observed for diacyl-S-ETE-PE^32^ (Supplementary Fig. 7c).

To experimentally validate the dispensability of PEBP1 for 15-LOX-driven oxidation of alkenyl-PE and -PC, we knocked down (KD) PEBP1 in HAEC cells and estimated the levels of oxidized diacyl- and alkenyl-S-ETE species of PE and PC. As expected, we found that the amount of diacyl-S-HpETE-PE was dependent on the presence of PEBP1^32^. In contrast, alkenyl-S-HpETE-PE levels in PEBP1 KD cells were not significantly different from those obtained from WT control cells (Supplementary Fig. 8b). Notably, PEBP1 also did not accelerate peroxidation of alkenyl-S-ETE-PE in a model system (Supplementary Fig. 8a). These data demonstrate that PEBP1 is not essential for the preferential 15-LOX catalyzed oxidation of alkenyl-PE species in cells, in agreement with the results obtained in biochemical models and MD simulation data. Notably, the levels of diacyl-S-HpETE-PC in control cells were low and not different from those obtained from PEBP1-deficient cells (Supplementary Fig. 8b). Alkenyl-S-HpETE-PC species were not detected either in control or in PEBP1 KD cells.

### Exogenous 15-LOX-2/alkenyl-ETE-PE induces ferroptosis in A375 cells

**Fig. 4:**
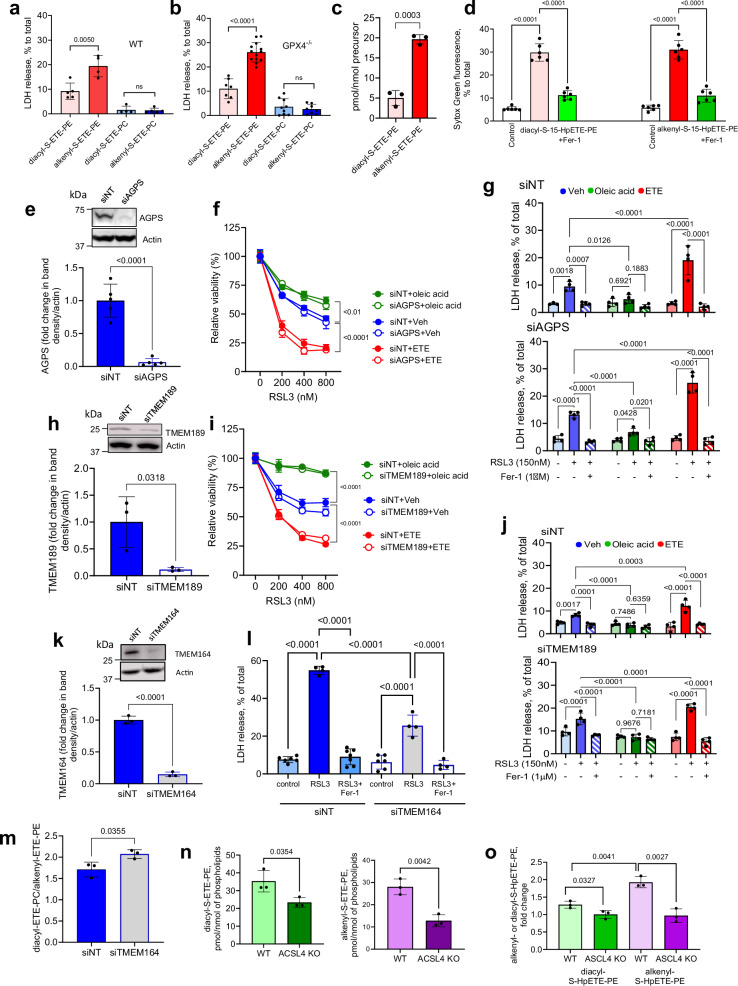
Cell death induced by oxidized ETE-phospholipids. Death induced by 15-LOX-2 and S-ETE-PEs or S-ETE-PCs in WT (**a**), GPX4^−/−^ (**b**) and content of S-HpETE-PEs in GPX4^−/−^ A375 cells (**c**). Mean ± SD, unpaired two-sided Student’s *t*-test, **a** *n* = 4–5 biological samples, (*t* = 4.025, *p* = 0.0050, df = 7), (**b**) *n* = 7-13 biological samples (*t* = 7.968, *p* < 0.0001, df = 18), **c** *n* = 3 biological samples (*t* = 11.08, *p* = 0.0003, df = 4). Death induced by diacyl-S-15-HpETE-PE (left) or alkenyl-S-15-HpETE-PE (right) in GPX4^−/−^ A375 cells (**d**). Mean ± SD, *n* = 5–6 biological samples, one-way ANOVA, (*F* = 109.6, *p* < 0.0001, df = 35). **e** Typical western blot (insert) and quantification of AGPS in A375 cells. Mean ± SD, *n* = 5 biological samples, unpaired two-sided Student’s *t*-test, (*t* = 8.133, *p* < 0.0001, df = 8). **f**, **g** RSL3 induced death of control (siNT) and AGPS KD (siAGPS) cells in the absence and presence of oleic or ETE acids. Mean ± SD, *n* = 4 biological samples, **f** one-way ANOVA (*F* = 97.92, *p* < 0.0001, df = 17), **g** two-way ANOVA: siNT (F[interaction, row, column]=[16.39,60.08, 14.75], df = 26, *p* < 0.0001); siAGPS (F[interaction, row, column]=[41.17,189.4,46.74], df = 27, *p* < 0.0001). **h** Typical western blot (insert) and quantification of TMEM189 in A375 cells. Mean ± SD, *n* = 3 biological samples. Unpaired two-sided Student’s *t*-test (*t* = 3.235, *p* = 0.0318, df = 4). RSL3 induced death of control and TMEM189 KD cells in the absence and the presence of oleic or ETE acids. Mean ± SD, *n* = 4 biological samples, **i** one-way ANOVA (*F* = 409.7, *p* < 0.0001, df = 17), **j** two-way ANOVA: siNT (*F* = 16.67, *p* < 0.0001, df = 27); siTMEM189 (*F* = 28.69, *p* < 0.0001, df = 27). **k** Typical western blot (insert) and quantification of TMEM164 in A375 cells. Mean ± SD, *n* = 3 biological samples, unpaired two-sided Student’s *t*-test (*t* = 20.47, *p* < 0.0001, df = 4). **l** RSL3 induced death of TMEM164 KD A375. Mean ± SD, *n* = 4–7 biological samples, one-way ANOVA: (*F* = 132.1, *p* < 0.0001, df = 30). **m** Ratio of diacyl-ETE-PC to alkenyl-ETE-PE in TMEM164 KD A375 cells. Mean ± SD, *n* = 3 biological samples, unpaired two-sided Student’s *t*-test (*t* = 3.122, *p* = 0.0355, df = 4). Content of S-ETE-PEs in non-treated (**n**) and S-HpETE-PEs in RSL3 treated (**o**) mouse fibroblasts. **n** Mean ± SD, *n* = 3 biological samples, unpaired two-sided Student’s *t*-test, diacyl-S-ETE-PE (*t* = 3.124, *p* = 0.0354, df = 4), alkenyl-S-ETE-PE (*t* = 5.886, *p* = 0.0042, df = 4), **o** Mean ± SD, *n* = 3 biological samples one-way ANOVA (F = 27.31, *p* = 0.001, df = 11). Source data are provided as a Source data file.

Encouraged by the computational and biochemical results that enzymatic 15-LOX induced peroxidation of alkenyl-*sn*-2-ETE-PE could be responsible for the phospholipid selectivity of the ferroptotic process, we employed our previously developed in vitro ferroptosis-induction model in which 15-LOX-2 and PE-substrates were co-incubated with wild type (WT) and GPX4^−/−^ A375 melanoma cells (Fig. 4ab). These experiments revealed that exogenous 15-LOX-2 and either diacyl-S-ETE-PE or alkenyl-S-ETE-PE were able to produce hydroperoxy-products (Fig. 4c) and induce ferroptosis in WT (Fig. 4a) and GPX4^−/−^ A375 cells (Fig. 4b). Importantly, alkenyl-S-ETE-PE was more effective as a pro-ferroptotic substrate than diacyl-S-ETE-PE species. Neither diacyl-S-ETE-PC nor alkenyl-S-ETE-PC was capable of triggering ferroptosis in A375 cells in the presence of 15-LOX-2 (Fig. 4a, b). We further examined whether alkenyl-S-HpETE-PE, the primary product of alkenyl-S-ETE-PE peroxidation, alone can cause cell death. Alkenyl-S-HpETE-PE was biosynthesized and purified using recombinant 15-LOX-2. When we exposed GPX4^−/−^ A375 cells to alkenyl-S-HpETE-PE, we found that the alkenyl species induced ferroptosis to similar levels as were observed in cells treated with diacyl-S-HpETE-PE. (Fig. 4d). This confirms that the differences in pro-ferroptotic activity between alkenyl-ETE-PE and diacyl-ETE-PE were mostly due to the more effective oxidation of the former.

### Oxidation of sn-1-vinyl group of plasmalogens does not affect sensitivity of cells to ferroptosis

Several publications have implied that the effects of alkenyl-PE on ferroptosis depend exclusively on the *sn*-1 vinyl bond, irrespective of the oxidizable PUFA present at the *sn*-2 position^4,33^, while others implicated *sn*-2 PUFA^6^. We were eager to directly address this controversial issue. Alkylglycerone phosphate synthase (AGPS) is required for the synthesis of all ether lipids, including vinyl bond-containing alkenyl-phospholipids^34^. Knock down of AGPS in A375 melanoma cells (Fig. 4e) did not affect RSL3-induced ferroptotic cell death (Fig. 4f). In sharp contrast, cells incubated with oleic acid or ETE—leading to the enrichment of sn-2 positions with non-oxidizable or highly-oxidizable fatty acids—strongly decreased or increased sensitivity to ferroptosis, respectively (Fig. 4f, g). Similarly, KD of TMEM189 (Fig. 4h), which encodes plasmenylethanolamine desaturase, the enzyme that introduces the vinyl bond to alkenyl-PE, had no effect on ferroptosis induced by RSL3 (Fig. 4i). Again, however, oleic acid suppressed and ETE sensitized these cells to ferroptosis triggered with RSL3 (Fig. 4i, j). This corresponds with published data demonstrating that knockout of AGPS or TMEM189 does not affect ferroptosis in several cell lines^6,33^. The depletion of TMEM189 causes proportional increases in alkyl species analogous to the alkenyl species which are depleted, maintaining levels of PUFA-containing alkyl/alkenyl-PE^35^. Together with our data demonstrating that oxidation of the vinyl bond by 15-LOX at the *sn*-1 position of alkenyl phospholipids occurred at an incomparably lower rate than oxidation of *sn*-2 ETE-residue (Fig. 2h, i), these data suggest that the *sn*-2-ETE-residue rather than sn-1-vinyl-group is the major determinant of the pro-ferroptotic sensitivity.

### Oxidation of sn-2-ETE plasmalogens strongly affects sensitivity of cells to ferroptosis

TMEM164 is a cysteine-dependent acyltransferase that incorporates ETE from diacyl-ETE-PC into alkenyl-lysophospholipids, resulting in generation of alkenyl-*sn-2*-ETE-PE species^36^. To experimentally test the contribution of TMEM164-mediated alkenyl-*sn-2*-ETE-PE plasmalogen synthesis towards ferroptosis sensitivity, we used siRNA to knock down TMEM164 in A375 melanoma cells (Fig. 4k). We found that in contrast to AGPS KD, TMEM164 KD decreased sensitivity to RSL3-induced ferroptosis (Fig. 4l). Given the relatively short timeframe of these experiments (<96 h), siRNA knockdown cannot be expected to completely deplete the phospholipid products of these enzymes. Thus, TMEM164 KD may deplete alkenyl-*sn-2*-ETE-PE plasmalogens more effectively than AGPS KD due to remodeling being the predominant pathway of ETE-PE plasmalogen biosynthesis. Indeed, lipidomics analysis confirmed that TMEM164 KD increased the ratio of diacyl-ETE-PC to alkenyl-ETE-PE in A375 cells (Fig. 4m).

Acyl-CoA synthetase long-chain family member 4 (ACSL4) is essential for lipid metabolism and plays a significant role in the regulation of ferroptosis via biosynthesis of ETE-CoA^37^ and subsequent synthesis of ETE-containing PE, including diacyl-S-ETE-PE and alkenyl-S-ETE-PE. Therefore, the knockout of ACSL4 should affect the levels of these two *sn-2*-ETE-PE species in cells. Indeed, our data demonstrated that the contents of both diacyl-S-ETE-PE and alkenyl-S-ETE-PE in ACSL4 knockout (KO) mouse fibroblasts were substantially lower (1.5- and 1.8-fold decreases compared to control cells, respectively) (Fig. 4n). Moreover, while in WT cells treated with RSL3 the levels of both diacyl-S-HpETE-PE and alkenyl-S-HpETE-PE were increased 1.3- and 1.9-fold, respectively, no changes in their levels were detected in ACSL4 KO cells (Fig. 4o). These data suggest that enrichment of alkenyl phospholipids with *sn*-2-ETE sensitizes cells to the production of HpETE-PE and ferroptotic death.

### Endogenous 15-LOX accelerates oxidation and consumption of alkenyl-PE in HAECs

**Fig. 5:**
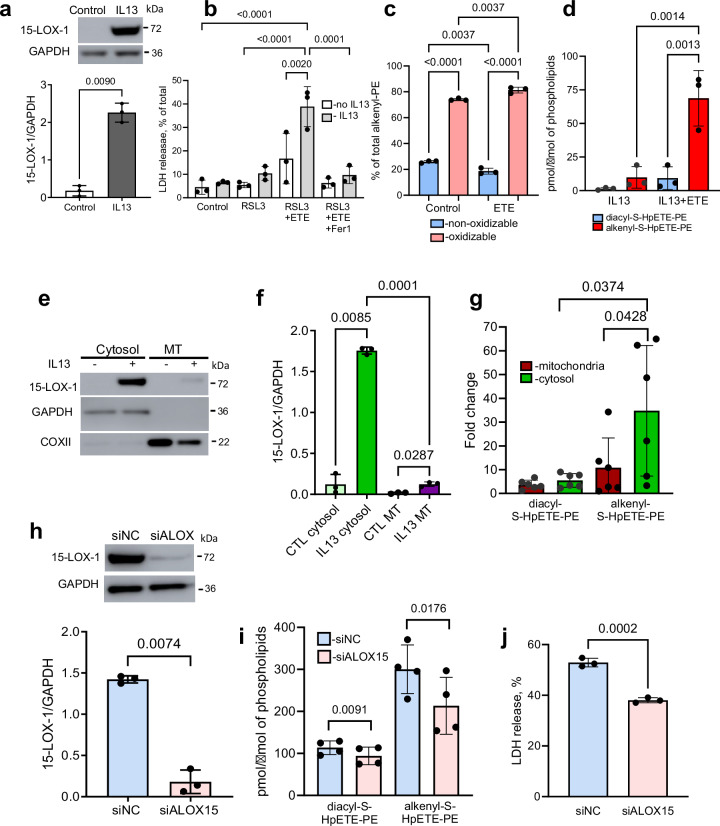
Oxidation of alkenyl-S-ETE-PE induced by 15-LOX-1 in HAECs. **a** Typical western blot (insert) and quantification of 15-LOX-1 in HAECs. Mean ± SD, *n* = 3, each from a different donor, paired two-sided Student’s *t*-test (*t* = 10.50, *p* = 0.0090, df = 2). **b** IL13 sensitizes HAEC to ferroptosis induced by RSL3. Mean ± SD, *n* = 3, each from a different donor, one-way ANOVA (*F* = 13.88, *p* < 0.0001, df = 23). **c** Content alkenyl-PEs in HAECs. Mean ± SD, *n* = 3, each from a different donor, one-way ANOVA (*F* = 1045, *p* < 0.0001, df = 11), non-oxidizable: control vs ETE, oxidizable: control vs ETE (adjusted *p* = 0.0037). **d** Effect of ETE on accumulation of S-HpETE-PEs in IL13 treated cells. Mean ± SD, *n* = 3, each from a different donor, one-way ANOVA: (*F* = 20.61, *p* = 0.0004, df = 11, adjusted p = 0.0014, p = 0.0013). A typical western blot (**e**) and quantitation (**f**) of 15-LOX-1 in cytosol and mitochondria from control and IL13 exposed cells. Mean ± SD, *n* = 3, each from a different donor, one-way ANOVA (*F* = 424.9, *p* = 0.0016, df = 11, adjusted *p* = 0.085, *p* = 0.001, *p* = 0.0287). **g** Changes in the content of S-HpETE-PEs in cytosol and mitochondria from IL13 treated HAECs. Mean ± SD, *n* = 6, each from a different donor, paired two-sided Student’s *t*-test, cytosol: diacyl-S-HpETE-PE vs. alkenyl-S-HpETE-PE (*t* = 2.813, *p* = 0.0374, df = 5); alkenyl-S-ETE-PE: cytosol vs. mitochondria (*t* = 2.700, *p* = 0.0428, df = 5). **h** Typical western blot (insert) and quantification of 15-LOX-1 in siNC (control) and siALOX15 (15-LOX-1 KD) IL13 treated HAECs. Mean ± SD, *n* = 3, each from a different donor, paired two-sided Student’s *t*-test (*t* = 11.53, *p* = 0.0074, df = 2). **i** Content of S-HpETE-PEs in 15-LOX-1 deficient HAECs exposed to IL13. Mean ± SD, *n* = 4, each from a different donor, paired two-sided Student’s t-test, alkenyl-S-HpETE-PE: siNC vs. siALOX15 (*t* = 4.763, *p* = 0.0176, df = 3), diacyl-S-HpETE-PE: siNC vs. siALOX15 (*t* = 6.042, *p* = 0.0091, df = 3). **j** Cell death induced by RSL3 in IL13 treated control and 15-LOX-1 KD HAECs. Mean ± SD, *n* = 3, each from a different donor, unpaired two-sided Student’s *t*-test, (*t* = 13.03, *p* = 0.0002, df = 4). Source data are provided as a Source data file.

To further investigate the role of intracellular 15-LOX in ferroptosis associated with the peroxidation of alkenyl-phospholipids, we used HAECs in which interleukin 13 (IL13) is known to cause robust expression of 15-LOX-1 (Fig. 5a)^38^. Treatment of HAECs with IL13 in the presence of ETE sensitized these cells to ferroptosis induced by RSL3 (Fig. 5b)^32^. Using LC-MS lipidomics (Supplementary Fig. 9a), we estimated the amounts of diacyl-S-ETE- and alkenyl-S-ETE-PE and respective-PC species in HAECs (Fig.1b, c). Among ETE-PC species, diacyl-S-ETE-PCs were the most abundant (Fig. 1c**)**. Analysis of alkenyl-PEs revealed that they were mainly represented by highly oxidizable PUFA containing species in HAECs (Fig. 5c). Treatment of HAECs with ETE resulted in the increase of oxidizable alkenyl-PE species (Fig. 5c). Exposure of ETE-treated HAECs to IL13 resulted in robust accumulation of the alkenyl-S-HpETE-PE (alkenyl-S-ETE-PE oxidation product) (Fig. 5d). The level of alkenyl-HpETE-PC was not changed in ETE-treated HAECs exposed to IL13 (Supplementary Fig. 9b).

### Cytosolic localization of 15-LOX-1 and peroxidation of alkenyl-PE in HAECs

To determine the major intracellular localization of 15-LOX-catalyzed alkenyl-S-ETE-PE oxidation, we isolated mitochondrial and cytosolic fractions from control HAECs and HAECs exposed to IL13. Western blot analysis showed that 15-LOX-1 was predominantly expressed in the cytosol in response to IL13 (Fig. 5e, f)^38^. The expression of the enzyme in mitochondria was barely detectable (Fig. 5e). Importantly, significant accumulations of alkenyl-S-HpETE-PE (Fig. 5g) as well as alkenyl-O-HpETE-PE (Supplementary Fig. 9c) and alkenyl-P-HpETE-PE (Supplementary Fig. 9d) were detected in the cytosolic fraction versus the mitochondrial fraction isolated from IL13 treated cells. Of note, the absence of significant changes in the levels of alkenyl-S-HpETE-PE in mitochondria is concordant with low expression of 15-LOX-1. While the content of alkenyl-ETE-PE in mitochondria was comparable with that in the cytosol (Fig. 1d, e), the oxidation of alkenyl-ETE-PE was dependent on the level of 15-LOX-1.

### Peroxidation of alkenyl-PE correlates with genetically manipulated expression of 15-LOX and sensitivity to ferroptosis

To explore the sufficiency of 15-LOX for the oxidation of alkenyl-ETE-PE in cells, we performed experiments in which we genetically manipulated the level of 15-LOX-1 in HAECs using siRNA (siALOX15) (Fig. 5h)^38^. We found that in 15-LOX-1 KD HAECs, the levels of diacyl-, alkenyl-S-HpETE-PE (Fig. 5i) and alkenyl-O-HpETE-PE (Supplementary Fig. 9e) species were significantly lower compared to control (siNC) cells. The change in the contents of alkenyl-S-HpETE-PE was more profound compared to that observed for diacyl-species (Fig. 5j). No changes in the levels of hydroperoxy-PC were detected in 15-LOX-1 KD cells (Supplementary Fig. 9f, g). Finally, we demonstrated the relevance of these findings to ferroptosis by exposing 15-LOX-1 KD HAECs to IL13 and subsequently treating with RSL3. We found that 15-LOX-1 KD decreased RSL3-induced cell death in IL13-treated HAECs (Fig. 5j), suggesting that 15-LOX-1 driven peroxidation of alkenyl-ETE-PE promotes ferroptosis execution.

**Fig. 6:**
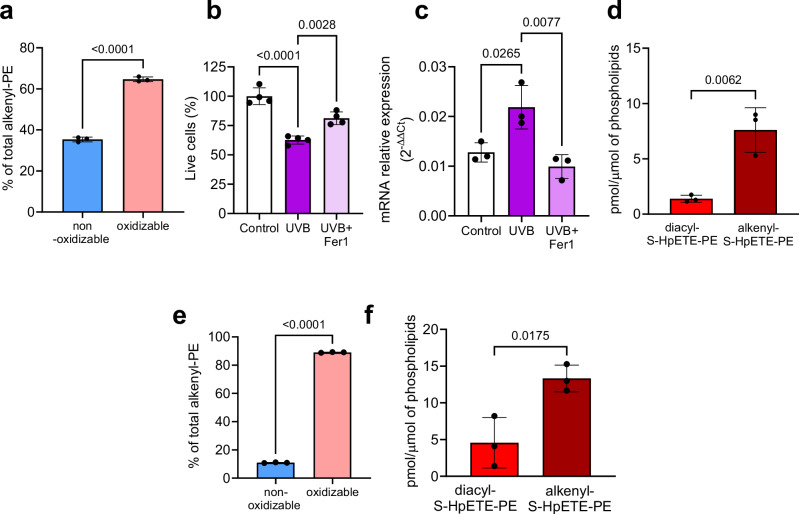
15-LOX-2 associated oxidation of alkenyl-S-ETE-PE in human keratinocytes and human epidermal explants. **a** Content of non-oxidizable and oxidizable alkenyl-PE species in human keratinocytes. Mean ± SD, *n* = 3 biological samples, unpaired two-sided Student’s *t*-test (*t* = 31.14, *p* < 0.0001, df = 4). **b** Percentage of surviving human epidermal keratinocytes (HEKs) 6 hrs after UVB (10 kJ/m^2^) exposure. Cells were pretreated with ferrostatin-1 (Fer-1, 10 μM). Mean ± SD, *n* = 4 biological samples, one-way ANOVA (*F* = 45.23, *p* < 0.0001, df = 11). **c** UVB induced 15-LOX expression in HEKs. Data are presented as mRNA relative expression. Mean ± SD, *n* = 3 biological samples, ordinary one-way ANOVA (*F* = 12.21, *p* = 0.0077, df = 8). **d** Fer-1 sensitive oxidation of diacyl- and alkenyl S-ETE-PE species in HEKs exposed to UVB. Mean ± SD, *n* = 3 biological samples, unpaired two-sided Student’s *t*-test (*t* = 5.273, *p* = 0.0062, df = 4). **e** Content of non-oxidizable and oxidizable alkenyl-PE species in human epidermal explants (HEE). Mean ± SD, *n* = 3 biological samples, unpaired two-sided Student’s *t*-test (*t* = 423, *p* < 0.0001, df = 4). **f** Fer-1 sensitive oxidation of diacyl- and alkenyl-S-ETE-PEs in human HEE exposed to UVB (10 kJ/m^2^). Mean ± SD, *n* = 3 biological samples, unpaired two-sided Student’s *t*-test (*t* = 3.900, *p* = 0.0175, df = 4). Source data are provided as a Source data file.

To examine the essentiality of 15-LOX-2 for peroxidation of alkenyl-ETE-PE, we employed a human keratinocyte model. Lipidomics analysis of alkenyl-PE species revealed that in human epidermal keratinocytes (HEK), alkenyl-PE were mainly represented by highly oxidizable species containing PUFA (Fig. 6a). Taking into account that in HEK UVB exposure induces ferroptosis (Fig. 6b) associated with an increase in 15-LOX-2 expression (Fig. 6c), we comparatively assessed the contents of diacyl- and alkenyl-S-HpETE-PE in these cells. We found that UVB exposure induced five-times higher ferrostatin-1 (Fer-1)-sensitive production of alkenyl-S-HpETE-PE compared to diacyl-S-HpETE-PE (Fig. 6d). In human epidermal explants, PUFA alkenyl-PEs were predominant species as well (Fig. 6e). A similar selective sensitivity to peroxidation was observed in UVB-exposed human epidermal explants ex vivo. In this model, elevated levels of alkenyl-S-HpETE-PE versus diacyl-S-HpETE-PE were documented by LC-MS analysis (Fig. 6f).

### Role of 15-LOXs in oxidation of alkenyl-PEs in disease conditions

Given the importance of aberrant and massive PE peroxidation as a pro-ferroptotic pathogenic factor, we chose to explore the role of 15-LOX in peroxidation of alkenyl-ETE-PE in three disease conditions: (i) traumatic brain injury (TBI), (ii) PMN-MDSC in the tumor microenvironment of growing Lewis lung carcinoma (LLC), and (iii) asthma.

**Fig. 7:**
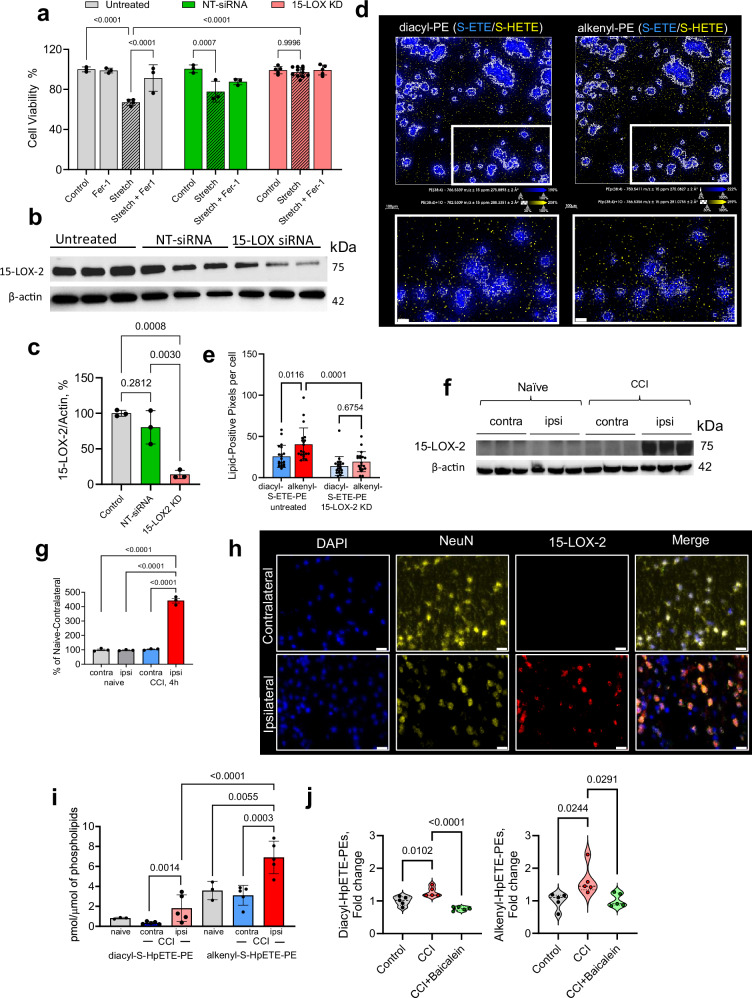
Assessment of oxidized S-ETE-PEs in stretched HT22 cells and mouse brain after CCI. **a** 15-LOX-2 KD attenuates neuronal death induced by mechanical stretch injury. Mean ± SD, *n* = 3–11 biological samples, one-way ANOVA, (*F* = 15.92, *p* < 0.0001, df = 42, adjusted *p* = 0.0007). Typical western blot (**b**) and quantification (**c**) of 15-LOX-2 in HT22 cells. Mean ± SD, *n* = 3 biological samples, one-way ANOVA, (*F* = 30.16, *p* = 0.0007, df = 8, adjusted p = 0.0008, *p* = 0.0030). **d** timsON MALDI-MSI of HT22 cells (untreated/stretch), enabling separation of isobaric species by collision cross section (CCS). Left: diacyl-S-ETE-PE (PE38:4, *m*/*z* 766.5382 ± 15 ppm, blue; CCS = 273.87 ± 2 Å) overlaid with diacyl-S-HETE-PE (PE38:4 + 1[O], *m*/*z* 782.5331 ± 15 ppm, yellow; CCS = 288.46 ± 2 Å). Right: alkenyl-S-ETE-PE (PE38:4, *m*/*z* 750.5411 ± 15 ppm, blue; CCS = 273.97 ± 2 Å) overlaid with alkenyl-S-HETE-PE (PE38:4 + 1[O], *m*/*z* 766.5356 ± 15 ppm, yellow; CCS = 278.20 ± 2 Å). Scale bars: 100 µm; bottom insets: zoomed images with a 50 µm scale bar, showing higher-resolution lipid distribution at the single-cell level. **e** Quantification of S-HETE-PEs at the single-cell level. The number of lipid-positive pixels per cell was measured within SCiLS Lab in untreated/stretch and 15-LOX-2 KD/stretch HT22 cells. Bars show mean pixel count ± SD (*n* = 20 cells). One-way ANOVA (*F* = 12.18, *p* < 0.0001, df = 79, adjusted *p* = 0.0116, *p*-0.0001). **f** Typical western blot and (**g**) quantification of 15-LOX-2 in naïve and injured ipsilateral (ipsi) and uninjured contralateral (contra) cortex at 4 h after CCI. *n* = 3 mice per group, Mean ± SD, one-way ANOVA, (*F* = 359.3, *p* < 0.0001, df = 11). **h** 15-LOX-2 expression in the brain after CCI. Confocal images (Zeiss LSM900, 40X objective) of ipsilateral pericontusional area. Dual immunofluorescence staining: anti-NeuN (yellow), anti-15-LOX-2 (red) antibodies, and DAPI to stain nuclei (blue). Representative merged images show co-localization of NeuN and 15-LOX-2 4 h after CCI in the ipsilateral region. Scale bars are 40 μm. *n* = 3 independent experiments. **i** Contents of S-HpETE-PEs in mouse brain after CCI. *n* = 3 naive mice, *n* = 5 CCI mice, Mean ± SD, one-way ANOVA (*F* = 22.48, *p* < 0.0001, df = 25, adjusted *p* = 0.0055, *p* = 0.0003), unpaired two-sided Student’s *t*-test: diacyl-S-HpETE-PEs contra vs ipsi (*t* = 5.606, *p* = 0.0014, df = 6). **j** Baicalein suppresses CCI induced generation of HpETE-PEs in ipsilateral cortex of mouse brain. *n* = 5 mice per group. One-way ANOVA: diacyl-ETE-PEs (*F* = 21.81, *p* = 0.0001, df = 14, adjusted *p* = 0.0102); alkenyl-ETE-PEs (*F* = 6.091, *p* = 0.01149, df = 14, adjusted p = 0.0244, p = 0.0291). Source data are provided as a Source data file.

To examine the effect of 15-LOX-2 on neuronal death and PE oxidation, we utilized a well-established in vitro model of TBI - neuronal mechanical stretch injury - that leads to ferroptotic death rescuable by Fer-1^39^ (Fig. 7a). In culture, neuronal (HT22) cells contain relatively high level of 15-LOX-2 (Fig. 7b). Importantly, KD of 15-LOX-2 in these cells (Fig. 7b, c) increased resistance to cell death induced by mechanical stretch (Fig. 7a). To examine the effect of 15-LOX-2 on the generation of stretch-induced PE oxidation products, we employed imaging mass spectrometry. We found that ETE-PE species underwent marked oxidation upon injury, which was attenuated by Fer-1 as well as 15-LOX-2 KD (Supplementary Fig. 10a). To further investigate whether there were differences between alkenyl versus diacyl ETE-PE oxidation, we utilized ion mobility LC-MS and observed higher levels of alkenyl-HETE-PE than diacyl-HETE-PE after stretch injury in a 15-LOX-2 dependent manner (Fig. 7de and Supplementary Fig. 10b, c).

Using a mouse model of controlled cortical impact (CCI), we revealed a link between the changes in 15-LOX-2 expression and the accumulation of hydroperoxy-species of diacyl- and alkenyl-ETE-containing phospholipids in the brain. Expression of 15-LOX-2 was elevated in the ipsilateral pericontusional cortical area >3.5 times versus the uninjured contralateral cortex or naïve control cortex (Fig. 7f, g) and this effect occurred within the first 4 hrs after CCI, (primarily in neurons) (Fig. 7h). We found that CCI caused a significant increase of the contents of diacyl- and alkenyl-S-HpETE-PE peroxidation products in ipsilateral areas of the brain whereby the accumulation of alkenyl-S-HpETE-PE was markedly higher than that of diacyl-S-HpETE-PE (Fig. 7i). These findings were not limited only to S-HpETE-species, as analyses of O-HpETE-PE and O-HpETE-PC species yielded similar results (Supplementary Fig. 10e). The contents of diacyl-S-HpETE-PC, alkenyl-S-HpETE-PC, diacyl-O-HpETE-PE or alkenyl-O-HpETE-PC species were not changed upon CCI (Supplementary Fig. 10d–f).

To further explore the role of 15-LOX-2-dependent ferroptosis in mechanisms of CCI-induced injury, we employed a ferroptosis inhibitor that targets 15-LOX activity. Although the anti-ferroptotic compound, Fer-1, is an effective 15-LOX inhibitor^40^, it has limited bioavailability and poor blood brain barrier penetration^41–43^. For this reason, we employed an alternative inhibitor, a polyphenolic flavonoid compound, baicalein (5,6,7-trihydroxyflavone), known to effectively suppress 15-LOX activity^40^. Post-injury treatment with baicalein (50 mg/kg, i.p.) attenuated CCI induced accumulation of alkenyl-HpETE-PE and diacyl-HpETE-PE (Fig. 7j), which has been associated with improved spatial memory acquisition versus vehicle-treated controls^39^.

**Fig. 8:**
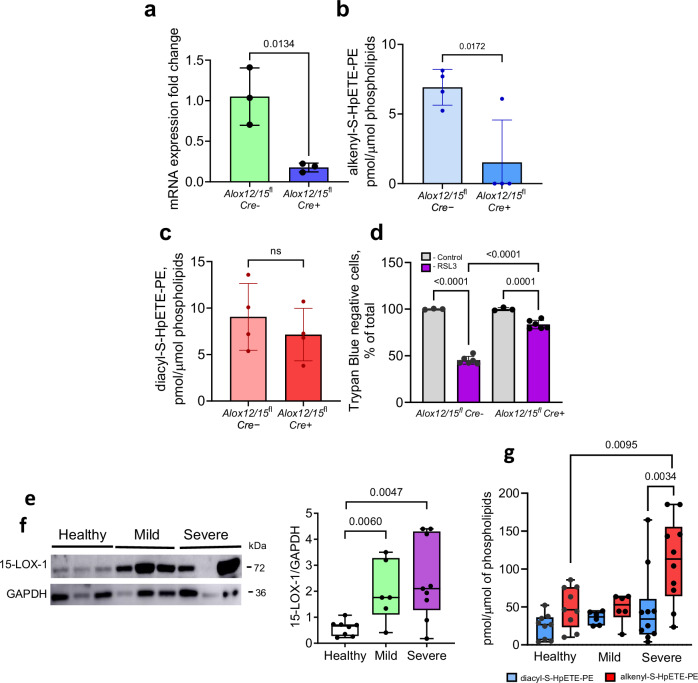
Assessment of diacyl- and alkenyl-S-HpETE-PE in PMN-MDCs from tumor bearing mice and HAEC from asthma patients. **a** Expression of Alox12/15 by qRT-PCR in PMN-MDSCs from *Alox12/15*^*fl*^*Cre*^*−*^ and *Alox12/15*^*fl*^*Cre*^*+*^ Lewis lung carcinoma (LLC) bearing mice. Mean ± SD, *n* = 3 biological samples (PMNs from three different mice), unpaired two-sided Student’s *t*-test (*t* = 4.229, *p* = 0.0134, df = 4). **b** Content of alkenyl-S-HpETE-PE in tumor PMN-MDSC obtained from *Alox12/15*^*fl*^*Cre*^*−*^ and *Alox12/15*^*fl*^*Cre*^*+*^ LLC bearing mice. Mean ± SD, *n* = 3 biological samples (PMNs from three different mice), unpaired two-sided Student’s *t*-test (*t* = 3.264, *p* = 0.0172, df = d). **c** Content of diacyl-S-HpETE-PE in tumor PMN-MDSC obtained from *Alox12/15*^*fl*^*Cre*^*−*^ and *Alox12/15*^*fl*^*Cre*^*+*^ tumor bearing mice. Mean ± SD, *n* = 3 biological samples (PMNs from three different mice), unpaired two-sided Student’s t-test revealed no significant (ns) differences. **d** RSL3 induced death of bone marrow PMN-MDSC isolated from the LLC tumor bearing *Alox12/15*^*fl*^*Cre*^*−*^ and *Alox12/15*^*fl*^*Cre*^*+*^ mice (*n* = 3 per group for untreated; *n* = 6 per group for RSL3 treated cells). Mean ± SD, one-way ANOVA (*F* = 222.8, *p* < 0.0001, df = 17). **e** Western blot of 15-LOX-1 and (**f**) levels of 15-LOX-1 in HAECs obtained from healthy subjects (n = 9) and patients with mild (*n* = 6) and severe (*n* = 10) asthma. Boxplot shows the median (center line), 5th-95th percentiles (box), and minimum/maximum values (whiskers) of data. Unpaired two-sided Student’s *t*-test, healthy vs mild asthma (t = 3.330, *p* = 0.0060, df = 12) and healthy vs severe asthma (*t* = 3.313, *p* = 0.0047, df = 15). **g** Content of diacyl- and alkenyl-S-HpETE-PE in HAECs obtained from healthy subjects (*n* = 9) and patients with mild (*n* = 6) and severe (n = 10) asthma. Boxplot shows the median (center line), 5th–95th percentiles (box), and minimum/maximum values (whiskers) of data. Unpaired two-sided Student’s *t*-test, alkenyl-S-HpETE-PE: healthy subjects vs patients with severe asthma (*t* = 2.923, *p* = 0.0095, df = 17). Paired two-sided Student’s *t*-test, diacyl-S-HpETE-PE vs alkenyl-S-HpETE-PE in severe asthma (*t* = 3.936, *p* = 0.0034, df = 9). Source data are provided as a Source data file.

Ferroptotic PE peroxidation in innate immune cells is an important mechanism of immunosuppression in the tumor microenvironment^44^. Assuming that 15-LOX-catalyzed peroxidation of alkenyl-PUFA-PE may be accountable, at least in part, for this process, we examined the levels of hydroperoxy-derivatives of alkenyl-S-ETE-PE in PMN myeloid-derived suppressor cells (PMN-MDSC) isolated from tumors of Lewis lung carcinoma (LLC)-bearing *Alox12/15*^*fl*^*Cre*^*+*^ and *Alox12/15*^*fl*^*Cre*^*−*^ mice. No changes in the contents of diacyl- and alkenyl-S-ETE-PE species were detected in 12/15-LOX-deficient animals (Supplementary Fig. 10g). However, lower amounts of alkenyl-S-HpETE-PE were correlated with a lower expression of LOX-2 (12/15-LOX) in PMN MDSC from *Alox12/15*^*fl*^*Cre*^*+*^ *versus Alox12/15*^*fl*^*Cre*^*−*^ LLC tumor bearing mice (Fig. 8a, b). This suggests that the selectivity for alkenyl-PE oxidation in PMN-MDSC is driven by 15-LOX-2 catalysis. No significant differences in the content of diacyl-HpETE-PE in PMN MDSC between *Alox12/15*^*fl*^*Cre*^+^ *versus Alox12/15*^*fl*^*Cre*^−^ mice were detected (Fig. 8c). Finally, we demonstrated that PMNs isolated from *Alox12/15*^*fl*^ *Cre*^*-*^ mice were more sensitive to cell death induced by RSL3 compared to PMNs from *Alox12/15*^*fl*^ *Cre*^*+*^ mice (Fig. 8d), demonstrating that alkenyl-S-HpETE-PE, among other 15-LOX-2 peroxidation products, sensitize PMN-MDSC to ferroptosis.

Considering that 15-LOX-1 is involved in the pathogenesis of asthma^38^ we obtained HAEC cells from healthy subjects and patients with mild and severe asthma. We established a correlation between 15-LOX-1 expression and asthma severity (Fig. 8e, f)^38^. Significantly elevated levels of alkenyl-, but not diacyl-S-HpETE-PE species were found in cells obtained from patients with severe asthma versus healthy controls (Fig. 8g). Furthermore, we established a strong positive correlation (*r* = 0.62, *p* < 0.002) between alkenyl-S-HpETE-PE and FeNO (fractional exhaled nitric oxide) (Supplementary Fig. 10h), the most common biomarker of Type-2 airway inflammation in asthma^45^. Of relevance, however, 7 of the severe asthma patients were on T2-targeted monoclonal antibody therapy, with the differences by asthma severity or FeNO not impacted by their use. These data further demonstrate that the 15-LOX preference for peroxidation for alkenyl-S-ETE-PE relative to diacyl-S-ETE-PE is important in contexts beyond ferroptosis.

## Discussion

### Abundance, structure, functions and peroxidation of ether phospholipids

Ether phospholipids are found across domains of life and are typically present as plasmalogen (alkenyl) species in mammals. Despite their abundance, the function of these lipids remains elusive, though the lack of an sn-1 ester group allows alkyl/alkenyl species to pack more tightly in membranes^3^ and preferentially react with certain enzymes^36^. Another popular concept states that oxidation of the characteristic sn-1 vinyl ether bond of plasmalogens confers antioxidant properties^14,46–48^. Evidence for such a sacrificial antioxidant role is lacking in living systems, however^18,49,50^, and this role is also at odds with the known enrichment of mammalian plasmalogens with oxidizable sn-2 PUFA^36^. This necessitates further studies of mechanisms of plasmalogens action, particularly LC-MS based structural characterization of alkenyl peroxidation products in cell contexts with dysregulated redox metabolism, such as ferroptosis.

Ferroptotic lipid peroxidation is driven primarily by the availability of polyunsaturated substrates, especially PUFA‑PEs. Enzymatic formation of 15-HpETE-PE is an obligatory feature of the initiation of ferroptosis^10,20^. Three major conditions are required for the completion of the ferroptotic program: (i) sufficiency of oxidizable PUFA-PL, (ii) redox-active metal, (iii) deficiency of redox, mostly-thiol-dependent, control over HOO-PE via their reduction to stable HO-derivatives. Here, we establish that alkenyl-ETE-PEs, quantitatively the most abundant ETE-PE molecular species, are utilized as preferred peroxidation substrates for both isoforms of 15-LOX (15-LOX-1 and 15-LOX-2). This results in the accumulation of the respective 15-HpETE-PE products in cells and tissues under pro-oxidant conditions, as we demonstrate in several diseases, including acute brain and skin injuries, asthma, and cancer^38,44,51–53^.

Several recent genetic studies have indicated that alkyl/alkenyl-phospholipids are pro-ferroptotic^4,6,16,17^, likely due to the enzymatic enrichment of alkenyl-PE with oxidizable ETE at the *sn*-2 position in mammals^36^. In contrast, another group of reports has shown that peroxisomal and ether-phospholipid *deficiencies* increase cellular vulnerability to ferroptosis in *C. elegans*^5,18^, where alkenyl-PE are not enriched in ETE^54^. Finally, yet another group of publications states that ether phospholipids do not affect ferroptosis at all^33^. It is likely that these apparently inconsistent results reflect different views on the importance of pro-ferroptotic peroxidation of the *sn*-2 PUFA-acyl chain versus “antioxidant” oxidation of the *sn*-1 vinyl group in alkenyl ether phospholipids. Indeed, the vinyl ether bond of plasmalogens may be oxidized^46^, thereby preventing lipid oxidation in some contexts^14,47,48^. This points to an essential but overlooked factor in the plasmalogen’s structure strongly affecting their pro-ferroptotic function and necessitates LC-MS based structural characterization of alkenyl/acyl-PE peroxidation products generated during ferroptosis. The current work establishes that this factor is the presence of oxidizable sn-2-PUFA/ETE residue in alkenyl-PE, rather than of the sn-1 vinyl ether bond. This conclusion is directly supported by the fact that alkenyl-sn-2-HpETE-PE represents the main product of alkenyl-sn-2-ETE-PE oxidation by 15-LOX in ferroptosis.

The peroxidation process is highly selective, as the more abundant diacyl-PUFA-PC species are not engaged in 15-LOX-catalyzed peroxidation reactions leading to ferroptotic death, and minor PE species such as di-PUFA-PE can also be selectively peroxidized^31^. Interestingly, peroxidation of diacyl-ETE-PE by 15-LOX is tightly regulated by a scaffold protein, PEBP1, which forms a complex with the enzyme, increasing the reaction rate and the yield of 15-hydroperoxy isomer of the product^32^. The markedly faster peroxidation of alkenyl-ETE-PEs is insensitive to PEBP1, similar to the even more efficient peroxidation of di-PUFA-PE^31^. PEBP1 prevents the *sn*-1 chain of diacyl-ETE-PE from entering the catalytic site and disturbing *sn*-2 peroxidation, but in alkenyl-ETE-PEs, such stabilization is not required: lacking the ester linkage, the *sn*-1 chain does not bind PEBP1 but instead associates with the 15-LOX surface, allowing peroxidation to proceed unhindered. Combined, these results indicate that the high selectivity of the enzymatic peroxidation by 15-LOXs may define the overall selectivity of lipid peroxidation in ferroptosis. Subsequent stages of the lipid peroxidation process—loosely-bound Fe-catalyzed radical-mediated formation of oxidatively-truncated electrophilic products and their interactions with nucleophilic sites in proteins yielding adducts—do not require enzymatic regulation and proceed as random reactions. It is tempting to speculate that enzymatic, 15-LOX-catalyzed PE peroxidation and formation of 15-HpETE-PE products and their reduction to the respective alcohols by GPX4/GSH represents a still reversible segment of the ferroptotic process. However, transition metal-catalyzed generation of radical intermediates and electrophilic secondary products capable of forming adducts with proteins represents a point of no return, leading to the cell’s demise.

The enzymes of de novo plasmalogen biosynthesis (e.g., AGPS, TMEM189) are not known to have selectivity towards ETE or other PUFA. To our knowledge, TMEM164 is the first plasmalogen biosynthetic enzyme that has been shown to have such selectivity^36^, suggesting that the sn-2 PUFA enrichment of alkenyl-PE is a result of acyl chain remodeling. While CoA-independent trans-acylase activity was first reported in 1983^55^, TMEM164 was identified as the gene encoding this enzyme only two years ago^36^. As a result, there are few studies on TMEM164 regulation. Nonetheless, early reports suggest that inflammatory cytokines may increase CoA-independent trans-acylase activity^56^, while others found that substrate availability may regulate the activity of this enzyme^57^, implying that it is constitutively active. Similarly, sufficient levels of ETE donors (e.g., diacyl-ETE-PC) are required for TMEM164 to synthesize alkenyl-ETE-PE. The neutral effects of AGPS and TMEM189 knockdown in A375 melanoma cells suggest that while alkenyl-ETE-PE may be preferred substrates for enzymatic oxidation, they are not necessary for ferroptosis. It is possible that compensatory effects of alternative types of sn-2-ETE-PE - alkyl-, alkenyl- or diacyl- can maintain ferroptosis sensitivity in some cells when ether lipids are depleted. Indeed, known contents of oxidizable PUFA-PE/ETE-PE substrates (e.g., diacyl-ETE-PE^10,58^ are massively in excess of what is required for ferroptosis execution. The abundance of these substrates is maintained by alternative enzymatic systems, including ACSL4, LPCAT3, and FATP2 sufficiently contributing to the pool of oxidizable PUFA-PE^59^. We demonstrated that 15-LOX-2 induced oxidation of alkenyl-S-ETE-PE almost two times more effectively than diacyl-S-ETE-PE in liposomes. This preferential peroxidation of alkenyl-S-ETEPE was not observed when peroxidation of phospholipids was initiated non-enzymatically by a lipophilic initiator of peroxyl radicals, AMVN. Interestingly, from the known pro-ferroptotic LOX substrates, only di-PUFA-PE (di-ETE-PE) is peroxidized significantly faster than sn-2-ETE plasmalogens^31^. Further studies will reveal the potentially unique role of sn-2-PUFA-PE in ferroptosis.

### Organelle-specific initiation and spread of ferroptosis

The ferroptotic process can be initiated in different organelles dependently on the sufficiency of oxidizable substrates, catalysts, and dysregulation of controlling mechanisms, including thiols, iNOS/NO^•^, and oxidoreductases coupled with endogenous antioxidants (e.g., CoQ10-H2). Conceptually and experimentally, ferroptosis was discovered in 2012^60^ and first linked to ER^10^. Subsequent work demonstrated that essentially all organelles—endoplasmic reticulum^10,61^, Golgi apparatus^62^, mitochondria^38,63,64^, lysosomes^65^, peroxisomes^6^, and nucleus^66^—may be involved in the induction and execution of ferroptotic cell death depending on the stimulus^66^. Mitochondria are one of the major sites for iron utilization and production of reactive oxygen species, fueling the lipid peroxidation process that may, in some cells, define ferroptosis^30^. Ether lipids, including alkenyl-PE species, are crucial for mitochondrial membrane structure and redox balance^67^ and comprise up to 40% of the total mitochondrial PE pool^68^. In addition to de novo synthesis, PE can also be formed in the inner mitochondrial membrane through decarboxylation of PS^69^. However, PS decarboxylase primarily uses PS as its substrate to produce diacyl-PE, not alkenyl-PE^70^. It has been demonstrated that ferroptosis is associated with dramatic morphological changes of mitochondria including membrane rupture^71^ and generation of pro-ferroptotic oxygenated PE species in mitochondria^63^.

Our prior work showed that in H9c2 cardioblasts, RSL3 results in generation of oxygenated PE in mitochondria and mitochondrial injury within 1 h, culminating in ferroptotic death within 3–4 h vs tens of hrs in other cell types^52,63,72^. Moreover, 15-LOX levels and activity are significantly upregulated in mouse cardiomyocyte mitochondria during stress conditions like ischemia-reperfusion, heart failure, and chemotherapy-induced cardiotoxicity. Furthermore, increased 15-LOX expression/activity drives mitochondrial dysfunction, promotes ferroptosis, and causes mitochondrial damage^73^. Although plasmalogen biosynthesis begins in peroxisomes and these organelles represent one of the major producers of reactive oxygen species^74^, unbiased explorations have not implicated peroxisomes as a unique common initiation site of pro-ferroptotic lipid peroxidation^75^. Exploring the ether lipids of peroxisomes and ER during ferroptosis would be an important topic of future studies. Recent work highlights mitochondria associated membranes^76^ and plasma membrane-associated ER^77^ as the intracellular compartments where the ferroptotic process begins. Oxidation of PE by 15-LOX occurs in the cytosol and mitochondria, where 15-LOX is bound to intracellular membranes^78^. Notably, not only intracellular but extracellular initiation of ferroptosis has been documented^31^, thus supporting the concept of a non-cell autonomous nature of ferroptosis^79^. The specificity and effectiveness of different hydroperoxy-phospholipids as inducers of ferroptosis are also not sufficiently studied, although a variety of individual molecular species of hydroperoxy-PE seem to be universally effective^31^. Lately, hydroperoxy-derivatives of other phospholipids, like hydroperoxy-PA, have also been found to exert pro-ferroptotic activity^80^.

Together, these results demonstrate that (i) the oxidizability of PE *sn*-2 acyl chains is the key determinant of ferroptosis sensitivity and (ii) while they are inferior 15-LOX substrates in WT cells, diacyl-ETE-PE are sufficiently oxidizable to uphold ferroptosis capacity in the absence of ether species.

### Pathophysiological relevance and therapeutic implications

Various studies link 15-LOX to ferroptosis in several pathophysiological contexts, including asthma^32,81,82^, cancer^83^, neurodegeneration^84,85^, ischemia-reperfusion^73,86^, and irradiation damage^87^. Initial redox lipidomics analysis suggested that 15-LOX drives lipid peroxidation, particularly PE oxidation^10^, resulting in activation of ferroptotic cell death^88^. In this work, we established that alkenyl-ETE-PEs, quantitatively the most abundant ETE-PE molecular species, are utilized as preferred peroxidation substrates for both isoforms of 15-LOX (15-LOX-1 and 15-LOX-2). This results in the accumulation of the respective 15-HpETE-PE products in cells and tissues under pro-oxidant conditions, as we demonstrated in several diseases, including acute brain injury, skin injury, asthma, and cancer^38,44,51–53^. Previous studies by us^32,39^ and others^89,90^ have shown markedly increased 15-LOX-2 expression in brain tissue after TBI. Indeed, the very low level of 15-LOX in healthy brain (particularly in neurons), increases many-fold after acute brain injury (CCI)^39,91^. Thus, targeting pro-ferroptotic 15-LOX activity may be protective against CCI induced injury. Indeed, a known 15-LOX inhibitor and free radical scavenger, baicalein, was effective in preventing CCI induced injury at biochemical and behavioral levels^39^.

Similar observations of increased 15-LOX-2 expression were reported here in the context of UV-induced skin injury. While UVB exposure can have multiple effects on the skin, selective LOX-catalyzed peroxidation of alkenyl-ETE-PE (and to some extent diacyl-ETE-PE) seems to be an important contributing pathogenic injury factor realizing its effects through skin dishomeostasis and inflammation^72^. Importantly, the upregulation of 15-LOX with UVB exposure has been documented^92^.

In the context of asthma, it has been shown that inhibition of 15-LOX-1 directly improves airway epithelial cell redox balance and lessens autophagic processes. However, no human studies of specific inhibition of 15-LOX-1 linked ferroptotic processes have been performed. Interestingly, vitamin E (γ-tocopherol), shown to inhibit 15-LOX and ferroptosis in vitro^10^, has been reported to lower eosinophilic inflammation and mucus production in asthmatic patients^93^. Additionally, we previously established an association of airway epithelial cell expression of 15-LOX-1 with disease severity^94,95^. Furthermore, inhibition of 15-LOX-1 directly improves airway epithelial cell redox balance and lessens autophagic processes^38,82,95^. Here we report a strong positive correlation between the 15-LOX product alkenyl-S-HpETE-PE levels with FeNO•, a common biomarker for Type 2 airway inflammation in asthma^32,45^. Together, these findings suggest that 15-LOX-1 driven peroxidation of alkenyl-ETE-PE may contribute to asthma pathogenesis.

Our work has demonstrated that PE-plasmalogens play an important role in regulation of ferroptosis. While sn-2-PUFA-PE plasmalogens sensitize cells to ferroptosis, non-oxidizable *sn-2* saturated or mono-unsaturated PE-plasmalogens are negative regulators of the ferroptotic process. Because these propensities of plasmalogens are linked to their metabolism by 15-LOX, it will be important to determine whether other LOXs, e.g., 5-LOX and 12-LOX, display selectivity towards plasmalogen PUFA-PE

In conclusion, the multistage process of ferroptotic lipid peroxidation is strongly regulated by the availability of polyunsaturated substrates and specific classes of phospholipids, particularly PUFA-PEs. Among them, PUFA-PE plasmalogens are preferred substrates due to more effective enzymatic peroxidation of their *sn*-2 chain by 15-LOX^10,31,80^. While the *sn*-1 vinyl bond of plasmalogens can be oxidized by 15-LOX, the efficiency of this reaction is more than two orders of magnitude lower than peroxidation of *sn*-2-ETE-PE. Thus, the so-called “antioxidant” function of plasmalogens is not realized as a mechanism of 15-LOX-driven ferroptosis regulation. Pro-ferroptotic enzymatic generation of hydroperoxy-PUFA-PE from the plasmalogen *sn*-2-PUFA precursors may be an alternative pathway for the biosynthesis of pro- and anti-inflammatory lipid mediators^10^. A specific feature of this pathway includes initial peroxidation of PUFA-residues esterified into PUFA-PE plasmalogens. Subsequent hydrolysis of thus-formed oxygenated products may lead to the release of free oxygenated PUFA as lipid mediators. These results suggest that PE plasmalogens function as selective depots for substrates of phospholipid peroxidation or lipid mediator synthesis, ascribing a previously unknown function to these long elusive lipids.

## Methods

### Model experiments

Liposomes (100 µM DOPC/100 µM diacyl-S-ETE-PE or 100 µM DOPC/100 µM alkenyl-S-ETE-PE) were prepared by extrusion through a membrane with 100 nm pores in 25 mM HEPES buffer (pH 7.4) to a concentration of 100 μM in DTPA (transition metal chelator) using a mini-extruder (Avanti Polar Lipids).

### Protein expression and purification

Full-length human PEBP1 was cloned into a pET21-derived bacterial expression plasmid (EMD Millipore, Billerica, MA) modified to express PEBP1 with N-terminal His 10-and mRuby 2 tags. PEBP1 construct was cloned into the modified pET21-mRuby2 vector by Gibson Assembly (New England Biolabs, Billerica, MA) using primers with homology at the upstream (sense) NdeI site (5′-GGTCTGAGGGGATACACTCA TATG-3′) and downstream (antisense) EcoRI site (5′-GCTTGTCGA CGGAGCTCGAATTC-3′) of the vector. Before performing experiments, PEBP1 was desalted into 5 mM Bis-Tris (pH 6.5), 25 mM NaCl.

Lipoxygenases, 15-LOX-1 and 15-LOX-2, were expressed as N-terminal His6-tagged proteins and were purified via immobilized metal affinity chromatography (IMAC) using an Ni-NTA resin for 15-LOX-1 and 15-LOX-2. The protein purity was evaluated by SDS-PAGE analysis and was found to be greater than 90%.

### Oxidation of diacyl-S-ETE-PE and alkenyl-S-ETE-PE induced by 15-LOX-1 or 15-LOX-2 in a model system

Liposomes (100 µM DOPC/100 µM diacyl-S-ETE-PE or 100 µM DOPC/100 µM alkenyl-S-ETE-PE) were incubated in the presence of either human 15-LOX-2 (0.4 µM) or 15-LOX-1 (0.4 µM) and 0.5 µM 9-HpODE in 25 mM HEPES buffer (pH 7.4) at 37 °C. At the end of incubation, lipids were extracted and analyzed by LC/MS. In some experiments, PEBP1 (0.4 µM) was used.

### ESR measurements

Mixture of vesicles (650 µM DOPC/325 µM diacyl-S-ETE-PE or 650 µM DOPC/325 µM alkenyl-S-ETE-PE) with 2.6 µM 15-LOX from Glycine max and 80 µM ascorbate in 25 mM borate buffer, pH 9.0, was used. ESR spectra were recorded on a JEOLREIX spectrometer with 100 kHz modulation (JEOL, Kyoto, Japan) at 35 °C under N_2_ conditions in gas-permeable Teflon tubing (0.8 mm internal diameter, 0.013 mm thickness, Alpha Wire Corporation, Elizabeth, NJ). The tubing was filled with 90 μl of sample, folded doubly, and placed in an open 3.0 mm internal diameter EPR quartz tube. Ascorbyl radical spectra were recorded at 335.535 mT, center field; 2.5 mT, sweep width; 10 mW, microwave power; 0.083 mT, field modulation; 10^3^, receiver gain; 0.1 s, time constant; 30 s for individual time, 5 scans were recorded per point during 50 min.

### Molecular docking simulations

Molecular docking simulations of ligands (alkenyl-S-ETE-PE and -PC) were performed in order to obtain its initial position at the catalytic site of 15-LOX. For each structure 5 runs were performed using the SMINA^96^ package to select further ligands with the lowest energy of binding for molecular dynamics simulations. The best alkenyl-S-ETE-PLs poses were bonded via the *sn*-2 chain in the catalytic site of 15-LOX-2 with a binding energy of −7.9 kcal/mol.

### Molecular dynamics (MD) simulations

Human 15-LOX-2 (isoform 2, PDB id: 4NRE)^97^ with a bound or unbound alkenyl-S-ETE-PE substrate, was simulated. Multiple MD runs were performed for 15-LOX-2 using the NAMD^98^ software with the CHARMM^99^ force field and 2 fs time steps. The protein was solvated with explicit water models TIP3P at physiological salt concentrations. The binding sites of alkenyl-S-ETE-PLs for substrate-bound simulations were predicted using the SMINA^96^ ligand-protein docking package. CHARMM force field parameters for covalently bonded iron were created based on the heme group and using the Gaussian^100^ package using B3LYP/6-31(d,p) basis set. The structure of alkenyl-S-ETE-PE was prepared in Maestro and parametrized using Swiss Param^101^. Prior to productive runs, the following protocol was adopted: 0.2 ns of water equilibration, 10,000 steps of minimization, 0.35 ns of heating from 0 to 300 K, and 0.15 ns equilibration of the whole system. Simulations were performed with a cutoff of 12 Å for non-bonded interactions and using the Langevin piston algorithm to maintain the temperature at 300 K and the pressure at 1 atm. We used VMD^102^ for visualization and ProDy^103^ for trajectory analysis with in-house scripts.

### Plasma membrane, endoplasmic reticulum, and mitochondria preparation

Sucrose density gradient ultracentrifugation was used for isolation of plasma membrane (PM) and endoplasmic reticulum (ER)^104^. Briefly, HBE cells (100 million) were incubated in hypotonic buffer (10 mM HEPES–KOH, pH 7.4, 1 mM EDTA) containing protease inhibitor cocktail on ice for 10 min to promote swelling. Sucrose was then added to a final concentration of 0.25 M, and cells were homogenized using a Dounce homogenizer followed by passage through an 18-gauge needle to ensure efficient disruption. The homogenate was subjected to differential centrifugation at 1000 × *g* for 10 min, 4000 × *g* for 10 min, and 12,000 × *g* for 15 min at 4 °C. The resulting supernatant after 12,000 × *g* was used for discontinuous sucrose density gradient ultracentrifugation, while the pellet (P2-fraction) was used for mitochondria preparation. Sucrose gradient consisted of 0.8 M and 1.2 M sucrose solutions in open-top thin-walled ultra-clear tubes (Beckman Coulter, Brea, CA). The supernatant was layered on top of 0.8 M sucrose solution and centrifuged at 100,000 × *g* for 150 min (SW-40 Ti rotor, 4 °C). Fractions corresponding to the 0.25/0.8 M and 0.8/1.2 M sucrose interfaces, as well as the bottom pellet, were collected, diluted in 10 mM HEPES (pH 7.4), and pelleted by ultracentrifugation at 100,000 × *g* for 60 min. The resulting pellets were resuspended in 10 mM HEPES and used for western blotting and lipidomics analysis. Mitochondrial fractions were prepared by washing the P2 fraction three times with 250 mM HEPES and centrifugation at 10,000 × *g* for 15 min. Finally, the pellet was resuspended in HEPES and used for lipidomics analysis. Enrichment of PM, ER, and mitochondria in different fractions was performed by western blotting using specific antibodies- E-cadherin for PM, calnexin for ER, and TOM20 for mitochondria (Supplementary Fig. 11).

### Cells

*Murine lung epithelial cell line* *(MLE)*, purchased from ATCC, was cultured at 37 °C and 5% CO_2_ in DMEM/F-12 (1:1) medium supplemented with 0.005 mg/ml insulin, 0.01 mg/ml transferrin, 30 nM sodium selenite (Life Technologies, Grand Island, NY), 10 nM hydrocortisone, 10 nM beta-estradiol (Sigma-Aldrich, Saint Louis, MO), 2 mM L-glutamine, 10 mM HEPES, and 10% FBS (Life Technologies, Grand Island, NY).

*HT-1080 cells* were obtained from ATCC and grown in DMEM with glutamine and sodium pyruvate (Corning 10-013) and supplemented with 10% heat-inactivated FBS, 1% non-essential amino acids (Invitrogen), and 1% penicillin-streptomycin mix (Invitrogen).

*Mouse embryonic fibroblasts (MEF)* were purchased from ATCC. Cell line was STR profiled at ATCC. Acsl4 KO cells were generated using CRISPR/Cas9 technology^58^. Cas9-expressing mouse embryonic fibroblasts were transfected with a plasmid expressing a gRNA targeting exon 1 of the Acsl4 gene.

### Bone marrow derived macrophages

Macrophages were isolated from bones of mice as described previously^105^. Briefly, the insides of bones were washed by placing a needle into the medullary cavity and injecting PBS to wash out the bone marrow. The obtained bone marrow cell suspension was homogenized by passing the sample up and down through an 18 G needle several times. Cells were then collected by centrifugation at 4 °C for 5 min and 1000 × *g*. Cells were resuspended in fresh DMEM supplemented with 20 ng/ml M-CSF, plated in Petri dishes, and cultured at 37 °C and 5% CO_2_.

*Primary human epidermal keratinocytes* (HEK, ATCC) were cultured using Dermal Cell Basal Medium (ATCC, PCS-200-030) with keratinocyte (ATCC, PCS-200-040) growth factor kits and Antibiotic Antimycotic solution (Sigma), and kept at passage 10 or below for all experiments. Cells were passaged weekly for cell culture maintenance using Trypsin-EDTA for Primary Cells (ATCC) and Trypsin Neutralizing Solution (ATCC) and supplemented with fresh media biweekly. For all assays, cells were used at 70% confluence.

### RNA isolation and quantitative RT-PCR

Total RNA was isolated from keratinocytes using TRIzol Reagent (Thermo Fisher) as per the manufacturer’s instructions and quantified using a DeNovix DS-11 spectrophotometer. Quantitative RT-PCR was performed using TaqMan Fast Advanced Master mix (Applied Biosystems) and beta-actin (Applied Biosystems). The primer/probe for target gene was FAM-ZEN/IBFQ labeled, and for actin (control) was VIC-MGB-PL labeled. Duplex reactions (target gene + actin) were run and analyzed on StepOnePlus (Applied Biosystems). Relative fold changes were calculated and normalized based on the 2^–ΔΔCt^ method.

### Primary human airway epithelial cell culture in air–liquid interface

HAECs were cultured in air–liquid interface (ALI) under serum-free condition as previously described^106,107^. Briefly, fresh bronchoscopic brushing primary HAECs or banked samples were cultured under immersed conditions for proliferation. When 80–90% confluent, cells were trypsinized and plated at 5 × 104 cells per well on 12-well Transwell plates for submerged stage culture by adding 200 μl culture medium to upper insert and 1000 μl culture medium to lower chamber. BEBM/DMEM at 50:50, supplemented with 4 g/ml Insulin, 5 pg/ml Transferrin, 0.5 µg/ml, Hydrocortisone, 0.5 µg/ml Epinephrine, 52 µg/ml Bovine hypothalamus extract, 50 µg/ml, Gentamicin, 50 ng/ml Amphotericin, 0.5 µg/ml albumin bovine, 80 nM ethanolamine, 0.3 mM, MgCl_2_, 0.4 mM MgSO_4_, 1 mM CaCl_2_, 30 ng/ml retinoic acid and 0.5 ng/ml Epithelial Growth Factor (EGF) was used as a cultured medium. When cells reached 100% confluence, they were then moved into ALI culture by reducing the upper volume to 50 µl with the lower volume remaining at 1.0 mL full medium with ETE supplement (10 µM). HAECs were cultured under ALI for 9 days and then stimulated with IL-13 (10 ng/ml) for the last 5 days before harvest for subsequent analysis. For RSL3 treatment, RSL3 (10 µM) and or ferrostatine-1 (Fer-1) (1 µM) was added overnight, with DMSO as a control.

### 15-LOX-1 knock down in HAEC cells

Dicer-substrate short interfering RNAs (DsiRNA) transfection was performed using Lipofectamine transfection reagent. The siALOX15 DsiRNA^TM^ (5′ - UGUUUUACGCUAAAGAUGGAAAAGA - 3′; 3′ - CAACAAAAUGCGAUUUCUACCUUUUCU - 5′, were purchased from Integrated DNA Technology (Coralville, IA). Briefly, 50 nM DsiRNA was pre-mixed with 3 μl/well Lipofectamine transfection reagent for 20 min at room temperature before being pooled together with HAECs suspension and seeded onto trans wells for incubation. After 24 hrs, the transfection mixture was removed, and cells were switched to ALI culture for 7 days. Cells were stimulated with IL-13 (10 ng/ml) under ALI culture for 7 days.

### PEBP1 knock down in HAEC cells

siRNA transfection of HAECs was performed using Lipofectamine RNAiMAX transfection reagent. The siPEBP1 DsiRNA^TM^; (5′ AGACCAGGUCUACAGUGA; 3′ 3′CAGAUGUCACUAUCUUGC 5′) was purchased from Integrated DNA Technology (Coralville, IA). Briefly, DsiRNA was pre-mixed with Lipofectamine transfection reagent (ThermoFisher Scientific) for 20 min at room temperature before being added to HAECs in suspension and seeded onto trans wells for incubations. After 24 h, the transfection mixture was removed, and cells were switched to ALI culture for 7 days.

### Isolation of mitochondrial fractions

Mitochondrial fractions were isolated using the Mitochondria Isolation Kit for Cultured Cells (ab110171, Abcam) with some modifications of the manufacturer’s instructions. Briefly, HAECs were collected from trans well membranes with a cell lifter and pelleted by centrifugation at 700 × *g* for 5 min. The cells were frozen and thawed to weaken cell membranes, then resuspended in Reagent A and incubated on ice for 10 min. This suspension was transferred into a pre-cooled Dounce Homogenizer, where it was homogenized for 3–5 strokes. Homogenates were centrifuged at 1000 × *g* for 10 min at 4 °C and saved as supernatant #1. The pellet was resuspended in Reagent B, and cell rupturing was repeated. These homogenates were centrifuged and saved as supernatant #2. Supernatants #1 and #2 were combined and centrifuged at 6000 × *g* for 15 min. The pellet was washed twice with PBS to remove contamination and centrifuged at 6000 × *g* for 15 min. The pellet was resuspended in Reagent C supplemented with Protease Inhibitors, and aliquots were frozen at −80 °C until use.

### Western blot

Total proteins were harvested in protein lysis buffer (50 mM Tris-HCl, 150 mM NaCl, 10 mM EDTA, 1.0 % NP-40). After being denatured at 95 °C in 5X SDS sample buffer for 5 min, the samples were run in 12% sodium dodecyl sulphate–polyacrylamide (SDS-PAGE) gels (Invitrogen) and transferred onto polyvinylidene difluoride (PVDF) membrane (Invitrogen). After the membrane was blocked with 5% skim milk, target proteins were immunodetected using primary antibodies. Membranes were developed using an Amersham Imager 600 (GE Healthcare Life Sciences) with SuperSignal West Femto Maximum Sensitivity Substrate (Thermo Fisher Scientific, 34096). Densitometry analysis was performed using Amersham Imager 600 and ImageJ software (NIH).

### Ferroptosis assay

Cells were seeded in 48-well plates in DMEM media. To inhibit spontaneous ferroptosis, media for GPX4^−/−^ A375 cells contained 175 nM Fer-1. WT cells were incubated in the presence of RSL3 (100 nM) with 15LOX-2 (100 nM) and DOPC liposomes containing either diacyl-S-ETE-PE or alkenyl-S-ETE-PE or diacyl-S-ETE-PC or alkenyl-S-ETE-PC. GPX4^−/−^ A375 cells were cultured in the presence of 2 μM Fer-1. GPX4^−/−^ cells were incubated in the presence of Fer-1 (175 nM) with 15-LOX-2 (100 nM) and DOPC liposomes containing either diacyl-S-ETE-PE or alkenyl-S-ETE-PE or diacyl-S-ETE-PC or alkenyl-S-ETE-PC. Ferroptotic inhibitor Fer-1 in concentration 1 μM was added to some samples to assess the percentage of cells dying from ferroptosis. Twenty hours after cell death was determined by measuring the release of lactate dehydrogenase (LDH) using the CytoToxONE™ Cyto-toxicity Detection Kit (Promega) according to the manufacturer’s instructions.

### LDH assays

Cell-free culture supernatants and cell lysates were collected and measured for LDH using the Lactate Dehydrogenase Assay Kit (Abnova, Walnut, CA). The results are presented as percent (%) released, determined as the following: % released = *n*(LDH in supernatants)/[*n*(LDH in supernatants) + *n*(LDH in cell lysis)] × 100%. *n* = LDH activity unit.

*Human melanoma A375 cells* were obtained from American Type Culture Collection (ATCC). Cells were grown in DMEM media with 10% FBS, 50 U/mL penicillin, 50 µg/mL streptomycin (all from Gibco), and 2.5 µg/mL plasmocin prophylactic (InvivoGen). GPX4^−/−^ A375 cells were obtained from Dr. Wei Gu (Columbia University, NY), cultured in the presence of 2 μM Fer-1.

### AGPS knock down in A375 Cells

A375 cells were transfected with control scrambled DsiRNA (51-01-14-04) (Integrated DNA Technology) or a mix of two Dicer-substrate siRNAs (DsiRNAs) against AGPS The siAGPS DsiRNA^TM^ (hs.Ri.AGPS.13.1: 5′ - GCAAGAAUUGUUUCACUAAAGAAAC - 3′, 3′ - AACGUUCUUAACAAAGUGAUUUCUUUG - 5′; hs.Ri.AGPS.13.2: 5′ - CAUGGGUUUACCAACAUUUAAAGAA - 3′, 3′ - CCG UAC CCA AAU GGU UGU AAA UUU CUU - 5′), were purchased from Integrated DNA Technology (Coralville, IA). Briefly, 8 μL of Lipofectamine RNAiMAX (Life Technology) was mixed with 40 pmol of siRNA in 400 μL Opti-MEM media (Thermo Fisher) and incubated at room temperature for 20 min. 6 × 10^5^ cells were seeded in a 6 cm dish in antibiotic-free DMEM (Thermo Fisher), and the siRNA-Lipofectamine mixture was added immediately. After 8 to 12 h, the transfection mixture was removed, and cells were maintained in antibiotic-free media for 36 h, after which cells were counted and seeded into a 48 well or 96 well plate for 24 h.

### TMEM189 knock down in A375 cells

A375 cells were transfected with control scrambled DsiRNA (51-01-14-04) (Integrated DNA Technology) or a mix of two Dicer-substrate siRNAs (DsiRNAs) against TMEM189 (hs.Ri.TMEM189.13.1 and hs.Ri.TMEM189.13.2) as described above. The siTMEM189 DsiRNA^TM^ (hs.Ri.TMEM189.13.1: 5′ - ACCUGAGACAGUGAAUUAAUGUUGA - 3′, 3′ - AGUGGACUCUGUCACUUAAUUACAACU - 5′; hs.Ri.TMEM189.13.2: 5′ - GAGCUGAUGUUUCCAAUACCAAGAT - 3′, 3′ - GUCUCGACUACAAAGGUUAUGGUUCUA - 5′) were purchased from Integrated DNA Technology (Coralville, IA).

### TMEM164 knockdown in A375 cells

A375 cells were transfected with control scrambled DsiRNA (51-01-14-04) (Integrated DNA Technology) or a mix of two Dicer-substrate siRNAs (DsiRNAs) against TMEM164 (hs.Ri.TMEM164.13.1 and hs.Ri.TMEM164.13.2) as described above. The siTMEM164 DsiRNA^TM^ (hs.Ri.TMEM164.13.1: 5′ - CCCUUUGAAUUGGAGAUUUACUACA - 3′, 3′ - AGGGGAAACUUAACCUCUAAAUGAUGU - 5′; hs.Ri.TMEM164.13.2: 5′ - CAUUGGUAAUUCAGUGUCCUAUGAA - 3′, 3′ - UAGUAACCAUUAAGUCACAGGAUACUU -5′) were purchased from Integrated DNA Technology (Coralville, IA). Cell death induction and western blot sample collection were also conducted as described above. For lipidomic analysis, 10^6^ cells were seeded in 10-cm dishes 48 hours after knockdown. Cells were cultured in these dishes for 24 hours, after which lipids were extracted and analyzed as described above.

### Treatment of A375 cells with RSL3

Wild type A375 cells, AGPS KD, and TMEM189 KD cells were seeded in 48- or 96-well plates for 24 h (with fatty acid when applicable) and treated with the indicated concentrations of RSL3 and Fer-1 for 16 h. When applicable, oleic acid (10 µM) or ETE (10 µM), conjugated to fatty-acid free BSA at a 3:1 molar ratio, were added to media at the time of seeding. After 24 h, for cell death experiments, the fatty acids were removed, and cells were treated with RSL3 at different concentrations in the absence and in the presence of ferrostatin-1 (Fer-1, 1 μM). Untreated cells were collected for western blotting at the same point (96 h post-transfection). Cell death and relative viability were measured with the CytoToxONE™ Cyto-toxicity Detection Kit (Promega) or the CellTiter-Glo 2.0 Cell Viability Assay (Promega).

### Western blot

Total proteins were harvested in protein lysis buffer (50 mM Tris-HCl, 150 mM NaCl, 10 mM EDTA, 1.0% NP-40). After being denatured at 95 °C in 5X SDS sample buffer for 5 min, the samples were run in 8–16% sodium dodecyl sulphate–polyacrylamide (SDS-PAGE) gels (Invitrogen) and transferred onto polyvinylidene difluoride (PVDF) membrane (Invitrogen). After the membrane was blocked with 5% skim milk in TBST, target proteins were immunodetected using primary antibodies diluted in 1% BSA in TBST. Membranes were developed using an Amersham Imager 600 (GE Healthcare Life Sciences) with SuperSignal West Femto Maximum Sensitivity Substrate (Thermo Fisher Scientific, 34096). Densitometry analysis was performed using Amersham Imager 600 and ImageJ software (NIH).

### Ferroptosis assay and Sytox Green staining

GPX4⁻^/^⁻ A375 cells were incubated in DMEM without fetal bovine serum, supplemented with 175 nM ferrostatin-1 (Fer-1), and either diacyl-S-HpETE-PE or alkenyl-S-HpETE-PE- for 1 h, and then FBS was added. To determine the contribution of ferroptosis to cell death, some samples were treated with Fer-1 at a final concentration of 1 μM. After 24 h of incubation, 2 μM Sytox Green was added to stain dead cells. Fluorescence was measured using a Cytation 5 imaging reader (excitation/emission: 500/530 nm). To quantify total Sytox Green fluorescence, cells were lysed with 0.1% Triton X-100.

### Synthesis of alkenyl-S-15-HpETE-PE

Incubation conditions: 1.3 mM alkenyl-S-ETE-PE was dried under N_2_ and then solubilized in 0.2 mM pH 7.8 HEPES buffer containing deoxycholic acid (10 mM) in the presence of DTPA (100 µM). The mixture was bubbled with 95% O_2_ and incubated with recombinant human 15-LOX-2 (10 µM) at 37 °C (under continuous vortexing) for 90 min in the dark. The reaction was stopped by the extraction of lipid by Folch procedure^108^. Deoxycholic acid was removed by 1D HPTLC (silica, 5 µm, 5 × 5 cm, Whatman) using solvent system: chloroform/methanol/ammonium hydroxide (65:25:5, v/v/v). Purity of alkenyl-S-15HpETE-PE was confirmed by LC-ESI-MS/MS using a C30 reverse phase column and a QExactive Orbitrap mass spectrometer as described below (Supplementary Fig. 12). Purified PE-OOH (99%) was reconstituted in DMSO and added to A375 melanoma cells.

*Mouse hippocampal neuronal cell line HT22* (Sigma, SCC129) was maintained in Dulbecco’s modified Eagle’s medium (DMEM, Gibco 11995073) with 10 % FBS, 1X penicillin/streptomycin, and incubated at 37 °C, 5% CO_2_, and 95% humidity.

### 15-LOX knock down in HT22 cells

Mouse Hippocampal Neuronal HT22 cells (1.0 × 10⁵ cells/well) were seeded in 6-well plates to reach 60% confluence the next day. Cells were transfected in Opti-MEM (Gibco, 31985062) with either ON-TARGET plus silencer siRNA targeting mouse Alox15 (15-LOX) or ON-TARGET plus non-targeting siRNA (Dharmacon) at a final concentration of 100 nM and a lipid transfection reagent (DharmaFECT 1), prepared per manufacturer’s instructions. The siALOX15 ON-TARGETplus siRNA (L-061509-01; siRNA-1: 5′-GGAAAUUGAGAUUCGUAAC-3′; siRNA-2: 5′-GGCAAGUCAUGAAUCGGUA-3′; siRNA-3: 5′-GGGACAAUGGACACCGUUA-3′; siRNA-4: 5′-UGGCUGAGCGGGUUCGAAA-3′) were purchased from Horizon Discovery (Dharmacon, Lafayette, CO, USA). A non-targeting control siRNA (D-001810-04-05) was used as a negative control. After 24 h, transfection mix was replaced with complete DMEM. After counting cells were seeded in BioFlex (BF-3001L) plate for in vitro TBI or 6 well plate for western blot. Ninety-six hours after transfection, cells were washed with PBS, and cell lysates were prepared using lysing buffer RIPA and protease inhibitor. Samples were vortexed (3 × 10 s), protein amount was estimated by BCA assay (ThermoFisher), diluted in Laemmli buffer (4x) and then loaded on 4-20 % Tris-glycine gradient gels (BioRad). After transfer to PVDF membrane and blocking (1 hr) with 5 % milk in TBST, protein expression was detected using anti-15-LOX-2 (1:400) antibody (sc-271290) following overnight incubation at 4 °C. The membrane was washed with TBST (3 times), incubated with an HRP-conjugated goat anti-mouse antibody (1:1000, 1 h), and then developed with SuperSignal West Pico Chemiluminescent Substrate (ThermoFisher) using Biorad Chemidoc Imaging System. The membrane was then stripped for 15 min, blocked with 5% milk, and reprobed with β-actin as loading control.

### Mechanical stretch model of traumatic brain injury in vitro

Sventy two hours after transfection, cells were re-seeded at 6 × 10⁴ cells/well onto laminin-coated, silicone-based deformable membrane BIOFLEX® culture plates (3001 L, Flexcell International) and incubated for 24 h to allow adherence. At 96 h, mechanical stretch injury was induced using the Cell Injury Controller II (CIC II, Custom Design & Fabrication). All experiments used identical parameters (regulator pressure 52.0 psi, peak pressure 7.8 psi, 99 ms duration), producing a single rapid biaxial deformation of the membrane and associated stretch-injury to cultured cells. Experimental conditions included: control, stretch, stretch + Ferrostatin-1 (Fer-1; 1 µM, 1 h pre-treatment), and the corresponding untreated, non-targeting siRNA and 15-LOX siRNA KD groups. Following stretch injury, cells were transferred to a 96-well plate 16 h later for a cell viability assay (CellTiter-Glo) and were subsequently prepared for MALDI-MS imaging.

### Localization of 15-LOX-2 in mouse brain

Tissue slices (10 μm) were fixed in 4% paraformaldehyde (PFA) in PBS for 15 min at room temperature, followed by three washes with PBS. Samples were permeabilized by incubation in 0.1% Triton X-100 in PBS for 10 min. Following a 1 h blocking with 20% normal goat serum and 1% bovine serum albumin (BSA) in PBS, samples were incubated overnight at 4 °C with primary antibodies diluted in the blocking solution with anti-15-LOX-2 (sc-271290, Santa-Cruz, USA, 1:500) and anti-NeuN Cy3-conjugated (MAB377, Millipore Sigma, USA, 1:400). On the subsequent day, samples were rinsed thrice with PBS and incubated with suitable secondary antibodies conjugated to Alexa Fluor 647 (1:200) for 1 h at room temperature in the dark. Nuclei were counterstained with DAPI at a concentration of 1 µg/mL for 5 min. Following the final washes with PBS, coverslips were affixed using antifade mounting media. Images were obtained from pericontusional area with a confocal fluorescence microscope (Zeiss LSM 900, Germany)

*Human bronchial epithelial (HBE) cells* (a HBE cell line originally established by Dieter Gruenert)^109^ were cultured in MEM (Thermo Fisher Scientific) supplemented with 10% FCS (Gibco), 50U/ml penicillin-streptomycin (Thermo Fisher Scientific), plasmocin (InvivoGen), and l-glutamine (Thermo Fisher Scientific). HBE cells were used in experiments until passage 50.

### Inhibition of ATP-dependent aminophospholipid translocase (APT) and labeling of PE with fluorescamine in HBE cells

To inhibit aminophospholipid translocase (APT), HBE cells were treated with 50 mM 2-deoxyglucose in the presence of 50 mM sodium azide in glucose-free medium for 30 min at 37 °C. This treatment resulted in a reduction of intracellular ATP levels by more than 90% and did not compromise cell viability. HBE cells (2 × 10⁶) were suspended in Hanks’ Balanced Salt Solution (HBSS) and gently agitated with 200 μM fluorescamine for 30 s. At the end of incubation, 40 mM Tris-HCl, pH 7.4, was added to bind free fluorescamine. After that lipids were extracted and analyses of fluorescamine labeled diacyl-S-ETE-PE (*m*/*z* 1044.5971), diacyl-O-ETE-PE (*m*/*z* 1042.5815), diacyl-P-ETE-PE (*m*/*z* 1016.5658), diacyl-PO-ETE-PE (*m*/*z* 1014.5502), and alkenyl-S-ETE-PE with (*m*/*z* 1028.6022), alkenyl-O-ETE-PE (*m*/*z* 1026.5866), alkenyl-P-ETE-PE (*m*/*z* 1000.5709), and alkenyl-PO-ETE-PE (*m*/*z* 998.5552) species were performed using LC-MS/MS. Fluorescamine labeled diacyl-S-ETE-PE and alkenyl-S-ETE-PE species were synthesized in our lab (with purity of 99%) and used as references to build standard calibration curves (Supplementary Fig. 13).

### Animal models

#### Mouse tumor model

Animal experiments were approved by the Wistar Institute Animal Care and Use Committee as well as the University of Pennsylvania Institutional Animal Care and Use Committee and by the Institutional Animal Care and Use Committee of AstraZeneca (Gaithersburg, MD) and conducted in Association for Assessment and Accreditation of Laboratory Animal Care (AAALAC)–accredited and United States Department of Agriculture (USDA)–licensed facility and the AstraZeneca Global Bioethics policy. *Alox12/15fl* (age 8–10 weeks) were obtained from Jackson Laboratory and crossed with B6.Cg-Tg (S100 A8-cre, -EGFP) 1Ilw/J (Jackson Laboratory). Female mice were used due to practical reasons in this exploratory study with small number of biological replicates. Sex based analysis was not performed. All mice were housed in autoclaved cages with access to food and water ad libitum in a sterile environment maintained with a 12 h dark/12 h light cycle at 72 ± 2 °F with 50 ± 20% room humidity. In mouse tumor models, the maximal tumor size approved by the IACUC was 2 cm in the largest diameter. In none of the experiments were exceeded these limits. LLC (Lewis lung carcinoma) cells were obtained from ATCC. Cells were maintained in DMEM medium supplemented with 10% fetal bovine serum (FBS, Sigma-Aldrich) and penicillin–streptomycin at 37 °C under 5% CO_2_ and were tested for mycoplasma contamination using the Universal Mycoplasma Detection Kit (ATCC). Tumor cells were injected subcutaneously at 5 × 10^5^ cells per mouse. Tumor tissues were dissociated using a tumor dissociation kit (Miltenyi) and then sorted by FACSAria (BD Biosciences) to obtain PMN-MDSCs as the CD11b^+^Ly6G^+^Ly6C^-^ population.

#### Isolation of PMNs from Alox12/15^fl^ mice

Single-cell suspensions were prepared from bone marrow using ammonium chloride lysis buffer. PMN-MDSCs were purified using anti-Ly6G microbeads (Miltenyi Biotec) according to the manufacturer’s instructions or sorted using the FACS Aria (CD45^ + ^CD11b^ + ^Ly6G^+^Ly6Clow) system.

#### PMN viability assay

PMN-MDSCs were treated with RSL3 (10 µM) for 4 h, washed 3 times, and incubated for another 16 h. Cell viability was evaluated using the trypan blue exclusion method.

#### Expression of Alox12/15 in PMNs by qRT-PCR with reverse transcription

RNA was extracted using the Total RNA Kit according to the manufacturer’s instructions. DNase digestion was performed; cDNA was generated using the High-Capacity cDNA Reverse Transcription Kit (Applied Biosystems). Quantitative PCR (qPCR) was performed using the Power SYBR Green PCR Master Mix (Applied Biosystems) with primers (forward: GGCTCCAACAACGAGGTCTA and reverse: TGAATTCTGCCTCCGAGTTCC) in 96-well plates. The plates were read using an ABI 7900 (Applied Biosystems) system.

#### Controlled cortical impact mouse model

All procedures were performed according to the protocols established by the Institutional Animal Care and Use Committee of the University of Pittsburgh (protocol # 21069524) and Columbia University Institutional Animal Care and Use Committee (IACUC) under approval number ABV3664. Male C57BL6J mice (Jackson Laboratories, Bar Harbor, ME), 12–15 weeks of age and weighing 27 ± 1.8 g, were housed under controlled environmental conditions and allowed ad libitum food and water during the study. Mice were group-housed and kept in 12-h light/dark cycles with temperature and humidity controlled. CCI model was used as previously described^110^. Briefly, 8 to 12-week-old wild-type C57BL/6 were anesthetized with 4.5% isoflurane (Anaquest) in 70% nitrous oxide and 30% oxygen using a Fluotec 3 vaporizer (Colonial Medical). The mice were placed in a stereotaxic frame, and a 5-mm craniotomy was made over the parieto-temporal cortex using a drill and a trephine. The bone flap was removed and discarded, and a pneumatic/electromagnetic cylinder with a 3-mm flat tip impounder with velocity 6 m/s, depth 1.2 mm, and duration 100 ms was used to induce CCI. The scalp was sutured closed, and the mice were returned to their cages to recover. Mice were sacrificed 4 hrs after CCI, and ipsilateral and cortical areas of cortex were isolated and used to estimate the levels of 15-LOX-2 and for lipidomics. In experiments with baicalein, vehicle (cremophor El and ethanol solution and diluted with normal saline (CEE:NS, 1:3, v/v)) or baicalein (50 mg/kg) was administered via intraperitoneal injection at 10–15 min post-CCI^39^. All studies were performed in a blinded manner.

### Human samples

#### Normal human skin samples

Tissue samples of the normal human skin were collected from three white males, ages 67–75, after obtaining signed informed consent. The study of the collected tissue was approved by the Institutional Review Board of the University of Pittsburgh (PRO15100580). To obtain epidermal sheets (human epidermal explants, HEE), full thickness skin explants were cut and incubated in 1 U/mL dispase in DMEM/F-12 (StemCell Technologies) for 7 h at 37 °C. Intact epidermal sheets were separated from the dermis, and treated with either vehicle (DMSO), Fer-1 (10 μM), or z-VAD-fmk (20 μM) for 12–16 h. HEE were exposed to UVB (5 kJ/m^2^). All studies were performed in a blinded manner.

#### Sources of HAEC for in vitro and ex vivo studies

This research complies with all relevant ethical regulations and study protocol that was approved by University of Pittsburgh Institutional Review Board (IRB). All participants provided informed consent and were compensated for their time and effort. All studies were performed in a blinded manner. The characteristics of these participants are included in two separate demographics tables depending on the study (Supplementary Tables 1 and 2). HAECs were obtained by bronchoscopic brushing of asthmatic and healthy control (HC) airways according to the RBl protocol. All participants were recruited as part of the Immune-epithelial Cell Interactions in Severe Asthma (P01 AI106684 and P01AI106684-06A1)^107^. All asthmatic participants met American Thoracic Society (ATS) criteria for asthma and included mild to severe asthmatic patients, while HCs were without respiratory disease and had normal lung function^110,111^. No participant smoked within the last year or for >5 pack years or was studied within 4 weeks of an asthma exacerbation. All participants were extensively evaluated through lung function testing (race-neutral FEV_1_ equations), FeNO, allergy testing, and questionnaires. Exacerbation history was determined by questionnaires. Severe asthma was defined by ERS/ATS criteria, including use of high-dose inhaled and/or systemic corticosteroids, in combination with a 2nd controller to maintain control or who remained uncontrolled^110^. Mild asthma participants were those who did not meet severe asthma criteria.

#### Phospholipidomics

Total lipids from liposomes, cells, and tissues were extracted using the Folch procedure^108^, and phosphorus was determined by a micro-method^112^. Briefly, cells (1.5 × 10^6^) were resuspended in 0.9% KCl, pieces of tissues (2–10 mg of protein) were homogenized in PBS containing DTPA (100 μM), and lipids were extracted using chloroform methanol mixture of 2:1 (v/v). To prevent oxidation of lipids during extraction and sample preparation for LC/MS analysis, a chloroform-methanol mixture containing 0.01% butylated hydroxytoluene was used. LC/ESI-MS analyses of phosphatidylethanolamines (PEs) and phosphatidylcholines (PCs) and their hydroperoxy and hydroxy species were performed on a Thermo HPLC system coupled to a Thermo Scientific™ Orbitrap Fusion™ Lumos™ Tribrid™ mass spectrometer. Lipids were separated on a normal phase column (Luna 3 μm Silica (2) 100 A, 150 × 1.0 mm, (Phenomenex)) at a flow rate of 0.065 ml/min. The injection volume was 5 µL. The column was maintained at 35 °C. The analysis was performed using gradient solvents: Solvent A was 285:215:5 2-propanol:hexanes:water, and solvent B was 285:215:40 2-propanol:hexanes:water with 10 m M ammonium formate for both. The samples were analyzed using the following gradient conditions: 0 min: 15% B, 3–15 min: 37% B, 23–75 min: 100% B, 76–90 min: 15% B^52^. Lipids were analyzed in negative ion mode using data dependent analysis with the following parameters: capillary voltage, 3500; sheath, aux, and sweep gases (35, 17, 0, respectively); ion transfer tube temperature, 300 °C. MS1 analysis used the orbitrap as the detector (120,000 resolution) with the following parameters: scan range 400-1800 *m*/*z*; Rf lens, 40; injection time, 100 ms; AGC target 50%. Data dependent MS^2^ analysis was performed with the following parameters: threshold 4e^2^; quadrupole isolation 1.2 *m*/*z*; injection time 22 ms; HCD stepped normalized collision energy 15,30,45; orbitrap detection (15,000 resolution). Compound Discoverer^TM^ software package (ThermoFisher Scientific) with an in-house generated analysis workflow and oxidized phospholipid database was used to evaluate LC/MS data. Peaks with a signal/noise ratio of >3 were identified and searched against the database of oxidized phospholipids. Lipid signals were further filtered by retention time, and values for *m/z* were matched within 5 ppm to identify the lipid species. Under conditions used, the number of points required per peak was from 10 to 15. Commercially available 1-stearoyl-2-15(S)-HpETE-*sn*-glycero-3-phosphoethanolamine, 1-stearoyl-2-15(S)-HETE-sn-glycero-3-phosphoethanolamine, 1-stearoyl-2-15(S)-HpETE-sn-glycero-3-phosphocholine, 1-stearoyl-2-15(S)-HETE-*sn*-glycero-3-phosphocholine (Cayman Chemicals) were used as reference standards for oxidized phospholipids. 1-stearoyl-2-ETE-*sn*-glycero-3-phosphoethanolamine, 1-stearoyl-2-ETE-*sn*-glycero-3-phosphocholine, 1-(1Z-octadecenyl)-2-arachidonoyl-sn-glycero-3-phosphoethanolamine, 1-(1Z-octadecenyl)-2-arachidonoyl-*sn*-glycero-3-phosphocholine (Avanti Polar Lipids) were used as reference standards to build the calibration curve for non-oxidized phospholipids. Deuterated phospholipids: 1-hexadecanoyl(d31)-2-(9Z-octadecenoyl)-*sn*-glycero-3-phosphoethanolamine, 1-hexadecanoyl(d31)-2-(9Z-octadecenoyl)-*sn*-glycero-3-phosphocholine, (Avanti Polar Lipids) were used as internal standards. Internal standards were added directly to the MS sample to a final concentration of 1 µM. All samples were analysed randomly.

### Matrix-assisted laser desorption ionization mass spectrometry imaging (MALDI-MSI)

#### Sample preparation

After injury, cells intended for imaging were transferred to IntelliSlides with growth chambers (Millicell® EZ Slide) and incubated for 16 h to allow attachment. Chambers were then removed, and slides were gently washed to reduce salt (2 × 10 s in PBS, followed by 1 × 10 s in 50 mM ammonium acetate), wicked from the edge, and dried under a gentle stream of nitrogen at room temperature. No chemical fixation was used. Matrix: 1,5-diaminonaphthalene (1,5-DAN) was prepared at 10 mg/mL in 70% acetonitrile (freshly prepared, vortexed, sonicated for 15 min, and filtered through a 0.22 µm membrane) and applied using a pneumatic TM-Sprayer™ (M3 + , HTX Technologies). Spraying parameters were: 10 passes, nozzle temperature 75 °C, flow rate 0.10 mL/min, track speed 1300 mm/min, pressure 10 psi, pattern: crisscross (CC), with 2.5 mm track spacing. Red phosphorus was used for external calibration of the mass spectrometer.

#### MALDI timsTOF MSI acquisition

Imaging was performed on a Bruker timsTOF fleX MALDI-2 (Smartbeam 3D laser) in negative ion mode with a spatial resolution of 5 µm. Laser settings were: 30 shots per pixel, 10 kHz repetition rate, and 32% laser power (instrument a.u., adjusted to avoid matrix overburn). The acquisition mass range was *m*/*z* 650–1050 for timsOFF and *m*/*z* 650–1350 for timsON, with ion transfer optimized for lipids (Funnel RF 350 Vpp; Multipole RF 300 Vpp; Transfer Time 110 µs; Pre-Pulse Storage 15 µs; Collision RF 1600 Vpp; Collision Energy 10 eV; Ion Energy 10 eV; high-sensitivity detection mode).

For timsON datasets, ion mobility separation used an accumulation time of 31 ms, ramp time 470 ms, and a 1/K₀ scan range of 1.25–1.50 V s cm⁻², with standard CCS calibration (vendor defaults).

#### Data processing and analysis

Ion images were generated in SCiLS Lab Version 2025a Pro (Bruker) using a ±15 ppm mass window around theoretical *m*/*z* values and a ±2 Å tolerance for CCS values in timsON mode, with normalization to total ion current (TIC). Mass accuracy was maintained at <10 ppm. For single-cell quantification, segmentation maps were generated using the automated feature-finding tool in SCiLS Lab across the *m*/*z* 650–1350 lipid range to extract cellular boundaries. The number of lipid-positive pixels per cell corresponding to PE(38:4) + 1(O), PEp(38:4) + 1(O), PE(38:5) + 1(O), and PEp(38:5) + 1(O) was assessed within segmented cell regions (*n* = 20 cells per condition) (Supplementary Figs. 14 and 15**)**. CCS values were determined from timsON datasets and cross-validated with MS/MS imaging datasets.

#### Statistical analysis

Statistical analyses were performed by either an unpaired *t*-test, one-way ANOVA with Tukey’s multiple comparisons test, or two-way ANOVA with Tukey’s multiple comparisons test, using GraphPad Prism 10.3.1 software. The data are presented as means ± SD values. The exact value of sample size (*n*) is presented in figure legends and reflects either the number of experimental replications with cells or biochemical model systems or the number of animals. For measurements on human samples, *n* represents the number of patients. Statistical analyses were performed by either two-tailed paired *t*-test or one-way ANOVA for normally distributed data. When the overall ANOVA revealed a significant effect, the data were further analyzed with the Tukey/Dunn post-hoc test to determine specific group differences. The statistical significance of differences was set at *p* < 0.05. Phospholipids were quantified from the full scan LC/MS spectra with ratio-metric comparison to the pre-selected internal standard using corresponding standard curves. Differences between the groups were analyzed by either two-sided unpaired *t*-test or one-way ANOVA with Tukey post-hoc analyses. For ANOVAs *F*-values and degree of freedom are provided. For the *t*-test, *t*-values and degree of freedoms are provided. Power analysis uses assumptions about the expected results of the research based on previous studies, along with certain statistical assumptions, to obtain the sample sizes required for detecting as statistically significant the expected results.

### Reporting summary

Further information on research design is available in the Nature Portfolio Reporting Summary linked to this article.

## Supplementary information

Supplementary Information

Description of Additional Supplementary Files

Supplementary Movie 1.

Supplementary Movie 2.

Supplementary Movie 3.

Supplementary Movie 4.

Supplementary Movie 5.

Supplementary Movie 6.

Reporting Summary

Transparent Peer Review file

## Source data

Source Data

## Data Availability

Data generated during the study and included in this article are available from the corresponding authors upon request. Source data are provided with this paper.
