# Supplementary material for: 15-LOX-catalytic bias towards ether-(alkenyl)-ETE-PEs oxidation bestows selectivity of PRO-ferroptotic cell death signaling
Source: Nat Commun. 2026 Jun 17;17:5360. doi: 10.1038/s41467-026-71869-z (PMC13276253; doi:10.1038/s41467-026-71869-z)
Supplement: Supplementary file 1 — Supplementary Information [file 41467_2026_71869_MOESM1_ESM.pdf]

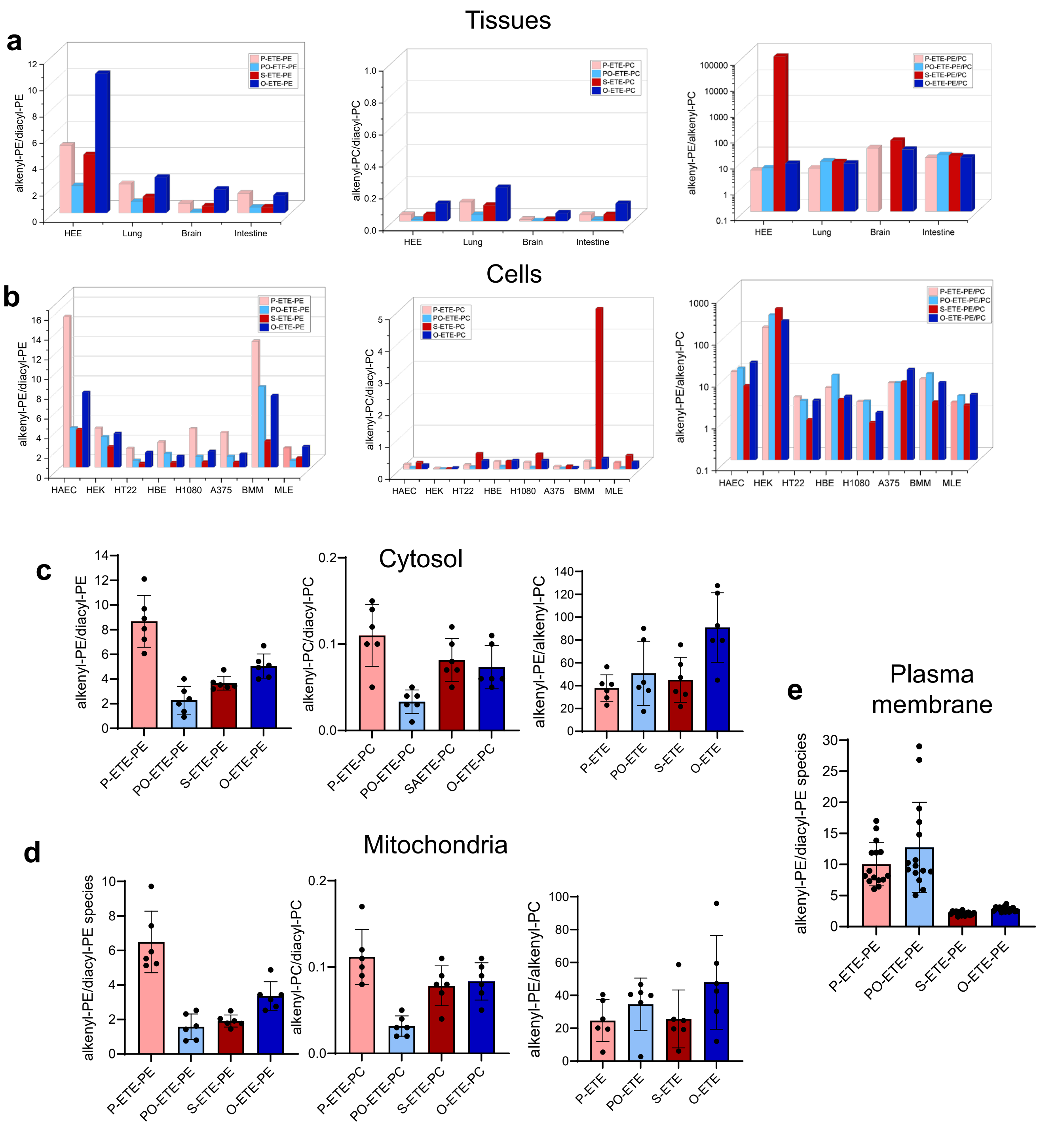

Supplementary Fig. 1.

Supplementary Fig. 1. Contents of diacyl- and alkenyl-ETE-PE and ETE-PC species in tissues, cells and cell organelles.

a Ratios of diacyl- to alkenyl-ETE-PE (left panel), diacyl- to alkenyl-ETE-PC species (middle panel) and alkenyl-PE to alkenyl-PC (right panel) in tissues. HEE- human epidermal explants. **b** Ratios of diacyl- to alkenyl-ETE-PE (left panel), diacyl- to alkenyl-ETE-PC species (middle panel) and alkenyl-PE to alkenyl-PC (right panel) in cells. HAEC – Human Airway Epithelial Cells; HEK – Human Epidermal Keratinocytes; HT22 – Mouse Hippocampal Neuronal cells; HBE – Human Bronchial Epithelial Cells; HT1080 – Human epithelial cells derived from connective tissue from a patient with Fibrosarcoma; A375 – Human Melanoma cells; BMM – Bone Marrow Macrophages (mouse); MLE – Mouse Lung Epithelial Cells. Ratios of diacyl- and alkenyl-ETE-PE (left panel), diacyl- and alkenyl-ETE-PC species (middle panels) and alkenyl-PE to alkenyl-PC (right panels) in cytosol (**c**) and mitochondria (**d**) isolated from HAEC. Mean ± SD, n=5 (five HAEC cultures, each from different donors). **e** Ratios of alkenyl- to diacyl-ETE-PE species in plasma membrane of HAEC. Mean ± SD, three HAEC cultures each from a different donor were used, n=5-6 biological replicates for each HAEC culture.

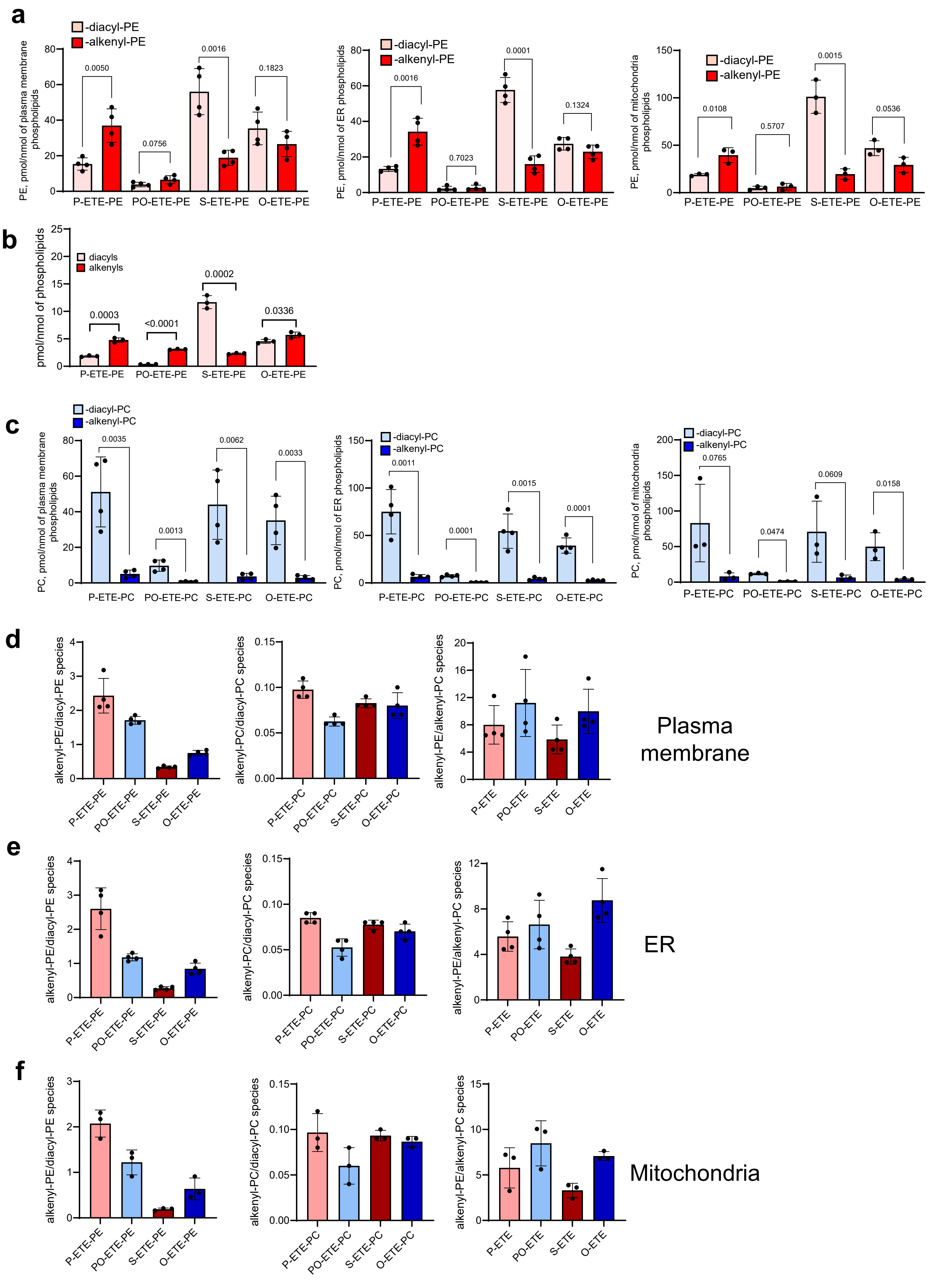

Supplementary Fig. 2.

Supplementary Fig. 2. Content of ETE-PE species in cellular organelles of HBE cells.

a Content of diacyl- and alkenyl-PE species in plasma membrane (left panel), ER (middle panel) and mitochondria (right panel) isolated from HBE cells. Data are normalized to total phospholipids in each of the respective fractions. Mean ± SD, n=4 biological samples for plasma membrane; n=3 for mitochondria; n=4 for ER, unpaired two-sided Student's t-test. Plasma membrane: Diacyls vs alkenyls - P-ETE-PE (t=4.318, p=0.0050, df=6), PO-ETE-PE (t=2.145, p=0.0756, df=6), S-ETE-PE (t=5.466, p=0.0016, df=6), O-ETE-PE (t=1.508, p=0.1823, df=6); ER: Diacyls vs alkenyls - P-ETE-PE (t=5.452, p=0.0016, df=6), PO-ETE-PE (t=0.4010, p=0.7023, df=6), S-ETE-PE (t=9.872, p<0.0001, df=6), O-ETE-PE (t=1.741, p=0.1324, df=6); Mitochondria: Diacyls vs alkenyls - P-ETE-PE (t=4.501, p=0.0108, df=4), PO-ETE-PE (t=0.6169, p=0.5707, df=4), S-ETE-PE (t=7.699, p=0.0015, df=4), O-ETE-PE (t=2.708, p=0.0536, df=4); **b** Content of diacyl-ETE-PE and alkenyl-ETE-PE assessed by fluorescamine labelling in plasma membrane of HBE cells. Mean ± SD, n=3 independent biological samples. Unpaired two-sided Student's t-test revealed significant differences between plasma membrane diacyl-P-ETE-PE vs. alkenyl-P-ETE-PE (t=11.5, p=0.0003, df=4), diacyl-PO-ETE-PE vs. alkenyl-PO-ETE-PE (t=62.93, p<0.0001, df=4), diacyl-S-ETE-PE vs. alkenyl-S-ETE-PE (t=13.55, p=0.0002, df=4), diacyl-O-ETE-PE vs. alkenyl-O-ETE-PE (t=3.178, p=0.0336, df=4). **c** Content of diacyl- and alkenyl- PC species in plasma membrane (left panel), ER (middle panel) and mitochondria (right panel) isolated from HBE cells by differential centrifugation. Data are normalized to total phospholipids in each of the respective fractions and presented as Mean ± SD, n=4 biological samples for plasma membrane; n=3 for mitochondria; n=4 for ER, unpaired two-sided Student's t-test. Plasma membrane: Diacyls vs alkenyls - P-ETE-PC (t=4.655, p=0.0035, df=6), PO-ETE-PC (t=5.699, p=0.0013, df=6), S-ETE-PC (t=4.124, p=0.0062, df=6), O-ETE-PC (t=4.700, p=0.0033, df=6). ER: Diacyls vs alkenyls - P-ETE-PC (t=5.840, p=0.0011, df=6), PO-ETE-PC (t=11.43, p<0.0001, df=6), S-ETE-PC (t=5.531, p=0.0015, df=6), O-ETE-PC (t=9.085, p<0.0001, df=6). Mitochondria: Diacyls vs alkenyls - P-ETE-PC (t=2.374, p=0.0765, df=4), PO-ETE-PC (t=2.829, p=0.0474, df=4), S-ETE-PC (t=2.587, p=0.0609, df=4), O-ETE-PC (t=4.025, p=0.0158, df=4). **d** Ratios of diacyl- and alkenyl-ETE-PE (left panels), diacyl- and alkenyl-ETE-PC species (middle panels) and alkenyl-PE to alkenyl-PC (right panels) in plasma membrane (**d**), ER (**e**) and mitochondria (**f**) isolated from HBE cells. Mean ± SD, n=4 biological samples.

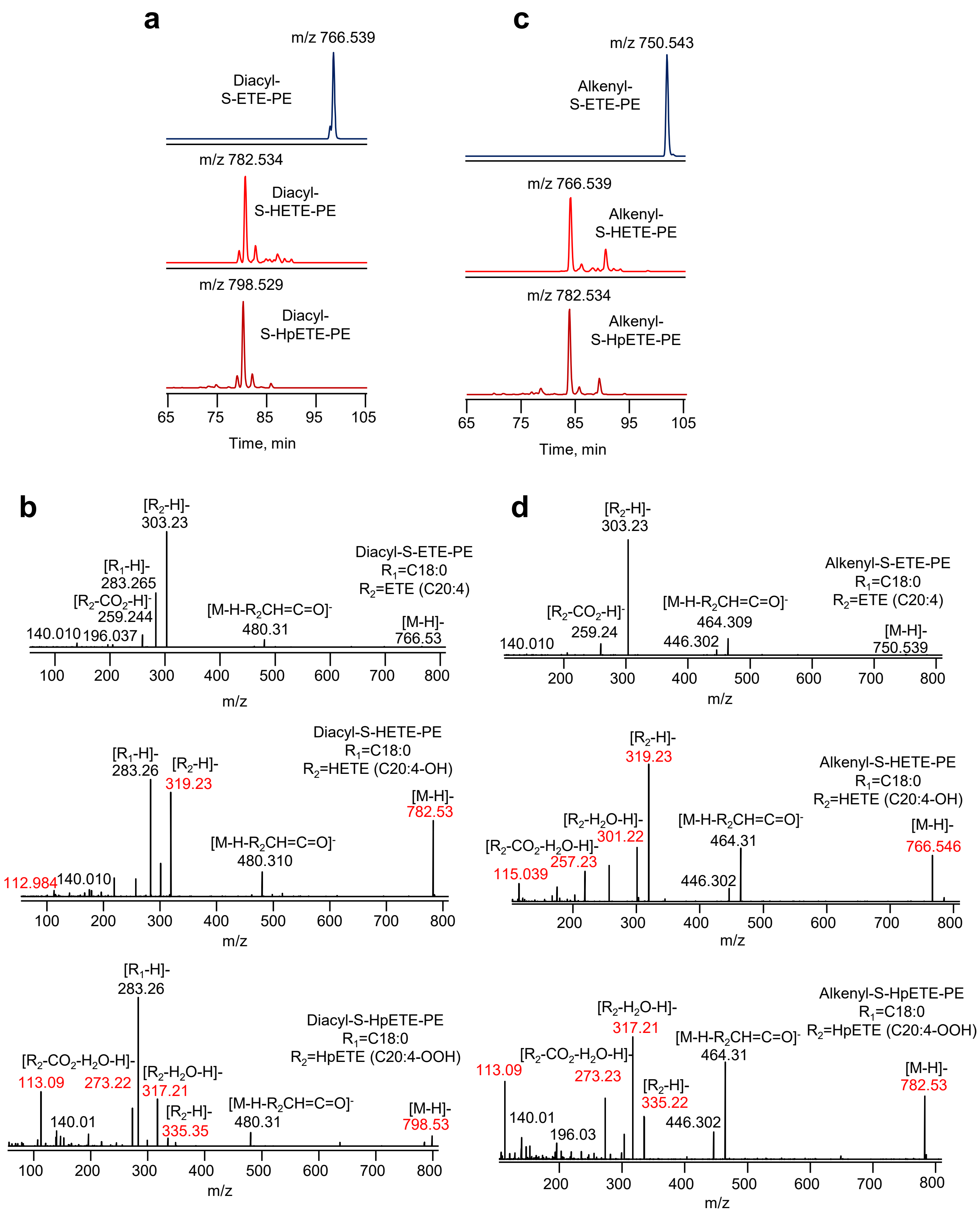

Supplementary Fig. 3.

Supplementary Fig. 3. Structural analysis of diacyl-S-ETE-PE and alkenyl-S-ETE-PE and their oxidation products formed in 15-LOX-1 driven reaction in liposomes.

a LC/MS base profiles of diacyl-S-ETE-PE (m/z 766.539, RT=98.6 min, upper panel) and its oxidation products, diacyl-S-HETE-PE (m/z 782.534, RT=80.6 min, middle panel) and diacyl-S-HpETE-PE (m/z 798.529, RT=80.1 min, lower panel). m/z -mass to charge ratio; RT - retention time. **b** MS^2 fragmentation patterns of diacyl-S-ETE-PE (upper panel) and its oxidation products, diacyl-S-HETE-PE (middle panel) and diacyl-S-HpETE-PE (lower panel). Upper panel: The fragments with m/z 283.26 and m/z 303.23 correspond to stearic acid (C18:0) and eicosatetraenoic acid (ETE, C20:4) localized in the *sn*-1 and *sn*-2 positions of diacyl-S-ETE-PE assigned as $[R_1\text{-H}]^-$ and $[R_2\text{-H}]^-$, respectively. Fragment with m/z 259.24 derived from ETE after loss of CO_2 assigned as $[R_2\text{-}CO_2\text{-H}]^-$. Middle panel: The fragments m/z 283.26 and with m/z 319.23 correspond to stearic acid (C18:0) and HETE (C20:4-OH) localized in the *sn*-1 and *sn*-2 positions of diacyl-S-ETE-PE assigned as $[R_1\text{-H}]^-$ and $[R_2\text{-H}]^-$, respectively. Lower panel: The fragment m/z 283.26 corresponds to stearic acid (C18:0) in the *sn*-1 of diacyl-S-HpETE-PE assigned as $[R_1\text{-H}]^-$. The fragments with m/z 335.35, m/z 317.21 and 273.22 assigned as $[R_2\text{-H}]^-$, $[R_2\text{-}H_2O\text{-H}]$- and $[R_2\text{-}CO_2\text{-}H_2O\text{-H}]$- correspond consequently to HpETE (C20:4-OOH), HpETE after loss of water and HpETE without both water and CO_2. On all MS^2 spectra, fragments with m/z 480.31 assigned as $[M\text{-H-}R_2CH\text{=C=O}]^-$ were generated with loss of ETE as ketene. The fragments with m/z 140.01 and 196.03 are attributed to polar head of PE species. The fragments with m/z 113.09 are used for the identification of 15-HpETE. **c** LC/MS base profiles of alkenyl-S-ETE-PE (m/z 750.543, RT=102.1 min, upper panel) and its oxidation products, alkenyl-S-HETE-PE (m/z 766.539, RT=84.3 min, middle panel) and alkenyl-S-HpETE-PE (m/z 782.534, RT=83.7 min, lower panel). **d** MS^2 fragmentation patterns of alkenyl-S-ETE-PE (upper panel) and its oxidation products, alkenyl-S-HETE-PE (middle panel) and alkenyl-S-HpETE-PE (lower panel). Upper panel: The fragment with m/z 303.23 corresponds to eicosatetraenoic acid (ETE, C20:4) localized in the *sn*-2 positions of alkenyl-S-ETE-PE assigned as $[R_2\text{-H}]^-$. Fragment with m/z 259.24 derived from ETE after loss of CO_2 assigned as $[R_2\text{-}CO_2\text{-H}]^-$. Middle panel: The fragments with m/z 319.23, m/z 301.22 and 257.23 assigned as $[R_2\text{-H}]^-$, $[R_2\text{-}H_2O\text{-H}]$- and $[R_2\text{-}CO_2\text{-}H_2O\text{-H}]$- correspond consequently to HETE (C20:4-OH), HETE after loss of water and HETE without both water and CO_2. Lower panel: The fragments with m/z 335.22, m/z 317.21 and 273.23 assigned as $[R_2\text{-H}]^-$, $[R_2\text{-}H_2O\text{-H}]$- and $[R_2\text{-}CO_2\text{-}H_2O\text{-H}]$- correspond consequently to HpETE (C20:4-OOH), HpETE after loss of water and HpETE without both water and CO_2. On all MS^2 spectra, fragments with m/z 480.31 assigned as $[M\text{-H-}R_2CH\text{=C=O}]^-$ were generated with loss of ETE as ketene. The fragments with m/z 140.01 and 196.03 are attributed to polar head of PE species. The fragments with m/z 113.09 are used for the identification of 15-HpETE.

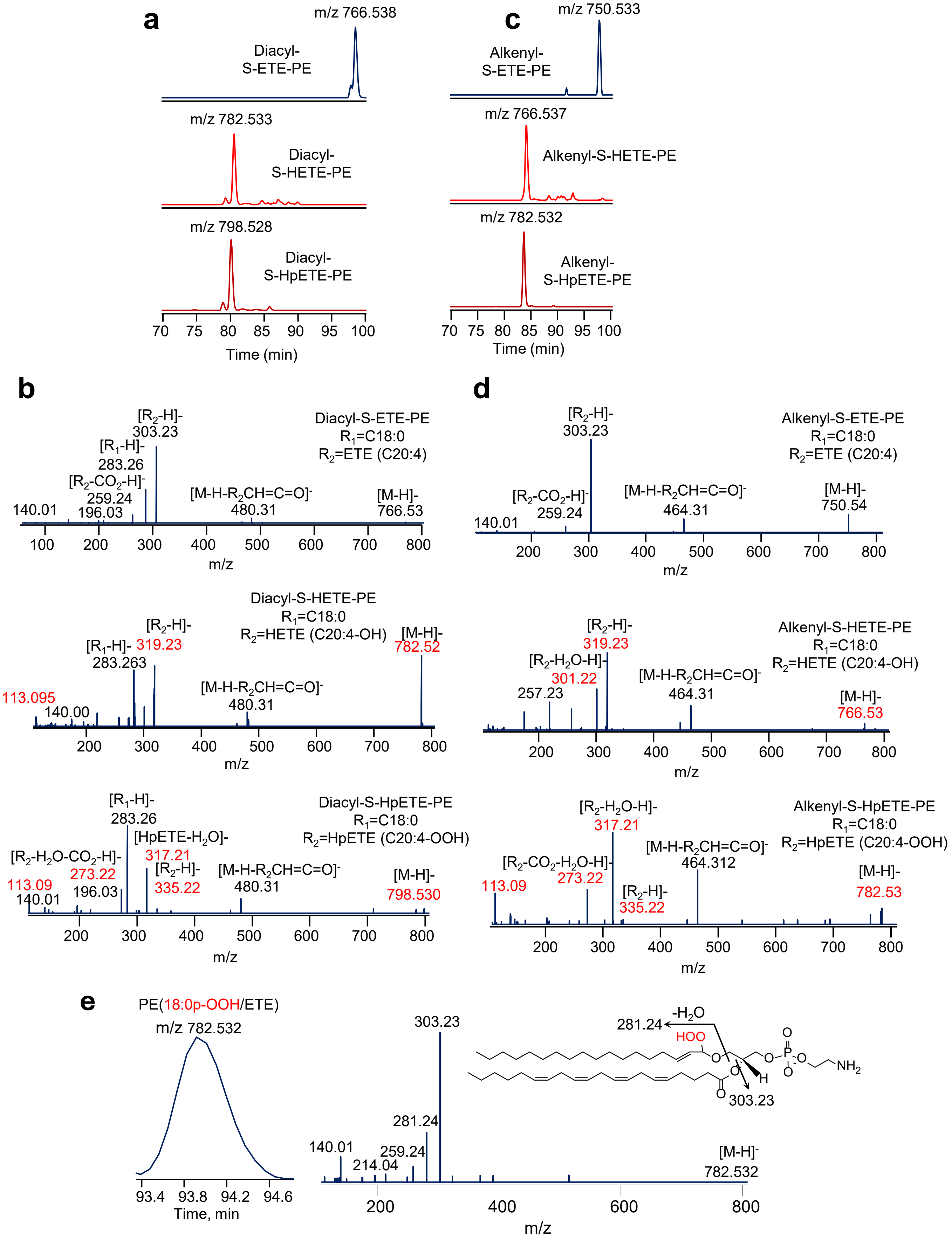

Supplementary Fig. 4.

Supplementary Fig. 4. Structural analysis of diacyl-ETE-PE and alkenyl-ETE-PE formed in 15-LOX-2 driven reaction in liposomes.

a LC/MS base profiles of diacyl-S-ETE-PE (m/z 766.538 RT=98.6 min, upper panel) and its oxidation products, diacyl-S-HETE-PE (m/z 782.533, RT=80.6 min, middle panel) and diacyl-S-HpETE-PE (m/z 798.528, RT=80.1 min, lower panel). m/z -mass to charge ratio; RT - retention time. **b** MS^2 fragmentation patterns of diacyl-S-ETE-PE (upper panel) and its oxidation products, diacyl-S-HETE-PE (middle panel) and diacyl-S-HpETE-PE (lower panel). Upper panel: The fragments with m/z 283.26 and m/z 303.23 correspond to stearic acid (C18:0) and eicosatetraenoic acid (ETE, C20:4) localized in the *sn*-1 and *sn*-2 positions of diacyl-S-ETE-PE assigned as $[R_1\text{-H}]^-$ and $[R_2\text{-H}]^-$, respectively. Fragment with m/z 259.24 derived from ETE after loss of CO_2 assigned as $[R_2\text{-}CO_2\text{-H}]^-$. Middle panel: The fragments m/z 283.26 and with m/z 319.23 correspond to stearic acid (C18:0) and HETE (C20:4-OH) localized in the *sn*-1 and *sn*-2 positions of diacyl-S-ETE-PE assigned as $[R_1\text{-H}]^-$ and $[R_2\text{-H}]^-$, respectively. Lower panel: The fragment m/z 283.26 corresponds to stearic acid (C18:0) in the *sn*-1 of diacyl-S-HpETE-PE assigned as $[R_1\text{-H}]^-$. The fragments with m/z 335.22, m/z 317.21 and 273.22 assigned as $[R_2\text{-H}]^-$, $[R_2\text{-}H_2O\text{-H}]$- and $[R_2\text{-}CO_2\text{-}H_2O\text{-H}]$- correspond consequently to HpETE (C20:4-OOH), HpETE after loss of water and HpETE without both water and CO_2. On all MS^2 spectra, fragments with m/z 480.31 assigned as $[M\text{-H-}R_2CH{=}C{=}O]^-$ were generated with loss of ETE as ketene. The fragments with m/z 140.01 and 196.03 are attributed to polar head of PE species. The fragments with m/z 113.09 are used for the identification of 15-HpETE. **c** LC/MS base profiles of alkenyl-S-ETE-PE (m/z 750.533, RT=102.1 min, upper panel) and its oxidation products, alkenyl-S-HETE-PE (m/z 766.537, RT=84.3 min, middle panel) and alkenyl-S-HpETE-PE (m/z 782.532, RT=83.7 min, lower panel). **d** MS^2 fragmentation patterns of alkenyl-S-ETE-PE (upper panel) and its oxidation products, alkenyl-S-HETE-PE (middle panel) and alkenyl-S-HpETE-PE (lower panel). Upper panel: The fragment with m/z 303.23 corresponds to eicosatetraenoic acid (ETE, C20:4) localized in the *sn*-2 positions of alkenyl-S-ETE-PE assigned as $[R_2\text{-H}]^-$. Fragment with m/z 259.24 derived from ETE after loss of CO_2 assigned as $[R_2\text{-}CO_2\text{-H}]^-$. Middle panel: The fragments with m/z 319.23, m/z 301.22 and 273.22 assigned as $[R_2\text{-H}]^-$, $[R_2\text{-}H_2O\text{-H}]$- and $[R_2\text{-}CO_2\text{-}H_2O\text{-H}]$- correspond consequently to HETE (C20:4-OH), HETE after loss of water and HETE without both water and CO_2. Lower panel: The fragments with m/z 335.22, m/z 317.21 and 273.22 assigned as $[R_2\text{-H}]^-$, $[R_2\text{-}H_2O\text{-H}]$- and $[R_2\text{-}CO_2\text{-}H_2O\text{-H}]$- correspond consequently to HpETE (C20:4-OOH), HpETE after loss of water and HpETE without both water and CO_2. On all MS^2 spectra, fragments with m/z 480.31 assigned as $[M\text{-H-}R_2CH{=}C{=}O]^-$ were generated with loss of ETE as ketene. The fragments with m/z 140.01 and 196.03 are attributed to polar head of PE species. The fragments with m/z 113.09 are used for the identification of 15-HpETE. **e** Typical LC-ESI-MS profile (left panel) and MS/MS spectrum (right panel) of oxygenated PE (18:0p/20:4) containing hydroperoxyacetal (PE (18:0p-OOH/20:4)) at m/z 782.532 and Rf=93.8 min, after incubation with 15-LOX-2. Possible structure is inserted.

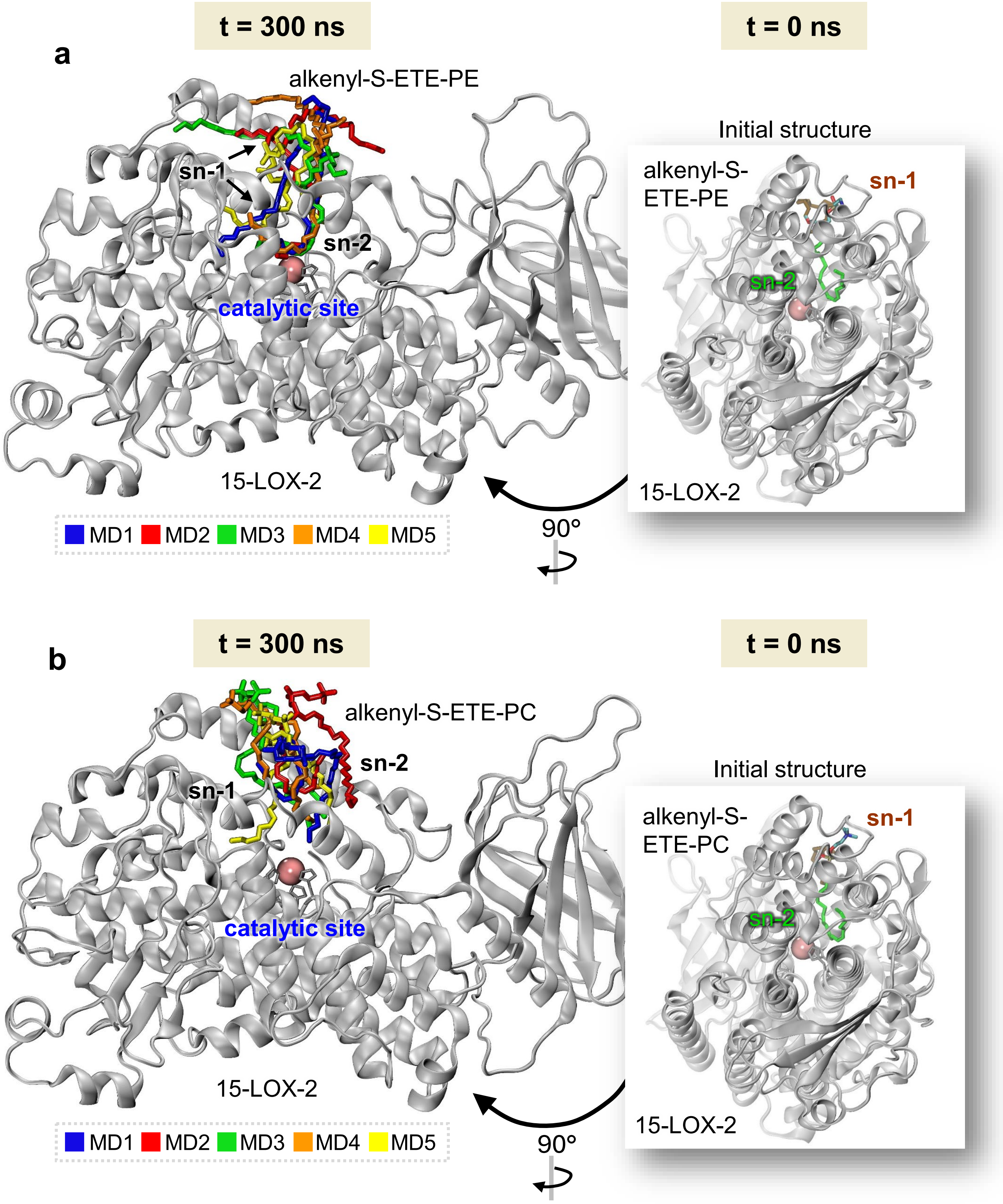

Supplementary Fig. 5. Substrate binding sites on 15-LOX-2 identified in MD simulations.

Final conformations of alkenyl-S-ETE-PE (**a**) and alkenyl-S-ETE-PC (**b**) molecules after 300 ns MD simulations (MD1-MD5) are shown in different colors (blue, red, green, orange, and yellow). The initial conformations of the alkenyl-S-ETE-PE (**a**) and alkenyl-S-ETE-PC (**b**) are shown in the insets where non-oxidizable *sn*-1 is colored in brown and oxidizable *sn*-2 in green.

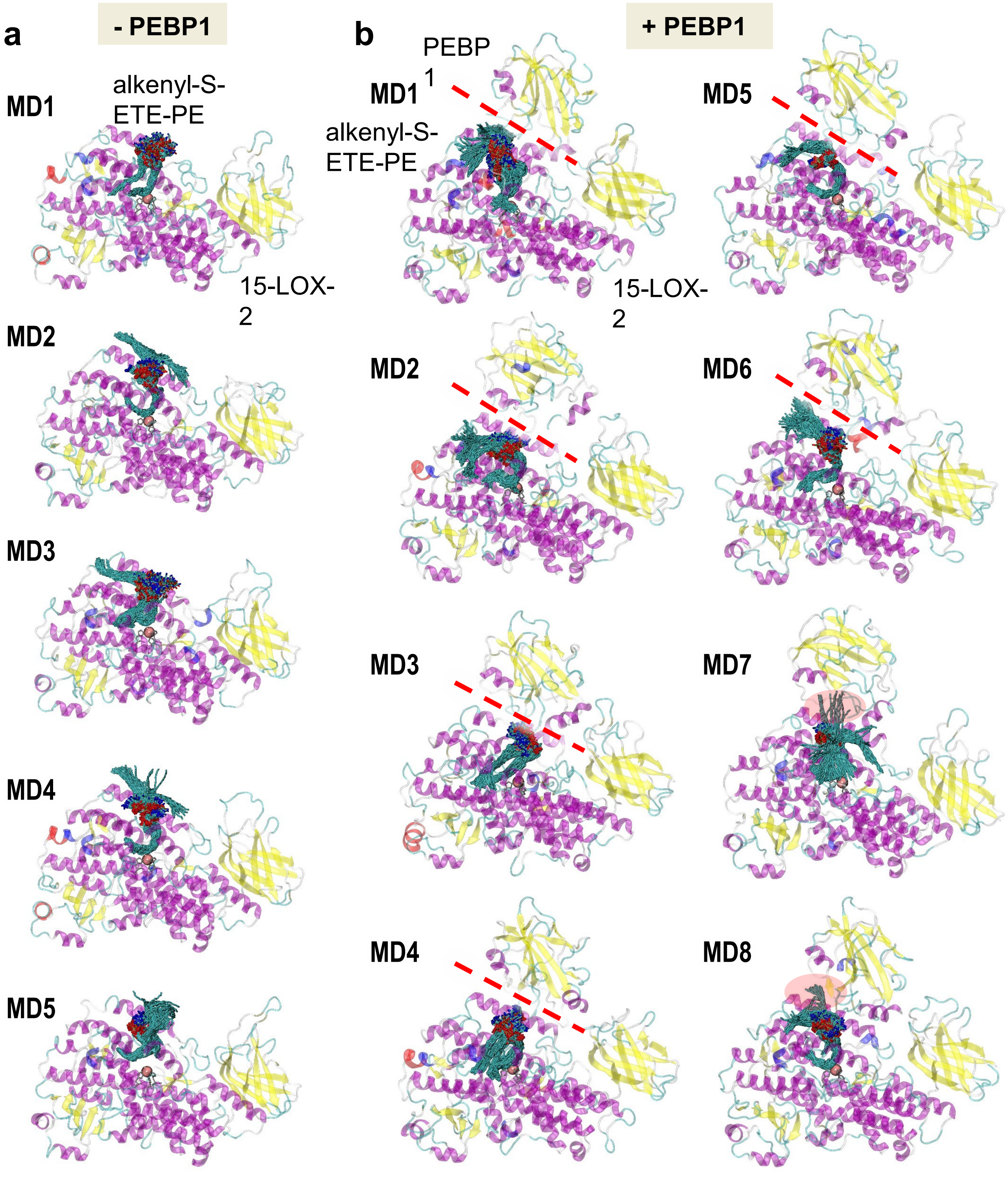

Supplementary Fig. 6. Interactions of alkenyl-PE with 15-LOX-2 observed in multiple MD runs conducted (a) in the absence and (b) in the presence of PEBP1 bound to 15-LOX-2.

Multiple conformations of alkenyl-PEs observed during 300 ns independent MD runs (MD1-MD5 in **a** and MD1-MD8 in **b**) are displayed. The oxidizable *sn*-2 tail of alkenyl-PE is shown in *green sticks.* The *pink sphere* and surrounding *grey sticks* represents the iron and the coordinating 15-LOX-2 catalytic residues (H378, H553, H373, I676). *Red dashed lines* denote a lack of interactions between PEBP1 and alkenyl-PE while the *light red ovals* highlight the interactions of the non-oxidizable *sn*-1 tail of alkenyl-PE with the α2 helix close to PEBP1 regardless of the presence of PEBP1.

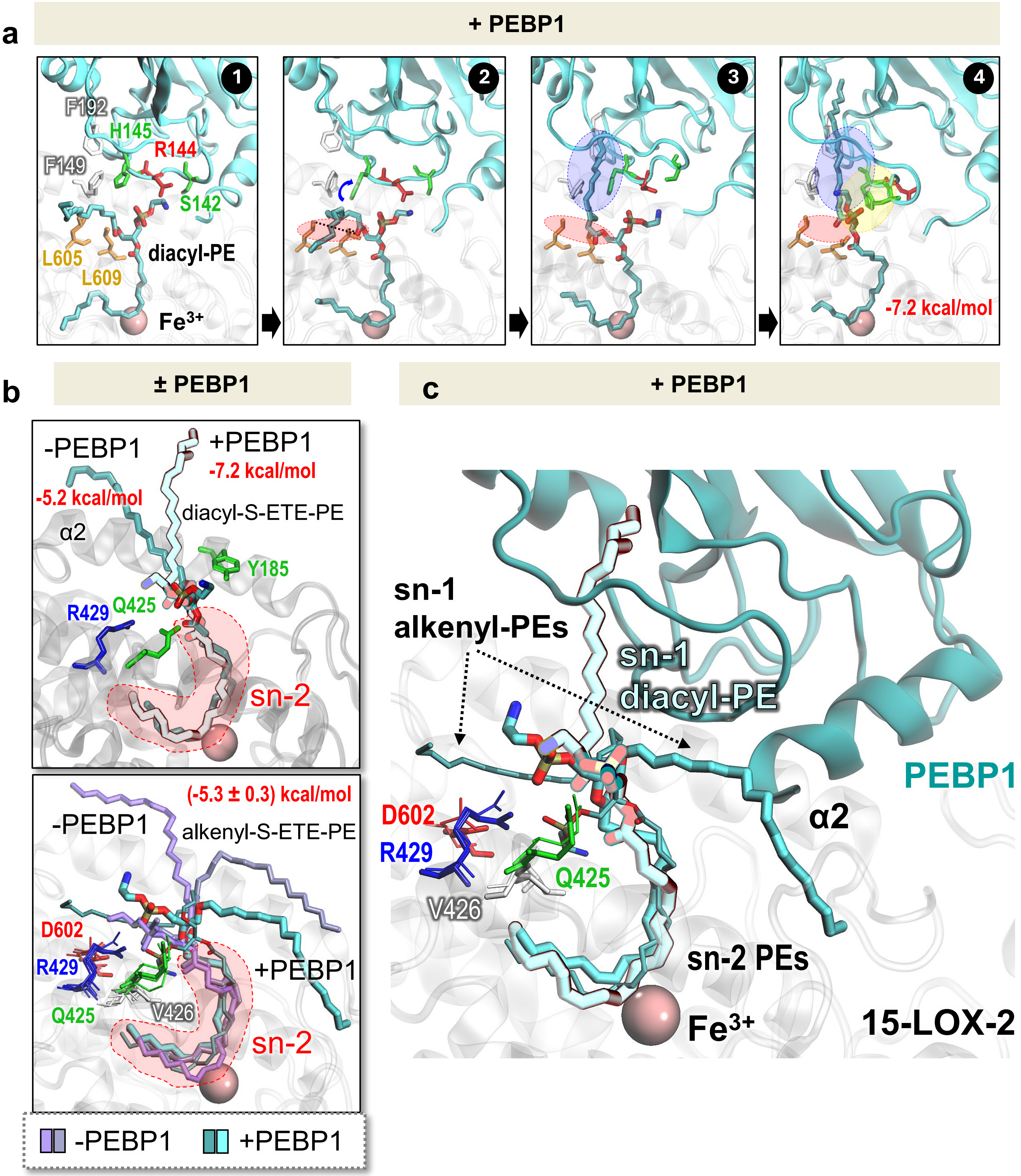

Supplementary Fig. 7.

Supplementary Fig. 7. Alkenyl-S-ETE-PE and diacyl-S-ETE-PE binding conformations on 15-LOX-2 shown for the PEBP1-bound and -unbound form of 15-LOX-2 identified in MD simulations.

a A sequence of MD snapshots illustrating the importance of the ester group at the *sn*-1 position of diacyl-S-ETE-PE in maintaning the interactions with L605 and L609 (#2, red oval) which helps stabilize the interactions between *sn*-1 and F149 (on 15-LOX-2) and F192 and H145 (on PEBP1) and promotes the further insertion into the PEBP1 structure (#3, blue oval). These interactions allow for the stabilization of the PE headgroup by H145 and S142 (#4, *yellow oval*), resulting in a high affinity binding (-7.2 kcal/mol). **b** Comparison of the bound conformations of diacyl-S-ETE-PE (*top*) and alkenyl-S-ETE-PE (*bottom*) in the presence and absence of PEBP1. Displayed residues (R429, Q425, and less frequently D602 and V426) contribute to stabilizing the PE headgroup of the alkenyl-PEs irrespective of PEBP1 presence, but for diacyl-S-ETE-PE, these interactions are observed only in the absence of PEBP1 as shown more clearly in the closeup view (panel **c**). The binding affinity of the alkenyl-S-ETE-PEs in the presence/absence of PEBP1 for displayed conformations is (-5.3 ±0.3) kcal/mol.

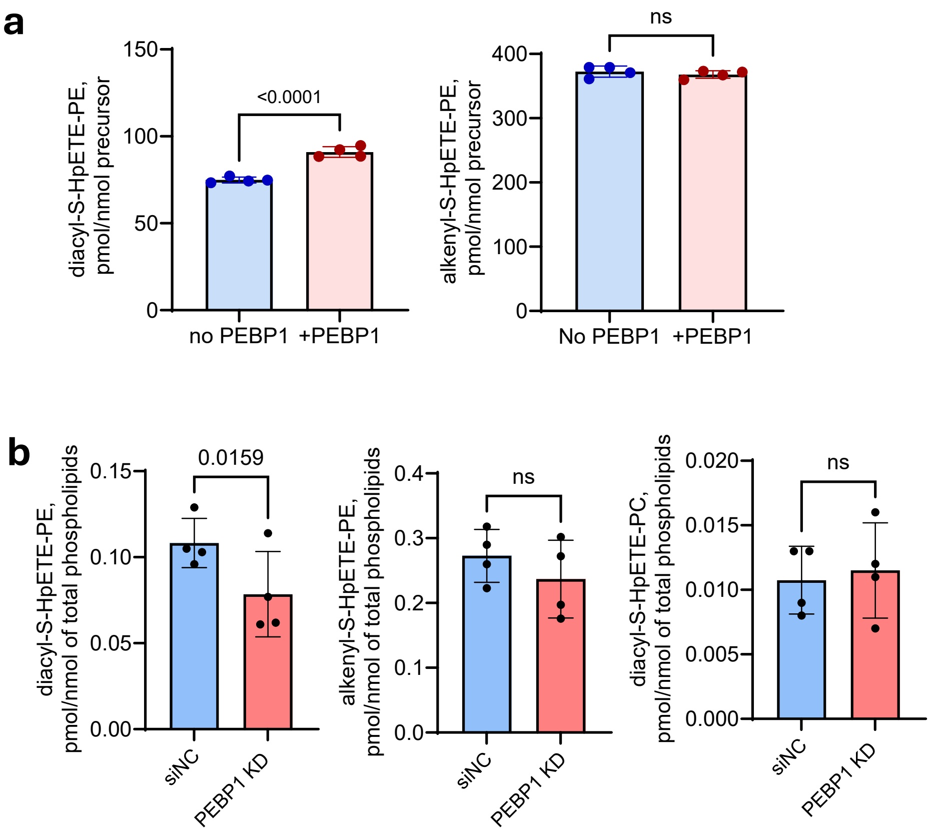

Supplementary Fig. 8. Effect of PEBP1 on 15-LOX induced oxidation of ETE-PE in model system and PEBP1 KD HAECs.

a Effect of PEBP1 on 15-LOX-2 catalyzed oxidation of diacyl-S-ETE-PE (left) and alkenyl-S-ETE-PE (right) in model system. Mean ± SD, n=4 experimental samples, unpaired two-sided Student's t-test for diacyl-S-HpETE-PE, no PEBP1 vs. +PEBP1 (t=9.201, p<0.0001, df=6). **b** Effect of PEBP1 on the content of diacyl-S-HpETE-PE (left), alkenyl-S-HpETE-PE (middle) and diacyl-S-HpETE-PC species (right) in HAEC cells. Mean ± SD, n=4 (each from a different donor), unpaired two-sided Student's t-test. Diacyl-S-HpETE-PE: siNC vs PEBP1 KD (t=4.943, p=0.0159, df=3), ns - not significant.

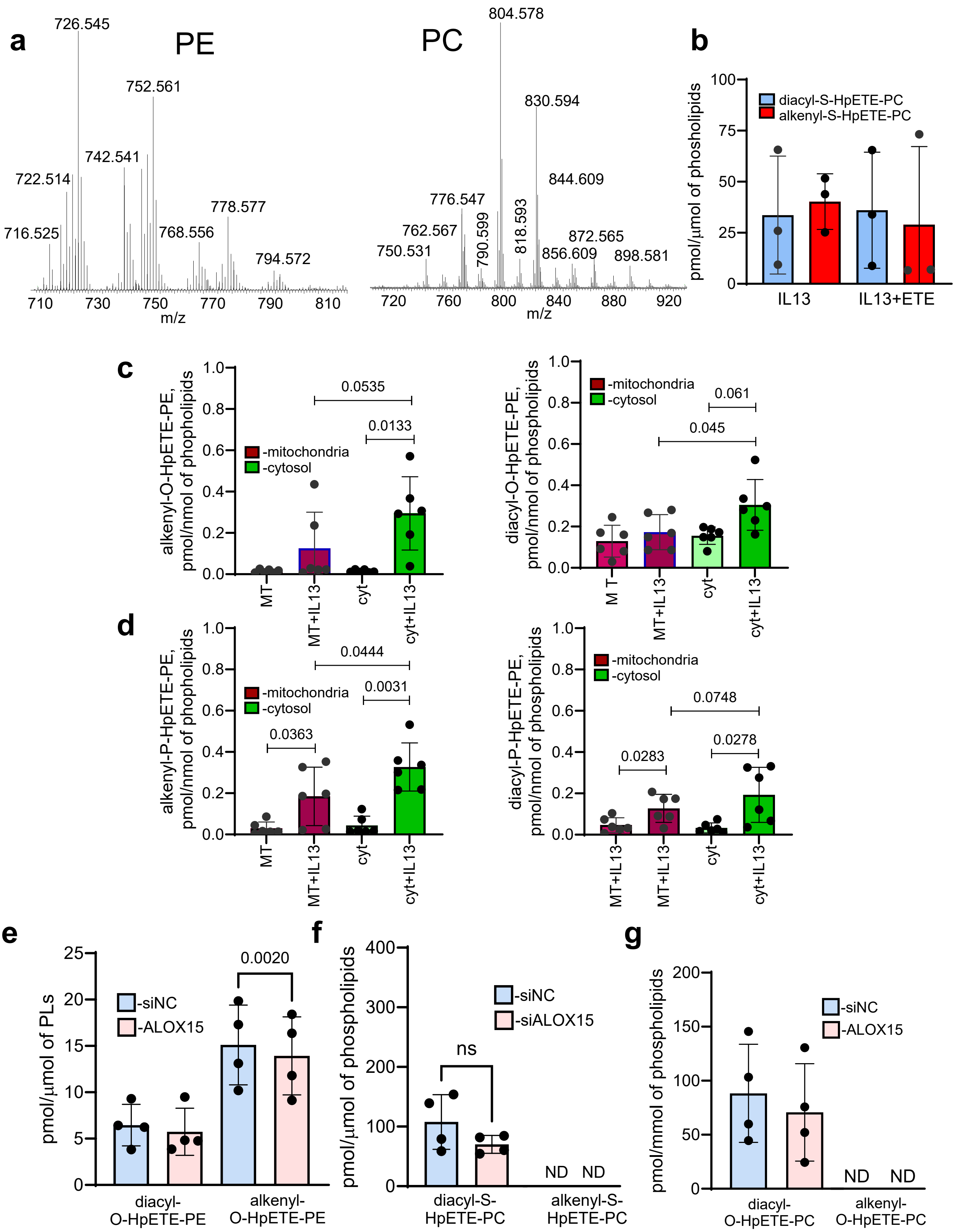

Supplementary Fig. 9.

Supplementary Fig. 9. Oxidation of alkenyl- and diacyl-ETE-PE species induced by 15-LOX-1 in HAECs.

a Typical mass spectra of PE (left panel) and PC (right panel) obtained from HAECs. **b** Content of diacyl-S-HpETE-PC in HAECs exposed to IL13. Alkenyl-S-HpETE-PC were not detected. Mean ± SD, n = 4 biological replicates (four HAEC cultures each from a different donor). Paired two-sided Student's t-test revealed no significant differences. **c** Accumulation of alkenyl-O-HpETE-PE (left panel, cytosol: cyt vs cyt+IL13, unpaired two-sided Student's t-test, t=3.750, p=0.0133, df=5) and diacyl-O-HpETE-PE (right panel, MT+IL13 vs cyt+IL13, unpaired two-sided Student's t-test, t=2.659, p=0.045, df=5) in cytosol (cyt) and mitochondria (MT) isolated from control and IL13 treated HAEC, Mean ± SD, n=5 (each from a different donor). **d** Accumulation of alkenyl-P-HpETE-PE (left panel, MT vs MT+IL13, unpaired two-sided Student's t-test, t=2.838, p=0.0363, df=5; cyt vs cyt+IL13, unpaired two-sided Student's t-test, t=5.346, p=0.0031, df=5; cyt+IL13 vs MT+IL13, unpaired two-sided Student's t-test, t=2.669, p=0.0444, df=5) and diacyl-P-HpETE-PE (right panel, MT vs MT+IL13, unpaired two-sided Student's t-test, t=3.054, p=0.0283, df=5; cyt vs cyt+IL13, unpaired two-sided Student's t-test, t=3.068, p=0.0278, df=5) in cytosol (cyt) and mitochondria (MT) isolated from control and IL13 treated HAEC, Mean ± SD, n=5 (each from a different donor). **e** Contents of diacyl- and alkenyl-O-HpETE-PE (left panel, paired two-sided Student's t-test, t=10.21, p=0.0020, df=3) and diacyl- and alkenyl-O-HpETE-PC (right panel) in 15-LOX-1 deficient HAEC exposed to IL13, Mean ± SD, n=4 (each from a different donor). **f** Contents of diacyl- and alkenyl-S-HpETE-PC (left panel, paired two-sided Student's t-test, t=10.21, p=0.0020, df=3) and (**g**) diacyl- and alkenyl-O-HpETE-PC (right panel) in 15-LOX-1 deficient HAEC exposed to IL13, Mean ± SD, n=4 (each from a different donor).

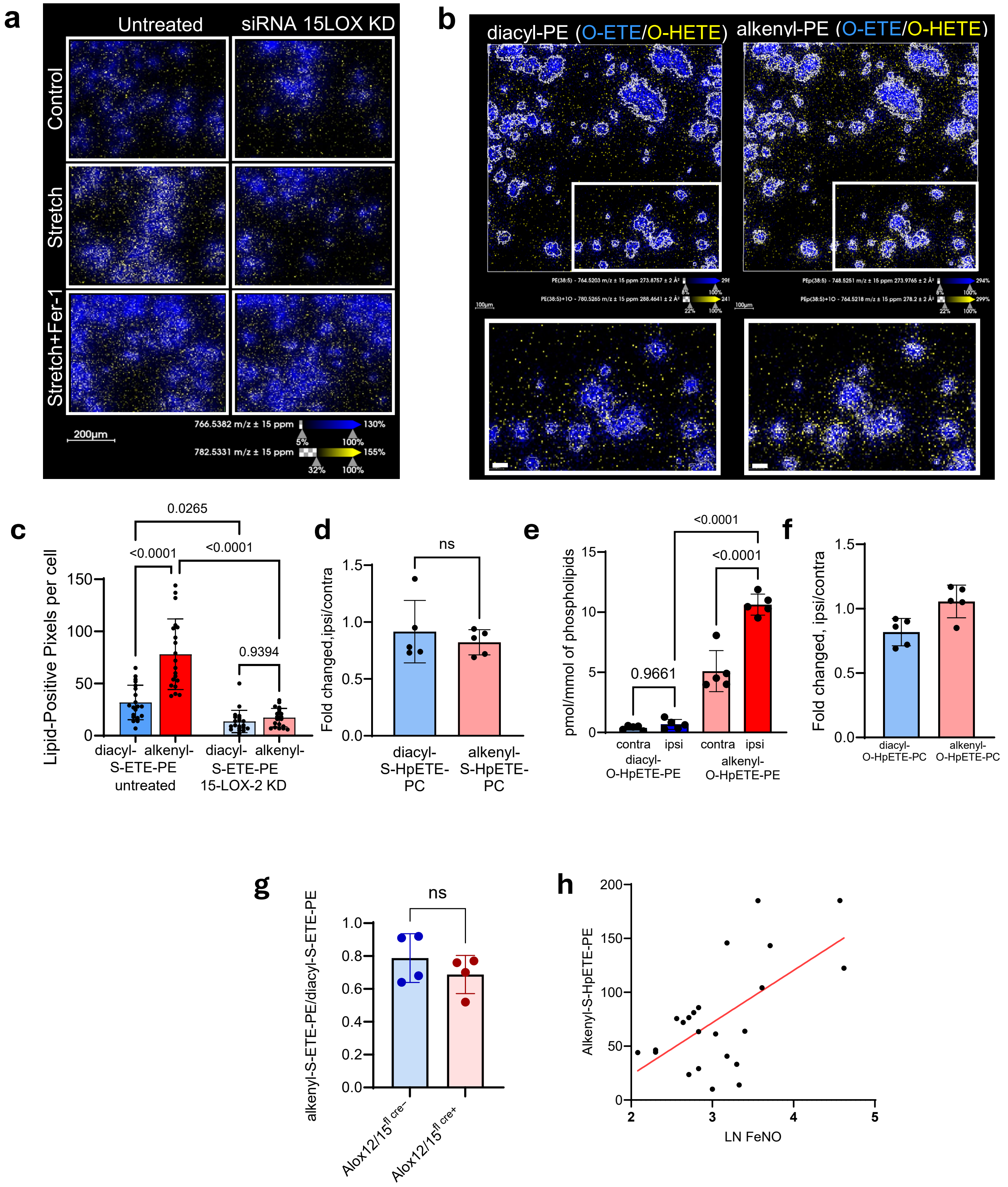

Supplementary Fig. 10.

Supplementary Fig. 10. Assessment of diacyl- and alkenyl-ETE-PE species in mouse brain after CCI, in PMN-MDSC from tumor bearing mice and in HAECs from asthma patients.

a timsOFF MALDI-MSI images of HT22 cells under six conditions: untreated control, stretch, and stretch + Ferrostatin-1 (Fer-1); and 15-LOX-2 KD control, stretch, and stretch + Fer-1. Images show the spatial distribution of two overlaid lipid species within ±15 ppm of their theoretical m/z values: diacyl-S-ETE-PE (PE38:4, m/z 766.5382, blue) and diacyl-S-HETE-PE (PE38:4+1[O], m/z 782.5331, yellow). Scale bar: 200 µm. **b** timsON MALDI-MSI of injured HT22 cells (untreated, stretch), enabling separation of isobaric species by collision cross section (CCS). Left: diacyl-O-ETE-PE (PE(38:5), m/z 764.5203 ± 15 ppm, blue; CCS = 273.87 ± 2Å) overlaid with diacyl-O-HETE-PE (PE(38:5)+1[O], m/z 780.5265 ± 15 ppm, yellow; CCS = 288.46 ± 2Å). Scale bars: 100 µm; bottom insets: zoomed images with a 50 µm scale bar, showing higher-resolution lipid distribution at the single-cell level. Right: alkenyl-O-ETE-PE (PE(38:5), m/z 748.5251 ± 15 ppm, blue; CCS = 273.97 ± 2Å) overlaid with alkenyl-O-HETE-PE (PE(38:5)+1[O], m/z 764.5218 ± 15 ppm, yellow; CCS = 278.20 ± 2Å). Scale bars: 100 µm; bottom insets: zoomed images with a 50 µm scale bar, showing higher-resolution lipid distribution at the single-cell level. **c** Quantification of O-HETE-PEs abundance at the single-cell level. The number of lipid-positive pixels per cell was measured within segmentation-defined cell boundaries (SCiLS Lab). Left: untreated, stretch condition. Right: siRNA 15-LOX KD stretch condition. Bars show mean pixel count ± SD (n=20 cells) for each oxidized lipid species. One-way ANOVA (F=43.38, $p < 0.0001$, df=79). **d** Changes in the content of diacyl- and alkenyl-S-HpETE-PC in ipsilateral cortical areas. Data presented as fold change, n=5 (five different mice). Mean ± SD, unpaired two-sided Student's t-test revealed no significant (ns) differences. **e** Contents of O-HpETE-PEs in mouse brain after CCI. Data are Mean ± SD, n=5 (each from a different mouse), ordinary one-way ANOVA (F=119.0, $p<0.0001$, df=19), Tukey's multiple comparisons p-values are shown on the plot. **f** Content of diacyl- and alkenyl-O-HpETE-PC in ipsilateral cortical area. Data presented as fold change respectively to diacyl- and alkenyl-O-HpETE-PC detected in contralateral cortical area. Mean ± SD, n=5, (each from a different mouse). **g** Content of alkenyl- and diacyl-S-ETE-PE in tumor PMN MDSC obtained from LCC bearing ALOX12/15 deficient mice. Mean ± SD, n=4 (each from a different mouse), unpaired two-sided Student's t-test revealed no significant change (ns). **h** Intracellular levels of alkenyl-S-HpETE-PE in freshly brushed human airway epithelial cells from asthmatic and healthy participants correlate with NO levels, measured as fractional exhaled NO (FeNO), in parts per billion of exhaled breath. This relationship supports interactions between alkenyl-S-HpETE-PE and a known biomarker of Type-2 (T2) Hi asthma.

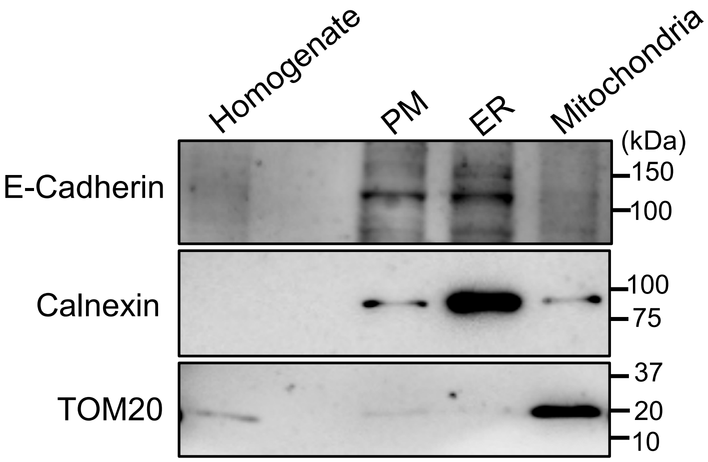

Supplementary Fig. 11. Western blot analysis to estimate marker proteins in fractions sucrose gradient ultracentrifugation.

10 µg of protein was loaded and enrichment of PM, ER and Mitochondria was confirmed by using antibodies specific for PM (E-cadherin), ER (calnexin) and mitochondria (TOM20). Western were repeated 3 times (from 3 independent experiments) with similar results.

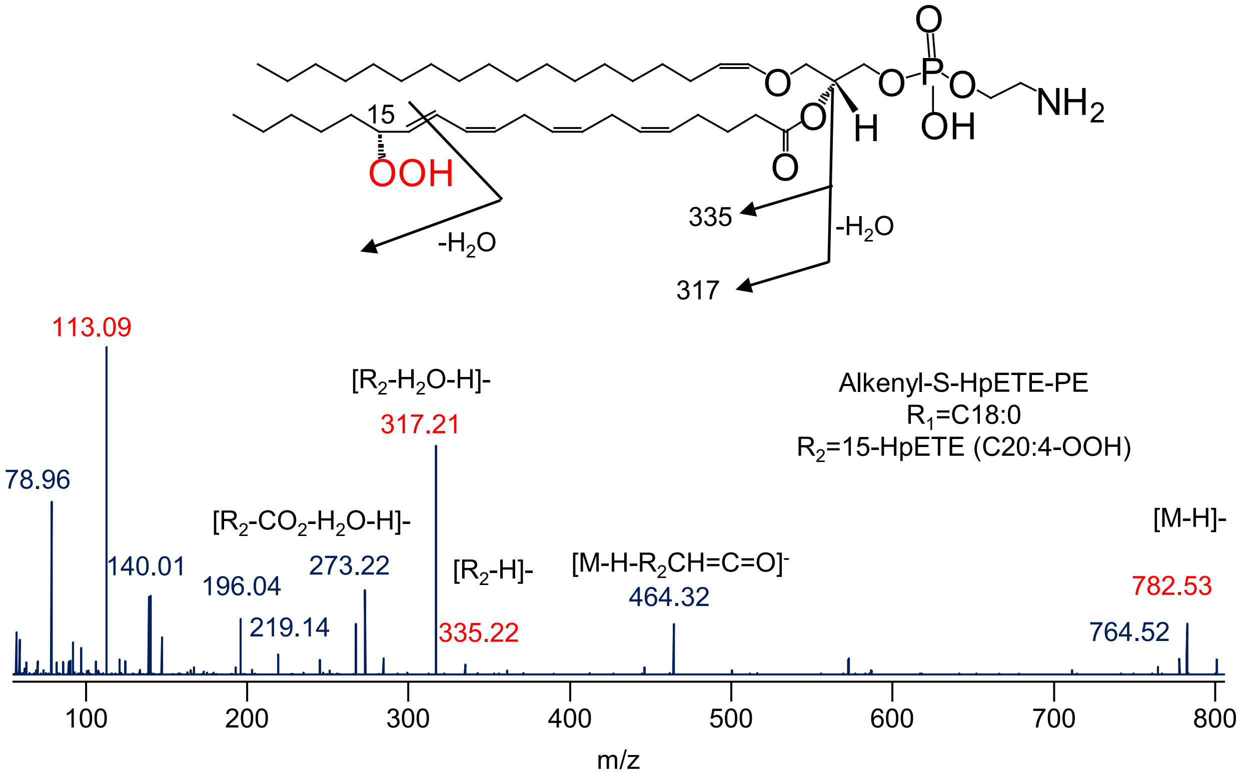

Supplementary Fig. 12. MS^2 spectrum of alkenyl-S-15-HpETE-PE synthetized using by 15-LOX-2.

Structure of alkenyl-S-15-HpETE-PE is inserted.

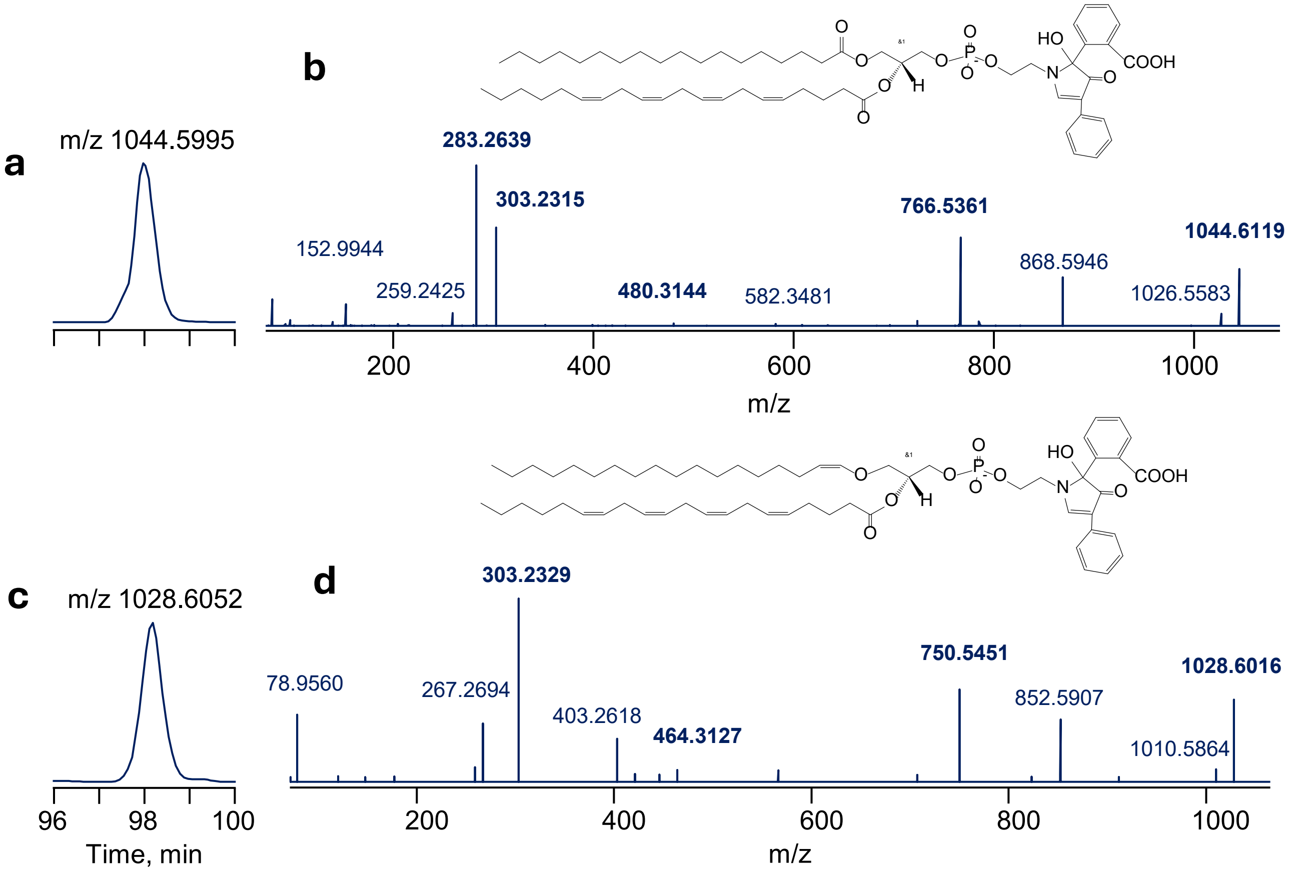

Supplementary Fig. 13. Typical LC-MS profiles (a,c) and MS^2 spectra (b,d) of fluorescamine labeled diacyl-S-ETE-PE (upper panels)) and alkenyl-S-ETE-PE (lower panels).

Possible structures of fluorescamine labeled PEs are inserted. To estimate the distribution of arachidonoyl PE diacyl and alkenyl-acyl molecular species on the cell surface we applied fluorescent labelled dye -fluorescamine which is widely used for fluorescence staining of living cells (*Poccia DL, Palevitz BA, Campisi J, Lyman H Fluorescence staining of living cells with fluorescamine. Protoplasma, 1979, 98, 91—113*).

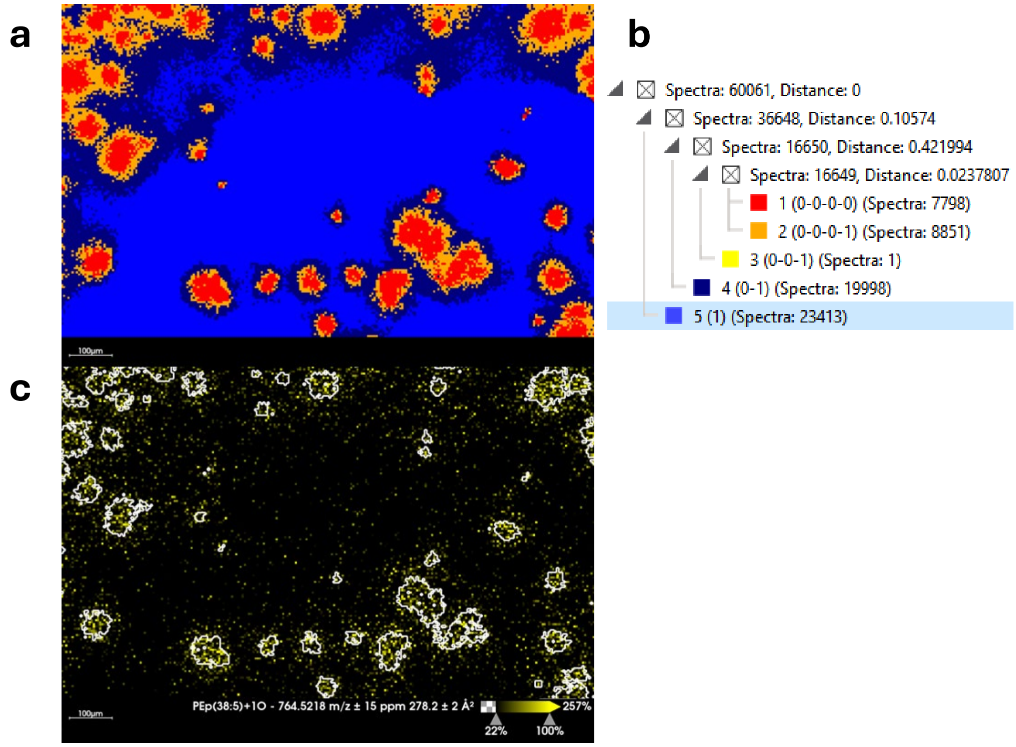

Supplementary Fig. 14. Segmentation-Based Identification of Cellular Regions for HT22 cells Using Lipid Distribution Patterns.

a Segmentation analysis was performed in SCiLS Lab using the automated feature-finding tool, based on the spatial distribution of 804 lipid species within the m/z range 650–950. **b** A segmentation tree, generated via multivariate clustering of mass spectra, grouped pixels with similar lipid signatures, enabling the identification of spatially distinct cellular regions. Red clusters in the segmentation map correspond to probable cell borders. **c** The corresponding lipid profile extracted from these border regions reveals distinct phospholipid compositions, confirming their cellular localization. The distribution of alkenyl-HETE-PE (PE(38:5)+1[O]) for untreated, stretch condition, enriched at the identified borders, further supports the segmentation-based identification of cell outlines.

a

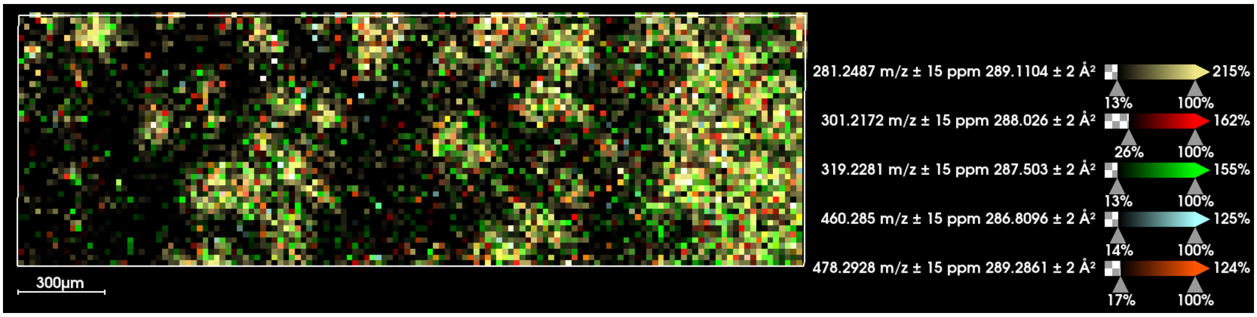

**m/z**	**Ion Description**
**281.2487**	Loss of sn2-18:1
**301.2172**	Loss of sn2-20:4-OOH minus H_2O
**319.2281**	Loss of sn2-20:4-OOH
**460.2850**	Neutral loss of sn2-20:4-OOH
**478.2928**	Loss of sn2 sn2-20:4-OOH as ketene (RCH=C=O)

b

m/z	1/K0 [V·s/cm^2]	CCS [Å^2]	Name
766.5339	1.3532	275.8893	*PE(38:4)
782.5309	1.4148	288.3351	*PE(38:4)+1[O]
764.5203	1.3433	273.8757	PE(38:5)
780.5265	1.4154	288.4641	*PE(38:5)+1[O]
750.5411	1.3488	275.0827	PEp(38:4)
766.5356	1.3787	281.0755	*PEp(38:4)+1[O]
748.5251	1.3433	273.9765	PEp(38:5)
764.5218	1.3645	278.2000	*PEp(38:5)+1[O]
780.5149	1.3685	278.9094	*PEp(38:5)+2[O]

Supplementary Fig. 15. MALDI MS/MS imaging confirmation and CCS values of oxidized PE species in HT22 cells.

a *Upper panel*: MALDI MS/MS imaging of diacyl-PE(38:5)+1[O] in untreated injured HT22 cells performed with timsON separation, showing a measured CCS = 288 ± 2 Å². *Lower panel*: Annotated characteristic fragment ions (m/z values) confirming the oxidized PE structure. **b** Table summarizing experimentally determined m/z and CCS values for identified diacyl- and alkenyl-ETE-PE species. Species confirmed by MALDI MS/MS imaging with timsON separation are indicated with asterisks (*).

Supplementary Table 1. Demographics data for human participants (HAEC cells).

	Healthy controls	Mild-Moderate Asthma	Severe Asthma
Age*	47±7	41±19	51±14
Sex/Gender (woman/man)	5/3	5/0	7/3
Race (White/Black)	7/1	5/0	10/0
FeNO (ppb)#	17 (14-19)	21 (9-29)	30 (16-55)

*mean±standard deviation
#median with intequartile range

Supplementary Table 2. Demographics data for human participants (Samples were obtained from Asthma patients).

Patient ID	Sex	Race	Age	Subject type
IMSA-090	Female	White	48	HC
IMSA-091	Male	White	39	HC
IMSA-093	Male	White	38	HC
IMSA-102	Female	White	46	HC
IMSA-243	Female	White	60	HC
IMSA-254	Male	White	44	HC
IMSA-104	Female	White	32	MMA
IMSA-105	Female	White	33	MMA
IMSA-107	Female	White	58	MMA
MISA-231	Female	White	20	MMA
IMSA-234	Female	White	64	MMA
IMSA-108	Female	White	64	SA
IMSA-110	Male	White	30	SA
IMSA-094	Male	White	58	SA
IMSA-101	Female	White	56	SA
IMSA-109	Female	White	52	SA
IMSA-225	Female	White	58	SA
IMSA-248	Female	White	22	SA
IMSA-251	Male	White	65	SA
IMSA-232	Female	White	57	SA
SARP-106	Female	White	46	SA
SARP-137	Female	White	52	HC
EHF	Female	Black	47	HC
