# Supplementary material for: 15-LOX-catalytic bias towards ether-(alkenyl)-ETE-PEs oxidation bestows selectivity of PRO-ferroptotic cell death signaling
Source: Nat Commun. 2026 Jun 17;17:5360. doi: 10.1038/s41467-026-71869-z (PMC13276253; doi:10.1038/s41467-026-71869-z)
Supplement: Supplementary file 2 — Description of Additional Supplementary Files [file 41467_2026_71869_MOESM2_ESM.pdf]

Description of Additional Supplementary Files

Supplementary Movie 1. All atom molecular dynamics simulation of alkenyl-PE bound to the catalytic site of human 15-LOX-2, showing the first independent trajectory of the system. The sn-1 acyl chain of alkenyl-PE is colored in brown and the sn-2 chain in green. Iron and the catalytic histidines are displayed as a pink sphere and silver sticks, respectively. Total simulation time is 300 ns; the movie was captured every 1 ns.

Supplementary Movie 2. All atom molecular dynamics simulation of alkenyl-PE bound to the catalytic site of human 15LOX-2, showing the second independent trajectory of the system. The sn-1 acyl chain of alkenyl-PE is colored in brown and the sn-2 chain in green. Iron and the catalytic histidines are displayed as a pink sphere and silver sticks, respectively. Total simulation time is 300 ns; the movie was captured every 1 ns.

Supplementary Movie 3. All atom molecular dynamics simulation of alkenyl-PC bound to the catalytic site of human 15-LOX-2, showing the first independent trajectory of the system. The sn-1 acyl chain of alkenyl-PC is colored in brown and the sn-2 chain in green. Iron and the catalytic histidines are displayed as a pink sphere and silver sticks, respectively. Total simulation time is 300 ns; the movie was captured every 1 ns.

Supplementary Movie 4. All atom molecular dynamics simulation of alkenyl-PC bound to the catalytic site of human 15-LOX-2, showing the second independent trajectory of the system. The sn-1 acyl chain of alkenyl-PC is colored in brown and the sn-2 chain in green. Iron and the catalytic histidines are displayed as a pink sphere and silver sticks, respectively. Total simulation time is 300 ns; the movie was captured every 1 ns.

Supplementary Movie 5. All atom molecular dynamics simulation of alkenyl-PE bound to the catalytic site of human 15-LOX-2 in a complex with PEBP1, showing the first independent trajectory of the system. The sn-1 acyl chain of alkenyl-PE is colored in brown and the sn-2 chain in green. Iron and the catalytic histidines are displayed as a pink sphere and silver sticks, respectively. Total simulation time is 300 ns; the movie was captured every 1 ns.

Supplementary Movie 6. All atom molecular dynamics simulation of alkenyl-PE bound to the catalytic site of human 15-LOX-2 in a complex with PEBP1, showing the second independent trajectory of the system. The sn-1 acyl chain of alkenyl-PE is colored in brown and the sn-2 chain in green. Iron and the catalytic histidines are displayed as a pink sphere and silver sticks, respectively. Total simulation time is 300 ns; the movie was captured every 1 ns.
