# Supplementary material for: 15-LOX-catalytic bias towards ether-(alkenyl)-ETE-PEs oxidation bestows selectivity of PRO-ferroptotic cell death signaling
Source: Nat Commun. 2026 Jun 17;17:5360. doi: 10.1038/s41467-026-71869-z (PMC13276253; doi:10.1038/s41467-026-71869-z)
Supplement: Supplementary file 9 — Reporting Summary [file 41467_2026_71869_MOESM9_ESM.pdf]

nature portfolio

Corresponding author(s): NCOMMS-25-08516B Valerian E. Kagan

Last updated by author(s): Mar 25, 2026

Reporting Summary

Nature Portfolio wishes to improve the reproducibility of the work that we publish. This form provides structure for consistency and transparency in reporting. For further information on Nature Portfolio policies, see our Editorial Policies and the Editorial Policy Checklist.

Statistics

For all statistical analyses, confirm that the following items are present in the figure legend, table legend, main text, or Methods section.

n/a	Confirmed	
☐	☒	The exact sample size (*n*) for each experimental group/condition, given as a discrete number and unit of measurement
☐	☒	A statement on whether measurements were taken from distinct samples or whether the same sample was measured repeatedly
☐	☒	The statistical test(s) used AND whether they are one- or two-sided<br>*Only common tests should be described solely by name; describe more complex techniques in the Methods section.*
☒	☐	A description of all covariates tested
☒	☐	A description of any assumptions or corrections, such as tests of normality and adjustment for multiple comparisons
☐	☒	A full description of the statistical parameters including central tendency (e.g. means) or other basic estimates (e.g. regression coefficient) AND variation (e.g. standard deviation) or associated estimates of uncertainty (e.g. confidence intervals)
☐	☒	For null hypothesis testing, the test statistic (e.g. *F*, *t*, *r*) with confidence intervals, effect sizes, degrees of freedom and *P* value noted<br>*Give P values as exact values whenever suitable.*
☒	☐	For Bayesian analysis, information on the choice of priors and Markov chain Monte Carlo settings
☒	☐	For hierarchical and complex designs, identification of the appropriate level for tests and full reporting of outcomes
☒	☐	Estimates of effect sizes (e.g. Cohen's *d*, Pearson's *r*), indicating how they were calculated

Our web collection on statistics for biologists contains articles on many of the points above.

Software and code

Policy information about availability of computer code

Data collection

NAMD: A parallel, object-oriented molecular dynamics code designed for high-performance simulations of large biomolecular systems. NAMD 2.14 for CUDA was used to perform all-atom molecular dynamics simulations of 15LOX. The software is available for academic research purposes at https://www.ks.uiuc.edu/Research/namd/
SMINA: A fork of AutoDock Vina for protein-ligand docking prediction was used to obtain the initial structure of alkenyl-ETE-PE structures for molecular dynamics simulations. The tool is available at https://sourceforge.net/projects/smina/
Gaussian: A computational chemistry program, version g03, was used to obtain partial charges and geometry for the catalytic site.
Maestro: The modeling environment is provided by the Schrodinger package. It was used to prepare substrates (building the structure and minimize it).
SwissParam: Web tool that provides topology and parameters for small organic molecules. It was used to parametrize the alkenyl-ETE-PE structure topology and parameters for the CHARMM force field used in molecular dynamics simulations. The server is available at: http://www.swissparam.ch/

Data analysis

VMD: A popular molecular graphics program was used to analyze and visualize trajectories generated by the NAMD package. VMD 1.93 was used. The software is available for academic research purposes at https://www.ks.uiuc.edu/Research/vmd/
ProDy: It is a free and open-source Python package for the analysis of protein dynamics. ProDy 2.4.1 was used to analyze the trajectories from molecular dynamics simulations. The software is available at http://www.bahargroup.org/prody/
Mass spectrometry data were analyzed using Compound Discoverer 2.0, Xcalibur 4.0, SCiLS Lab 2025a Pro, and Microsoft Excel 2016.
Graphs were plotted using GraphPad Prism 10 and Origin 2022b. Statistical analyses were performed by using GraphPad Prism 10 software.

For manuscripts utilizing custom algorithms or software that are central to the research but not yet described in published literature, software must be made available to editors and reviewers. We strongly encourage code deposition in a community repository (e.g. GitHub). See the Nature Portfolio guidelines for submitting code & software for further information.

Data

Policy information about availability of data

All manuscripts must include a data availability statement. This statement should provide the following information, where applicable:

- Accession codes, unique identifiers, or web links for publicly available datasets
- A description of any restrictions on data availability
- For clinical datasets or third party data, please ensure that the statement adheres to our policy

Data generated during the study and included in this article are available from the corresponding authors upon request. Source data are provided with this paper.

Research involving human participants, their data, or biological material

Policy information about studies with human participants or human data. See also policy information about sex, gender (identity/presentation), and sexual orientation and race, ethnicity and racism.

Reporting on sex and gender	For the in viro experiments on human airway epithelial cells, typically with 3-6 samples obtained from individual human subjects, the biological sex data are included in demographic tables 1-3, the sexual orientation was not reported.
Reporting on race, ethnicity, or other socially relevant groupings	For the in vitro experiments on human airway epithelial cells, typically with 3-6 samples obtained from individual human subjects, race, ethnicity or other socially relevant grouping were not reported. Demographic data included in Supplementary tables 1-2 Tissue samples of the normal human skin were collected from three white males, ages 67-75, after obtaining signed informed consent.
Population characteristics	Human airway epithelial cells obtained from patients with severe and moderate asthma were used. The definition of asthma as severe and moderate are based on definition from the European Respiratory Society and American Thoracic Society. The references are included in the paper. These definitions are based on clinical symptoms, exacerbations, lung function and amount of medication used. healthy subjects were those without an y evidence of respiratory (or other chronic diseases and have a normal lung function. No any potential self-selection biases were presented. Tables with demographics data (Supplementary Tables 1 and 2) are included in Supplementary Information file.
Recruitment	Human subjects were recruited through the University of Pittsburgh Clinical and Translational Science Institute registry, The University of Pittsburgh Asthma and Environmental Lung Health registry, as well as through the pulmonary clinics. All participants were recruited as part of the Immune-epithelial Cell Interactions in Severe Asthma (P01 AI106684 and P01AI106684-06A1) .<br>The study of the collected normal human skin tissue (three white males, ages 67-75) was approved by the Institutional Review Board of the University of Pittsburgh (PRO15100580).
Ethics oversight	University of Pittsburgh Institutional Review Board

Note that full information on the approval of the study protocol must also be provided in the manuscript.

Field-specific reporting

Please select the one below that is the best fit for your research. If you are not sure, read the appropriate sections before making your selection.

☒ Life sciences ☐ Behavioural & social sciences ☐ Ecological, evolutionary & environmental sciences

For a reference copy of the document with all sections, see nature.com/documents/nr-reporting-summary-flat.pdf

Life sciences study design

All studies must disclose on these points even when the disclosure is negative.

Sample size	No statistical methods were used to pre-determine sample sizes, but our sample sizes are similar to those reported in previous publications. The chosen sample size produce suitable standard deviation.
Data exclusions	No data were excluded from analysis.
Replication	Model experiments were prepared/repeated independently at least 3 times, other experiments (in vitro and in vivo) were repeated 3 or more times as indicated in the figure legends. All attempts at replication of experimental findings were successful.
Randomization	All samples and animals were randomly allocated into experimental groups.
Blinding	Investigators were blinded to group allocation during data collection.

Reporting for specific materials, systems and methods

We require information from authors about some types of materials, experimental systems and methods used in many studies. Here, indicate whether each material, system or method listed is relevant to your study. If you are not sure if a list item applies to your research, read the appropriate section before selecting a response.

Materials & experimental systems

n/a	Involved in the study
☐	☒ Antibodies
☐	☒ Eukaryotic cell lines
☒	☐ Palaeontology and archaeology
☐	☒ Animals and other organisms
☐	☒ Clinical data
☒	☐ Dual use research of concern
☒	☐ Plants

Methods

n/a	Involved in the study
☒	☐ ChIP-seq
☒	☐ Flow cytometry
☒	☐ MRI-based neuroimaging

Antibodies

Antibodies used	Antibodies against 15LOX1 (rabbit IgG) were from Abnova (cat # H00000246-D01PWalnut, CA). Primary antibodies against 15LOX2 (Cat # sc-271290), E-cadherin (Cat # sc-8426), and Tom20 (Cat # sc-17764) were from Santa Cruz Biotechnology Inc. (Dallas, TX). Secondary antibodies (cat # 7076S) were from Cell Signaling Technology, Inc. (cat # 7076S, Danvers, MA). Beta-actin HRP conjugated antibodies were from Abcam (cat # ab20272, Cambridge, MA. The total OXHPOS (mouse IgG) were from Abcam (cat # ab110411, Cambridge, MA). The anti-GAPDH antibodies were from Novus Biologicals (cat# NB300-320, Littleton, CO). The anti-AGPS antibody was from Proteintech (cat # 21011-1-AP, Rosemont, IL), as was anti-Calnexin (Cat # 10427-2-AP). Anti-TMEM189 and anti-TMEM164 were from Thermo Fisher (Catalog #PA5-97031 and PA5-58540, respectively).
Validation	All antibodies used in this study were commercially available and validated by manufacturers.

Eukaryotic cell lines

Policy information about cell lines and Sex and Gender in Research

Cell line source(s)	Murine lung epithelial cell line (MLE), mouse embryonic fibroblasts (MEF) A375, HT1080 cells, primary human epidermal keratinocytes (HEK) were obtained from American Type Culture Collection (ATCC). GPX4-/- A375 cells were obtained from D. Wei Gu (Columbia University, NY). The HT-22 cell line was obtained from Dr. David Schubert, The Salk Institute (La Jolla, CA). HBE cells (a human bronchial epithelial cell line originally established by Dieter Gruenert).<br>Primary Human Airway Epithelial Cell Culture were obtained from human subjects (see the section "Research involving human participants, their data, or biological material" above).<br>Bone marrow derived macrophages were isolated from bones of mice and were approved by the Institutional Animal Care and Use Committee of AstraZeneca (Gaithersburg, MD) and conducted in Association for Assessment and Accreditation of Laboratory Animal Care (AAALAC)–accredited and United States Department of Agriculture (USDA)–licensed facility and the AstraZeneca Global Bioethics policy. No primary cell lines from human or vertebrate models were established.
Authentication	None of the cell lines used were authenticated.
Mycoplasma contamination	All cell lines were tested negatively for Mycoplasma contamination.
Commonly misidentified lines (See ICLAC register)	No commonly misidentified lines were used in this study.

Animals and other research organisms

Policy information about studies involving animals; ARRIVE guidelines recommended for reporting animal research, and Sex and Gender in Research

Laboratory animals	C57BL6J mice 12–15 weeks of age (Jackson Laboratories, Bar Harbor, ME), Alox15fl mice (Jackson Laboratory) were with B6.Cg-Tg (S100 A8-cre, -EGFP) 1Ilw/J (Jackson Laboratory).
Wild animals	Study didn't involve wild animals.
Reporting on sex	Male C57BL6J mice were used in CCI experiments, Female Alox12/15fl mice were used in mouse tumor model to isolate bone marrow macrophages.
Field-collected samples	Study didn't involve samples collected from field.
Ethics oversight	All animal experimental procedures were conducted in strict accordance with the recommendation in the guide for the Care and Use of Laboratory Animals of the National Institute of Health and approved by the Institutional Animal Care and Use Committees of University of Pittsburgh and Columbia University Institutional Animal Care and Use Committee (IACUC) , as well as the Wistar Institute Animal Care and Use Committee and he Institutional Animal Care and Use Committee of AstraZeneca (Gaithersburg, MD)

	and conducted in Association for Assessment and Accreditation of Laboratory Animal Care (AAALAC)–accredited and United States Department of Agriculture (USDA)–licensed facility and the AstraZeneca Global Bioethics policy.

Note that full information on the approval of the study protocol must also be provided in the manuscript.

Clinical data

Policy information about clinical studies

All manuscripts should comply with the ICMJE guidelines for publication of clinical research and a completed CONSORT checklist must be included with all submissions.

Clinical trial registration	Since this is not a clinical trial, there is no registration.
Study protocol	The study protocol can be provided on request. This is an observation study which focus on collection of lung-specific biologica; samples (Immune mechanism of severe asthma /NIAID and Protein-oxidized phospholipid interactions determine epithelial cell fate and asthma control/NIAID)
Data collection	Samples were collected as a part of IRB approved research. Bronchoscopies with associated clinical visits, asthma control questionnaires, spirometry and asthma related clinical history were obtained.
Outcomes	There is no primary outcome because this is an exploratory study on the role of 15LOX in oxidation of plasmalogens during ferroptosis.

Plants

Seed stocks	N/A
Novel plant genotypes	N/A
Authentication	N/A
