# Supplementary material for: 15-LOX-catalytic bias towards ether-(alkenyl)-ETE-PEs oxidation bestows selectivity of PRO-ferroptotic cell death signaling
Source: Nat Commun. 2026 Jun 17;17:5360. doi: 10.1038/s41467-026-71869-z (PMC13276253; doi:10.1038/s41467-026-71869-z)
Supplement: Supplementary file 10 — Transparent Peer Review file [file 41467_2026_71869_MOESM10_ESM.pdf]

nature portfolio

Peer Review File

15-LOX-CATALYTIC BIAS TOWARDS ETHER-(ALKENYL)-ETE-PEs OXIDATION BESTOWS SELECTIVITY OF PRO-FERROPTOTIC CELL DEATH SIGNALING.

Corresponding Author: Professor Valerian E Kagan

This file contains all reviewer reports in order by version, followed by all author rebuttals in order by version.

Version 0:

Reviewer comments:

Reviewer #1

(Remarks to the Author)
In this manuscript, Tyurina Y.T. et al. demonstrated that 15-LOX selectively catalyzes the peroxidation of alkenyl-ETE-PEs over diacyl-ETE-PEs, promoting ferroptosis through the accumulation of alkenyl-15-HpETE-PEs. The authors primarily investigated the formation of diacyl- and alkenyl-HpETE-PEs compared to PCs in the presence of their respective substrates and 15-LOX in vitro (liposomes) and in disease models using cell and tissue samples.
This manuscript interests readers, as emerging studies have recently highlighted the role of ether phospholipids in driving ferroptosis. The 15-LOX-catalyzed alkenyl-ETE-PE peroxidation axis and its role in ferroptosis may identify new oxidized lipid species as potential targets for ferroptosis regulation. However, certain gaps remain, and the referee raises the following concerns and questions that need to be addressed:

1. The authors mention that most of the lipids in certain types of cells are assumed to contain primarily alkenyl-type phospholipids (Fig. 1). However, several studies have already measured intracellular ether-type phospholipids (e.g., doi.org/10.1038/s41589-020-0529-6). This difference should be discussed.

2. Dixon et al. indicated that “while the key ether lipid synthesis enzyme alkylglycerone phosphate synthase (AGPS) is entirely dispensable for ferroptosis, at least in some contexts” (doi.org/10.1016/j.chembiol.2022.06.004).
KO or KD experiments of such key enzymes are necessary to demonstrate the involvement of ether-type phospholipids in ferroptosis in this study. Alternatively, are ether-type phospholipids involved in ferroptosis only under specific conditions?

3. Plasmalogens are synthesized in peroxisomes and the ER and are localized in the inner leaflet of the plasma membrane, sensitizing cells to ferroptosis. However, the authors only measured alkenyl-ETE-PE in the mitochondria and cytosol of HAECs.

4. The authors emphasize that alkenyl-ETE-PE is a selective substrate of 15-LOX, leading to the production of alkenyl-HpETE-PE, which induces ferroptosis. However, based on the Human Protein Atlas database (https://www.proteinatlas.org/), the site of expression of 15LOX in vivo is not ubiquitous. Does this ensure the general involvement of LOX in the ferroptosis suggested by the authors?

5. The authors used MD simulations to investigate the binding activity between 15-LOX2 and alkenyl-ETE-PE/PC. However, this binding activity remains to be validated in vitro (e.g., using SPR) or in cells. Additionally, does the addition of PEBP1 induce a conformational change in the catalytic site of 15-LOX? If so, why did the formation of alkenyl-SA-HpETE-PE remain unchanged after modification by PEBP1 (Extended Fig. 3e)?

6. The authors used IL-13, UVB, and CCI as disease models to manipulate the expression of 15-LOX-1 and 15-LOX-2. However, these inducers may have additional off-target effects that influence the overall results (e.g., IL-13 facilitates ferroptosis via the IL-13-STAT6-SOCS1-SLC7A11 pathway in asthma). Genetically manipulating 15-LOX via KD, KO, or overexpression would be a more appropriate model for studying the distinct role of the 15-LOX-catalyzed alkenyl-ETE-PE peroxidation axis in ferroptosis. Also, to what extent does phospholipid oxidation by LOX contribute to cell death induction?

7. Fig2 h,i: Is cell death suppressed by knockout of 15LOX? Is the oxidation of these lipids the cause of cell death?

8. Fig4b: Increasing LOX expression with IL-13 alone does not facilitate ferroptosis, and ferroptosis is not enhanced without the addition of RSL3 and ETE. Does this mean that the oxidation of endogenous alkenyl-ETE-PE is insufficient to induce ferroptosis?

9. Why does cell death occur when cytoplasmic lipids are oxidized, and what is the state and localization of phospholipids in the cytoplasm? Also, does cell death occur when cells are treated with oxidized alkenyl-ETE-PE?

10. Fig 6: A comparison with the group without CCI treatment is needed. Is alkenyl-ETE-PE oxidation essential for causing CCI, or does it simply increase during disease progression? Is alkenyl-ETE-PE oxidation similarly important for disease progression in an asthma model?

Reviewer #2

(Remarks to the Author)
Comments:
• The authors suggest that there are two phases in ferroptosis: first phase mediated by LOX and second driven by non-enzymatic oxidation. What is the impact of each mechanism in generation of alkenyl-peroxides and diacyl-peroxides? Please discuss.
• The experiments with purified 15LOX were performed using liposomes with equal proportions of DOPC to diacyl-PE or DOPC to alkenyl-PE (50 : 50). Diacyl- and alkenyl-PEs have different properties in the bilayer, which potentially can influence the structure of liposomes thus leading to artificial differences in enzyme activity. Is it possible to repeat the experiment using a mixture of diacyl- and alkenyl-PEs substrate in the same liposome? Such an experiment is closer to the situation in cell membrane and can better characterize substrate preferences of purified 15LOX-1/2.
• Please explain in the discussion more clearly why PEBP1 did not stimulate peroxidation of alkenyl-PE.
• The data on asthma patients were obtained in extremely small groups (3 to 5). The conclusions could be strengthened by analyzing additional samples.

Reviewer #3

(Remarks to the Author)
Author reported alkyl/alkenyl-ETE-PE as potential substrates of enzymatically catalyzed peroxidation. By using redox lipidomics, biochemical, biophysical and genetic approaches combined with molecular dynamics simulations, we established that both isoforms of 15LOX (15LOX-1 and 15LOX-2) selectively oxidize alkyl/alkenyl-ETE-PE (but not 43 alkyl/alkenyl-ETE-PC) species to 15-HpETE-PEs and trigger ferroptotic cell death in vitro and in vivo more effectively than via oxidation of diacyl-ETE-PE. Thus, 15LOX-catalyzed bias towards oxidation of alkenyl-ETE-PE may represent a new therapeutic target. In my opinion this Manuscript should be consider following points.

1. The abstract lacks precision and depth. It should clearly define the problem, methodology, key findings, and conclusion in a more structured format for better reader comprehension.
2. The literature review section is insufficient and fails to provide a comprehensive background. It should include more recent and relevant studies to establish a strong foundation for the research problem.
3. The methodology is not well-justified. It is crucial to explain why the selected methods were chosen over other alternatives and how they are suitable for addressing the research question.
4. Figures and tables are not well-organized, and some are missing proper legends and labels. Ensure all data representations are clear and accompanied by sufficient explanations.
5. The statistical analysis is weak or incomplete. Provide detailed information about the statistical tests used, including the rationale behind their selection and their relevance to the data.
6. The results are not discussed in the context of existing literature. Compare the findings with similar studies to highlight the significance and novelty of the work.
7. There are several grammatical and typographical errors. A thorough proofreading is needed to improve the overall readability and professionalism of the manuscript.
8. The conclusion is too vague. Summarize the key findings more effectively and provide a clear statement on how the study advances knowledge in the field.
9. Some references are outdated, and a few citations are missing. Ensure that all references are current and properly formatted according to the journal’s guidelines.
10. The manuscript does not adequately address future research directions. Suggest specific areas where the study's findings could be further explored to strengthen the manuscript's impact.

Version 1:

Reviewer comments:

Reviewer #1

(Remarks to the Author)
The authors have performed additional experiments to address several certain gaps and questions from reviewers. However, further questions remain. The reviewer’s comments/questions are as follows:

1. The authors additionally show the level of diacyl- and alkenyl-PE in the plasma membrane of HBE cells, while HAECs were used for the determination of diacyl- and alkenyl-PE and PC in mitochondria and the cytosol. However, Fig. 1b and 1c showed different lipid profiles for these cells.

2. The enrichment of alkenyl-PE (especially alkenyl-ETE-PE) in several cell lines and tissues mainly resulted from TMEM164 as the authors mentioned? Are there any critical factors that influence this event? The new data demonstrated that AGPS and TMEM189 (catalytic enzymes in ether lipid biosynthesis) did not affect ferroptosis sensitivity. Why is that? The authors state that “The depletion of TMEM189 causes proportional increases in alkyl species analogous to the alkenyl species which are depleted, maintaining levels of PUFA-containing alkyl/alkenyl-PE” (page 9). Does the observation that ferroptosis sensitivity is unchanged in AGPS and TMEM189-KO cells indicate that, under RSL3 treatment, either alkyl-PE or alkenyl-PE is sufficient? If so, does this suggest that the importance of alkenyl-PE is limited to conditions under LOX induction?

3. Although the site and localization of ferroptosis is not the goal of this current study, it is still unclear why the author measured alkenyl-PE in the mitochondria as a representative organelle. How does mitochondria relate to ether-PL? If the authors wanted to demonstrate their levels in various organelles, there are several major sites for ferroptosis which more related to alkenyl-PE (such as ER or peroxisomes).

4. The author used the PE externalization and labeling method to determine diacyl- and alkenyl-PE in the plasma membrane. To measure total lipid contents in the plasma membrane, is this protocol appropriate for the current study compared to other protocols (e.g., PMID: 22555243)? So that the levels of PC can be also measured.

5. The authors performed additional HT-22 stretching experiments. However, the reviewer recommends that the role of alkenyl-HpETE-PE in ferroptosis upon Fer-1 treatment and 15-LOX-2 KD in TBI should be performed in CCI mice model (same as the utilization of genetically manipulated 15-LOX in PMN-MDSC).

6. More data (e.g., IL-13) linking 15-LOX expression, alkenyl-PE, and ferroptosis in asthmatic patients could improve clinical relevance and translational insight for this study.

Other comments

Several typographical errors and labeling inconsistencies remain throughout the revised manuscript. Examples include missing labels for Fig. 8g and Extended Data Figs. 9f–9g, and the incorrect notation “stretc + Fer-1” in Fig. 7f. The manuscript would benefit from thorough proofreading prior to resubmission.

Reviewer #2

(Remarks to the Author)

Reviewer #3

(Remarks to the Author)
The revised version of the manuscript includes all remarks and modifications indicated. The main concerns of the manuscript have been solved. In my opinion, the provided version is now suitable for publication.

Version 2:

Reviewer comments:

Reviewer #1

(Remarks to the Author)
The revised manuscript adequately addresses all of the comments and incorporates the suggested revisions. The major concerns raised previously have been satisfactorily resolved. In my opinion, the manuscript is now suitable for publication.

Open Access This Peer Review File is licensed under a Creative Commons Attribution 4.0 International License, which permits use, sharing, adaptation, distribution and reproduction in any medium or format, as long as you give appropriate credit to the original author(s) and the source, provide a link to the Creative Commons license, and indicate if changes were made.
In cases where reviewers are anonymous, credit should be given to 'Anonymous Referee' and the source.
The images or other third party material in this Peer Review File are included in the article's Creative Commons license, unless indicated otherwise in a credit line to the material. If material is not included in the article's Creative Commons license and your intended use is not permitted by statutory regulation or exceeds the permitted use, you will need to obtain permission directly from the copyright holder.
To view a copy of this license, visit https://creativecommons.org/licenses/by/4.0/

Comments from the Reviewers:

Reviewer #1 (Remarks to the Author): In this manuscript, Tyurina Y.Y. et al. demonstrated that 15-LOX selectively catalyzes the peroxidation of alkenyl-ETE-PEs over diacyl-ETE-PEs, promoting ferroptosis through the accumulation of alkenyl-15- HpETE-PEs. The authors primarily investigated the formation of diacyl- and alkenyl-HpETE-PEs compared to PCs in the presence of their respective substrates and 15-LOX in vitro (liposomes) and in disease models using cell and tissue samples. This manuscript interests readers, as emerging studies have recently highlighted the role of ether phospholipids in driving ferroptosis. The 15-LOX-catalyzed alkenyl-ETE-PE peroxidation axis and its role in ferroptosis may identify new oxidized lipid species as potential targets for ferroptosis regulation.

We thank the Reviewer for the encouraging comments emphasizing the emerging interest in the role of ether phospholipids in ferroptosis.

However, certain gaps remain, and the referee raises the following concerns and questions that need to be addressed:

1. The authors mention that most of the lipids in certain types of cells are assumed to contain primarily alkenyl-type phospholipids (Fig. 1). However, several studies have already measured intracellular ether-type phospholipids (e.g., doi.org/10.1038/s41589-020-0529-6). This difference should be discussed.

In some cells such as immune cells, platelets, alkenyl ether lipids (plasmalogens) containing polyunsaturated fatty acids, particularly arachidonic acid (ETE), in the sn-2 position are more abundant than their respective diacyl counterparts (PMID: 6712967) (https://doi.org/10.1007/BF02536106 [not Pubmed indexed], PMID: 6427378, PMID: 6417134). Furthermore, in red blood cells, the inner leaflet of the plasma membrane contains high levels of plasmalogens enriched in polyunsaturated residues, particularly ETE. (PMID: 32367017). This enrichment is achieved through enzymatic remodeling likely catalyzed by transmembrane protein 164 (TMEM164), which transfers ETE from diacyl-PC to alkenyl-lyso-PE as has been demonstrated in human cancer cell lines (PMID: 36782012). Our findings extend these results by showing that alkenyl-PE species are selectively enriched in ETE relative to both diacyl-PE and alkenyl-PC across various murine and human cells. We discussed the differences in the contents of primarily alkenyl-type phospholipids vs diacyl-phospholipids on page 13 of the revised manuscript. In addition, in the revised manuscript we included new data on the contents of diacyl-PE and alkenyl-PE in the plasma membrane of human bronchoalveolar (HBE) cells. Please see also our response to comment 3 of this Reviewer.

2. Dixon et al. indicated that "while the key ether lipid synthesis enzyme alkylglycerone phosphate synthase (AGPS) is entirely dispensable for ferroptosis, at least in some contexts" (doi.org/10.1016/j.chembiol.2022.06.004). KO or KD experiments of such key enzymes are necessary to demonstrate the involvement of ether type phospholipids in ferroptosis in this study. Alternatively, are ether-type phospholipids involved in ferroptosis only under specific conditions?

The Reviewer raises a very important point on the role of alkylglycerone phosphate synthase (AGPS) in ferroptosis. We addressed this issue by several sets of experiments. First, we compared the rates of oxidative modification of the vinyl bond in the sn-1 position of alkenyl-S-ETE-PE with peroxidation of sn-2 ETE in the same PE-plasmalogen. We employed enzymatic (15-LOX catalyzed) and non-enzymatic (2,2'-azobis(2,4-dimethylvaleronitrile) [AMVN]-dependent) inducers of peroxidation. With both triggers, peroxidation of ETE in the sn-2 position of PE occurred at a ~500-fold higher rate than oxidation of the vinyl-bond. This has been assessed by the accumulation of S-15-HpETE-PE with hydroperoxy-(OOH-) group in the sn-2-ETE and of 1-OOH-S-ETE-PE with OOH-group at the first carbon of the sn-1 stearic acid. Furthermore, the rate of sn-2-ETE-PE peroxidation induced by AMVN was not affected by the presence of the sn-1 vinyl-group and was approximately the same in alkenyl-S-ETE-PE and diacyl-S-ETE-PE. This is compatible with the interpretation that sn-2-ETE-residues rather than sn-1-vinyl-groups are the major determinants of pro-ferroptotic sensitivity. Finally, we found that RSL3 induced cell death in A375 melanoma cells is not affected by AGPS knockdown but is sensitive to supplementation with oleic acid or ETE. In the revised manuscript, these new results are included in the Results section (see Figures 2hi and Figure 4e-g) and described them on pages 6 and 9 of the revised manuscript, respectively.

3. Plasmalogens are synthesized in peroxisomes and the ER and are localized in the inner leaflet of the plasma membrane, sensitizing cells to ferroptosis. However, the authors only measured alkenyl-ETE-PE in the mitochondria and cytosol of HAECs.

In response to this comment, we performed new experiments in which we estimated the content of diacyl-S-ETE-PE and alkenyl-S-ETE-PE species in plasma membrane using human bronchoalveolar epithelial (HBE) cells. We were mostly interested in estimating the amounts of alkenyl-ETE-PE and diacyl-ETE-PE in the inner leaflet of the plasma membrane where they are available for peroxidation. PE is predominantly located in the inner leaflet of the plasma membrane due to its internalization by ATP dependent aminophospholipid translocase (APT) (PMID: 2288706). Inhibition of APT was achieved by depleting ATP using 2-deoxy-glucose (50 mM) in the presence of sodium azide (50 mM). This resulted in PE externalization to the membrane surface; we detected the externalized PE in the outer leaflet of the cell membrane using fluorescamine (200 μM, 30 seconds on ice) which interacts with primary amines on the cell surface (PMID: 486448). At the end of the incubation, lipids were extracted and two pools of PE - fluorescamine labeled and unlabeled - were resolved by LC/MS. We determined that the content of diacyl-S-ETE-PE species in the plasma membrane was higher compared to alkenyl-S-ETE-PE species (11.3±1.1 and 2.9±0.1 pmol/nmol of phospholipids, respectively). We added a new method to the Methods section (page 25), presented new results in Figure 1f and described them on page 5 of the revised manuscript.

4. The authors emphasize that alkenyl-ETE-PE is a selective substrate of 15-LOX, leading to the production of alkenyl-HpETE-PE, which induces ferroptosis. However, based on the Human Protein Atlas database (https://www.proteinatlas.org/), the site of expression of 15LOX in vivo is not ubiquitous. Does this ensure the general involvement of LOX in the ferroptosis suggested by the authors?

Various studies link 15-LOX to ferroptosis in several contexts, including asthma (PMID: 38641013; REF), cancer (PMID: 20234819), neurodegeneration (PMID: 33233496; PMID: 39292705), ischemia-reperfusion (PMID: 37154013; PMID: 36987924), and irradiation damage (PMID: 37327313). Initial studies suggested that 15-LOX drives lipid peroxidation, particularly PE oxidation (PMID: 27842066), resulting in activation of ferroptotic cell death (PMID: 36899940). Oxidation of PE by 15-LOX occurs in the cytosol where it is bound to intracellular membranes (PMID:9414270). Generation of hydroperoxy-PE species is specific for the initiation stage of ferroptosis. The second stage is 15-LOX independent and results in the formation of truncated phospholipids followed by the generation of protein adducts: electrophilic ox-truncated lipid species plus nucleophilic amino-acids (mainly Cys) in proteins (PMID: 37245287). We discussed this issue on page 15 of the revised manuscript.

5. The authors used MD simulations to investigate the binding activity between 15-LOX2 and alkenylETE-PE/PC. However, this binding activity remains to be validated in vitro (e.g., using SPR) or in cells. Additionally, does the addition of PEBP1 induce a conformational change in the catalytic site of 15-LOX? If so, why did the formation of alkenyl-SA-HpETE-PE remain unchanged after modification by PEBP1 (Extended Fig. 3e)?

Our previous work demonstrated that PEBP1 binds 15-LOX to facilitate its activity towards diacyl-PE oxidation (PMID: 29053969). To validate the effect of PEBP1 on 15-LOX-driven oxidation of alkenyl-PE and -PC, we knocked-down (KD) PEBP1 in HAEC cells and estimated the levels of oxidized diacyl- and alkenyl-S-ETE species of PE and PC. As expected, we found that the level of diacyl-S-HpETE-PE was dependent on the presence of PEBP1. Analysis of alkenyl-S-HpETE-PE demonstrated that its level in PEBP1 KD cells was not significantly different from those obtained from control cells. These data demonstrate that PEBP1 is not essential for 15-LOX catalyzed oxidation of alkenyl-PE species and correspond to the results obtained in the biochemical model and MD simulation data. Notably, the levels of diacyl-S-HpETE-PC in control cells were low and not different from those obtained from PEBP1 deficient cells. Alkenyl-S-HpETE-PC species were not detected either in control or in PEBP1 KD cells. We presented these results in Extended Data Figure 7b and described them in the text on page 8 of the revised manuscript.

As shown in previous simulations, PEBP1 binding to 15-LOX does not induce a critical conformational change within the catalytic site that accommodates the sn-2 chain. Instead, PEBP1 interacts primarily with the non-oxidizable sn-1 chain and the PE headgroup of diacyl-PE, providing additional stabilization. As we showed previously (PMID: 38230815), such stabilization is required for maintaining productive engagement of the sn-2 chain in diacyl-PE. Otherwise, the sn-1 chain of the diacyl-PE can enter the catalytic site and disturb or even replace the sn-2 chain (PMID: 38230815). In the case of alkenyl-PE, however, our new simulations indicate that PEBP1 does not interact with the sn-1 chain, but the sn-2 chain remains conformationally stable regardless of PEBP1 presence. We included Extended Data Figure 5 and Movies 5-6, which show the lack of specific interactions between PEBP1 and

alkenyl-PE, and Extended Data Figure 6, which demonstrate that the conformation of the substrate remains unchanged in the catalytic pocket with or without PEBP1 and also provides a structural rationale for the absence of interaction between alkenyl-PE and PEBP1. These results are described on pages 7-8 in the revised manuscript.

6. The authors used IL-13, UVB, and CCI as disease models to manipulate the expression of 15-LOX-1 and 15-LOX-2. However, these inducers may have additional off-target effects that influence the overall results (e.g., IL-13 facilitates ferroptosis via the IL-13-STAT6-SOCS1-SLC7A11 pathway in asthma).

The Reviewer is correct that UVB exposure has multiple effects on the skin, and, therefore, an off-target consequence which is unrelated to 15-LOX *is possible. However, this model is quite physiologically relevant, as 15-LOX is a critical regulator of skin homeostasis and inflammation (PMID: 23954555), and the upregulation of 15-LOX with UVB exposure has been documented (PMID: 36438817). Therefore, the UVB exposure model allows for a physiologically relevant upregulation of 15-LOX as a gain-of-function manipulation. Given the selectivity of 15-LOX for alkenyl-ETE-PE and diacyl-ETE-PE type substrates, potential off-target effects of this particular response to UVB are not expected to alter the conclusions from our experimental results.*

In asthmatic conditions, IL-13 induces 15-LOX-1 in acute lung injury (ALI) culture. Execution of ferroptosis depends on 15-LOX-1, although the enzyme is likely partially induced by STAT6 mediated pathways (there are several STAT6 binding sites in the promoter) (PMID: 10657944). In our previous publications (PMID: 29053969, PMID: 34762602), we demonstrated that 15-LOX-1 knockdown markedly lowers generation of hydroperoxy-phospholipids, in particular 15-HpETE-PE. We performed new experiments in which we exposed IL13-treated (10ng/ml for 5 days) control and 15LOX1 knockdown (KD) HAEC to RSL3 (10 nM for 24 hours). We found that 15-LOX-1 KD decreases RSL3-induced LDH release (cell death) in IL13-treated HAECs. We included this data in Figure 5l and described them on page 11 of the manuscript. We also discussed the models used in the studies on page 16 of the revised manuscript.

Previous studies by us (PMID: 30531185, PMID: 29053969) and others (PMID: 27838820, PMID: 30602353) have shown markedly increased 15-LOX-2 expression in brain tissue after traumatic brain injury (TBI). The increase in the pericontusional cortical areas occurs within the first 4 hrs after TBI vs the uninjured hemisphere as well as naïve controls and is primarily observed in neurons rather than glial cells. To examine the effect of 15-LOX-2 on neuronal death and PE oxidation after TBI we utilized a well-established in vitro TBI model of neuronal mechanical stretch injury that leads to ferroptotic death (PMID: 30531185). Injury to neuronal cells lead to decreased cell viability, which was rescued by Fer-1 as well as knockdown of 15-LOX. We next employed imaging mass spectrometry utilizing timsTOF fleX MALDI-2 (Bruker) to examine the effect of 15-LOX-2 on the generation of stretch-induced PE oxidation. We observed that ETE-PE species underwent marked oxidation upon injury, which was attenuated by Fer-1 as well as 15-LOX KD. To further investigate whether there were differences between alkenyl vs diacyl ETE-PE oxidation we performed additional experiments utilizing ion mobility and observed higher levels of alkenyl-HETE-PE vs diacyl-HETE-PE after in vitro TBI in a 15-LOX dependent manner. We included these new results in Figure 7c, f-j and Extended Figure 9d-f and described them on page 12 of the revised manuscript. New methods were also included in the Method section of the manuscript (see pages 23-25 and 28-29).

Genetically manipulating 15-LOX via KD, KO, or overexpression would be a more appropriate model for studying the distinct role of the 15-LOX-catalyzed alkenyl-ETE-PE peroxidation axis in ferroptosis.

In our study we used bone marrow PMN-MDSC obtained from mice with genetically manipulated 15-LOX (Alox12/15^{fl} Cre^{-} and Alox12/15^{fl} Cre^{+} mice). We found that low content of alkenyl-S-HpETE-PE was associated with low 15-LOX expression as evidenced by LC/MS and mRNA expression, respectively. The results obtained from PMNs from Alox12/15^{fl} Cre^{-} and Alox12/15^{fl} Cre^{+} mice were presented in Fig. 6d-f (Figure 8a-c in the revised manuscript). In response to the Reviewer's comment, we included a new dataset on the RSL3-induced death of PMN-MDSC obtained from 15-LOX null (Alox12/15^{fl} Cre^{-} and Alox12/15^{fl} Cre^{+}) mice. Briefly, PMN-MDSC were exposed to RSL3 (10µM, for 4hrs). After that cells were washed and incubated for another 16h. Cell death was estimated by Trypan Blue exclusion method. We found that PMN isolated from Alox12/15^{fl} Cre^{-} mice were more sensitive to cell death induced by RSL3 compared to Alox12/15^{fl} Cre^{+} mice. We described the protocols in the Methods section on page 26, presented these new results in Figure 8d and described them on page 13 of the revised manuscript.

Also, to what extent does phospholipid oxidation by LOX contribute to cell death induction?

The first stage of ferroptosis is a decision-making stage. In many cases, it is enzymatic and associated with activation (expression) of 15-LOX to generate hydroperoxy-PE species. Please see our response to comment 4.

To address the contribution of 15-LOX to cell death, we mimicked the 15-LOX-catalyzed stage of ferroptosis by employing a lipophilic azo-initiator of peroxyl radicals, AMVN, (2,2'-azobis(2,4-dimethylvaleronitrile)). The initiator spontaneously decomposes to yield a carbon centered radical which, in the presence of oxygen, converts into a peroxyl radical and hydroperoxy-phospholipids (HOO-PL). In the absence of transition metals, HOO-PLs do not undergo cleavage, however, they can still be reduced to the respective hydroxy-species. Therefore, we examined AMVN-driven oxidation and cell death in HT22 cells. We found that incubation of cells with AMVN resulted in significant generation of hydroperoxy-species of diacyl- and alkenyl- PE and PC but no cell death was detected under these conditions. When iron (as Ferric nitrilotriacetate) was added to the incubation media, cells started to die ferroptotically and a significant increase in the accumulation of lipid hydroperoxides and generation of oxidatively-truncated PE and PC species was detected. This was completely inhibited by ferrostatin-1, a ferroptotic cell death inhibitor (paper in preparation). This second stage of ferroptosis is linked to the production of oxidatively-truncated PE species and formation of protein adducts (PMID: 37245287). This stage is non-enzymatic and depends only on the presence of iron.

7. Fig2 h,i: Is cell death suppressed by knockout of 15LOX?

The data presented on Fig. 2h and Fig.2i (Figure 4a and 4b, respectively, in the revised manuscript) demonstrated that <u>exogenous</u> 15-LOX in the presence of either diacyl-S-ETE-PE or alkenyl-S-ETE-PE can induce ferroptotic cell death in A375 control (Fig. 2h: Figire 4a in the

revised manuscript)) or A375 GPX4 knockout cells (Fig. 2i; Figure 4b in the revised manuscript). The highest effect was detected in the presence of alkenyl-S-ETE-PE. Cell death was associated with the accumulation of diacyl-S-HpETE-PE and alkenyl-S-HpETE-PE species, respectively. Neither accumulation of PE oxidation products nor cell death were detected without exogenous 15-LOX-2. To more completely respond to this question, we also included new data demonstrating that PMN isolated from 15-LOX deficient mice were less sensitive to cell death induced by RSL3 (see our response to comment 6).

Is the oxidation of these lipids the cause of cell death?

Yes, oxidation of these lipids causes cell death. Previously we have demonstrated that incubation of cells with diacyl-S-HpETE-PE resulted in cell death which was inhibited by ferrostatin-1 (PMID: 27842066). We also included in the revised manuscript new results demonstrating that exogenous alkenyl-S-HpETE-PE effectively initiated ferroptosis in cells (see our response to comment 8). We also performed new experiments in 15-LOX-2-deficient neuronal cells as described above (see our responses to comments 6 and 10).

8. Fig4b: Increasing LOX expression with IL-13 alone does not facilitate ferroptosis, and ferroptosis is not enhanced without the addition of RSL3 and ETE. Does this mean that the oxidation of endogenous alkenyl-ETE-PE is insufficient to induce ferroptosis?

The Reviewer points to a very important issue on the role of different products of multistage phospholipid peroxidation in ferroptosis. We demonstrated that oxidation of endogenous alkenyl-ETE-PE to its primary product, hydroperoxy-alkenyl-ETE-PE, is insufficient to fully execute the ferroptotic program. In a separate series of experiments (please see also our responses to comments 4 and 6), we used an azo-initiator of peroxyl radicals (AMVN) which triggered marked accumulation of hydroperoxy-alkenyl-ETE-PE (alkenyl-S-HpETE-PE). This, however, did not cause increased sensitivity to ferroptosis. Next, we combined AMVN with Ferric nitrilotriacetate (Fe-NTA), which is capable of decomposing alkenyl-S-HpETE-PE to yield lipid radicals and subsequent electrophilic oxidatively-truncated phospholipid-species. The accumulation of the latter has been confirmed by LC-MS. The cleavage of HpETE-PE was required for the effective induction of ferroptotic death inhibitable by Fer-1. While this important question is not within the scope of the current work, we would like to mention that these secondary electrophilic peroxidation products are known to attack nucleophilic residues in proteins, including cell membrane proteins to form the adducts culminating in ferroptotic death (PMID: 37245287). Given that peroxidation of ETE-PE is characteristic of ferroptosis, it is noteworthy that ~20-50 proteins undergo this "oxidative PE-lipidation" process. Thus, hydroperoxy species of phospholipids, including alkenyl-S-HpETE-PE, are necessary but insufficient for the completion of the ferroptotic program.

9. Why does cell death occur when cytoplasmic lipids are oxidized, and what is the state and localization of phospholipids in the cytoplasm?

This is yet another fundamental question raised by the Reviewer. While it is not a direct goal of the current study, we would like to comment on it. Experimentally, ferroptosis has been discovered in 2012 (PMID: 22632970) and the initial works suggested that ER is the major

localization of the pro-ferroptotic events (PMID: 27842066). Later, numerous studies have been focused on the specific intracellular locales where the process commences. It has been demonstrated that essentially all organelles – endoplasmic reticulum (PMID: 27842066; PMID: 29512999) and Golgi apparatus (PMID: 30511023), mitochondria (Jung et al., 2021 PMID: 34102574; PMID: 37548939), lysosomes (PMID: 33340545), peroxisomes (PMID: 32939090), and nucleus (PMID: 34465893) - may be involved in the induction and execution of ferroptotic cell death pathway triggered by different stimuli (PMID: 34465893). These issues are being actively studied, and new intracellular compartments are being discovered as the initiating sites. Recent work identified mitochondria associated membranes (PMID: 40514428) and plasma membrane-associated ER (PMID: 40318637) as the intracellular compartments where the ferroptotic process begins. Notably, not only intracellular but extracellular initiation of ferroptosis has been documented (PMID: 38230815) which has led to the concept of non-cell autonomous nature of ferroptosis (PMID: 31341276). We discussed this matter in the revised manuscript on page 14.

Also, does cell death occur when cells are treated with oxidized alkenyl-ETE-PE?

In response to the Reviewer's question, we performed new experiments in which we investigated the effect of alkenyl-S-HpETE-PE on death of A375 GPX4 knockout (KO) cells. Alkenyl-S-HpETE-PE was biosynthesized and purified using recombinant 15-LOX-2. A375 GPX4 KO cells were incubated with either alkenyl-S-HpETE-PE (500 nM) or diacyl-S-HpETE-PE (500 nM) in the absence and in the presence of the ferroptosis inhibitor ferrostatin-1 for 24 hours. At the end of incubation cell death was evaluated using Sytotox green. We found that alkenyl-S-HpETE-PE was able to induce ferroptosis in A375 GPX4 KO cells. Quantitatively, the effect was similar to that observed in cells treated with diacyl-S-HpETE-PE. We included new results in Figure 4d and described them on page 9 of the revised manuscript. The method on synthesis of alkenyl-S-HpETE-PE was also added to the Methods section of the manuscript (see page 23),

10. Fig 6: A comparison with the group without CCI treatment is needed.

As requested, we added results on the contents of diacyl-S-ETE-PE and alkenyl-S-ETE-PE in brain of naïve mice. No differences in the contents of diacyl-S-ETE-PE and alkenyl-S-ETE-PE between contralateral areas of controlled cortical impact (CCI) exposed mice and naïve mice were detected. We have updated Figure 7ab with this data (Fig.6a in the previous version of the manuscript).

11. Is alkenyl-ETE-PE oxidation essential for causing CCI, or does it simply increase during disease progression?

We observed a marked increase in 15-LOX-2 expression after CCI and our new results obtained by using confocal microscopy indicate that this increase is primarily observed in neuronal cells. Knockdown of 15-LOX-2 in neuronal cells significantly attenuated cell death and alkenyl-ETE-PE oxidation. We included new results in Figure7c and 7f-j and described them on page 12 of the revised manuscript. Furthermore, we and others have shown that anti-ferroptotic agents improve neurocognitive outcome after traumatic brain injury (PMID:30531185; PMID:30264934).

12. Is alkenyl-ETE-PE oxidation similarly important for disease progression in an asthma model?

Our published data show that inhibition of 15-LOX-1 directly improves asthmatic airway epithelial cell (AEC) redox balance and lessens autophagic processes (PMID: 34762602, PMID: 32513718. PMID: 38987265). However, no human studies of specific inhibition of 15-LOX-1 linked ferroptotic processes have been performed. Interestingly, vitamin E (gamma-tocopherol), shown to inhibit 15-LOX and ferroptosis in vitro (PMID: 27842066), has been reported to lower eosinophilic inflammation and mucus production in asthmatic patients. (PMID: 28736267). Additionally, we previously established an association of airway epithelial cell expression of 15-LOX-1 with asthma severity (PMID: 19218191, PMID: 32513718). We discussed this on page 16 of the revised manuscript.

Reviewer #2 (Remarks to the Author): Comments:

• The authors suggest that there are two phases in ferroptosis: first phase mediated by LOX and second driven by non-enzymatic oxidation. What is the impact of each mechanism in generation of alkenyl-peroxides and diacyl-peroxides? Please discuss.

Ferroptosis begins with the selective peroxidation of PUFA-PE in which arachidonoyl (C20:4, ETE)- and adrenoyl- (docosatetraenoic, DTE, C22:4)-containing phosphatidylethanolamines (PE) species are converted into their hydroperoxy-PE-derivatives (PMID: 27842066). This process is not random but rather is highly specific as 15-HpETE-PE and 17-HpDTE-PE are the preponderant peroxidation products (PMID: 27842066). The selectivity and specificity point to the enzymatic nature of these reactions, and we demonstrated that two isoforms of 15-LOX – LOX-1 and LOX-2 – are effective generators of these products (PMID: 29053969). Alkenyl PE are preferred substrates of 15-LOX-catalyzed peroxidation vs diacyl-PE species. This is likely due to the enrichment of alkenyl phospholipids with ETE in the sn-2 position. Notably, oxidation of the vinyl bond at the sn-1 position of alkenyl phospholipids occurs at an incomparably lower rate (~500-fold more slowly than sn-2-ETE-PE). Importantly, we established that oxidation of the vinyl bond does not affect ferroptosis. Our new data with AGPS$^{-/-}$ cells (A375) clearly demonstrated that knockdown of this gene does not change their sensitivity to ferroptosis. In sharp contrast, incubation of cells with oleic acid (OA) or ETE leading to the marked enrichment of PE-plasmalogens with sn-2-OA or sn-2-ETE strongly decreases or increases the cells' sensitivity to ferroptosis, respectively. We included the data in the Results section (Figure 2hi and Figure 4e-g), described on pages 6 and 9 and discussed on pages 14-15 of the revised manuscript.

It has been reported that ACSL4 plays a significant role in the regulation of ferroptosis via biosynthesis of ETE-CoA (PMID: 37442770) and consequent synthesis of ETE-containing PE including diacyl-S-ETE-PE and alkenyl-S-ETE-PE. Therefore, the knockout of ACSL4 will affect the levels of these two PEs in cells. Indeed, our data demonstrated that the contents of both diacyl-S-ETE-PE and alkenyl-S-ETE-PE in ACSL4 knockout mouse fibroblasts were consequently lower (1.5- and 1.8-fold decreased compared to control cells, respectively). Moreover, while in wild type cells treated with RSL3 (500 nM), the levels of diacyl-S-HpETE-PE and alkenyl-S-HpETE-PE were increased 1.3- and 1.9-fold, respectively, no changes in their levels were detected in ACSL4 knockout cells. Thus, affecting the biosynthetic pathways of PE

species can impact the first stage of ferroptosis. We included the data in the Results section (Figure 4kl) and described them on pages 9-10, and 14-15 of the revised manuscript.

Hydroperoxy-PE (HOO-PE) is unstable and can be readily decomposed in the presence of transition metals, including $Fe^{2+/3+}$, to radicals O•-PE or OO•-PE. This designates the commencement of the second stage of pro-ferroptotic peroxidation representing a non-enzymatic peroxidation process (PMID: 30217775), which includes beta-scission leading to the oxidative truncation of the fatty acid residues in HOO-PE. As a result, a large variety of different strongly electrophilic products are formed which can readily react with nucleophilic amino acids in proteins thus generating adducts. This "oxidative protein lipidation" is believed to disrupt the lipid bilayer and the entirety of biomembranes, including the cell membrane, thus causing death (PMID: 37245287). Our previous work has documented the formation of PE-oxidatively lipidated proteins in cells undergoing ferroptosis. High levels of oxidizable ETE-PE alkenyl species and their higher rate of peroxidation by 15-LOX makes them influential regulators of the pro-ferroptotic outcome. Alternatively, HOO-PE can be reduced (by GSH/GpX4) to stable alcohols. This reductive route is a dead-end of ferroptosis. We discussed this on page 15 of the revised manuscript.

• The experiments with purified 15LOX were performed using liposomes with equal proportions of DOPC to diacyl-PE or DOPC to alkenyl-PE (50 : 50). Diacyl- and alkenyl-PEs have different properties in the bilayer, which potentially can influence the structure of liposomes thus leading to artificial differences in enzyme activity. Is it possible to repeat the experiment using a mixture of diacyl- and alkenyl-PEs substrate in the same liposome? Such an experiment is closer to the situation in cell membrane and can better characterize substrate preferences of purified 15LOX-1/2.

As requested by the Reviewer, we performed new experiments in which we prepared liposomes using natural lipids obtained from HT22 cells. The liposomes were exposed to 15-LOX-2 for 2.5 and 5 minutes at 37°C. At the end of incubation, lipids were extracted, and diacyl-S-ETE-PE and alkenyl-S-ETE-PE were analyzed by LC/MS. We found that 15-LOX-2 induced oxidation of alkenyl-SA-ETE-PE almost two times more effectively than diacyl-SA-ETE-PE. These new results are included in the revised manuscript (Figure 2f, page 5).

• Please explain in the discussion more clearly why PEBP1 did not stimulate peroxidation of alkenyl-PE.

PEBP1, through interactions involving the sn-1 ester linkage of diacyl-ETE-PEs and residues of 15-LOX, stabilizes the sn-1 chain of these lipids within the hydrophobic cavity, thereby preventing it from entering the catalytic site and disturbing sn-2 peroxidation. In alkenyl-ETE-PEs, such stabilization is not required: lacking the ester linkage, the sn-1 chain does not bind PEBP1 but instead associates with the 15-LOX surface, and even when it occasionally enters the catalytic site, the conformational constraints imposed by the vinyl ether linkage at sn-1 prevent perturbation of sn-2, allowing peroxidation to proceed. We discussed this on page 16 in the Discussion section of the revised manuscript.

• The data on asthma patients were obtained in extremely small groups (3 to 5). The conclusions could be strengthened by analyzing additional samples.

In response to this comment, we performed additional measurements of PE peroxidation products in new samples. We obtained 2 control samples as well as 3 and 5 samples from patients with mild and severe asthma, respectively. The new results on 15-LOX-1 expression and content of diacyl-S-HpETE-PE and alkenyl-S-HpETE-PE species were included in the updated Figures 8f and 8g, respectively. These new measurements confirmed and enhanced our conclusions on the link between 15-LOX-1 expression and elevated levels of peroxidized plasmalogens in asthma. We described these results on page 13 in the Results section of the revised manuscript.

Reviewer #3 (Remarks to the Author):

Author reported alkyl/alkenyl-ETE-PE as potential substrates of enzymatically catalyzed peroxidation. By using redox lipidomics, biochemical, biophysical and genetic approaches combined with molecular dynamics simulations, we established that both isoforms of 15LOX (15LOX-1 and 15LOX-2) selectively oxidize alkyl/alkenyl-ETE-PE (but not 43 alkyl/alkenyl-ETE-PC) species to 15-HpETE-PEs and trigger ferroptotic cell death in vitro and in vivo more effectively than via oxidation of diacyl-ETE-PE. Thus, 15LOX-catalyzed bias towards oxidation of alkenyl-ETE-PE may represent a new therapeutic target. In my opinion this Manuscript should be consider following points.

We are thankful to the Reviewer for the concise analysis of several of our results and for the proposed changes in the presentation of our concepts and experimental data.

1. The abstract lacks precision and depth. It should clearly define the problem, methodology, key findings, and conclusion in a more structured format for better reader comprehension.

As recommended, we restructured the Abstract according to the Reviewer's instructions (see below).

Ether (alkyl/alkenyl) phospholipids, particularly phosphatidylethanolamine (PE) and phosphatidylcholine (PC), are broadly represented in membranes, but their physiological functions are poorly characterized. The antioxidant role of plasmalogens realized via sacrificial oxidation of the sn-1 vinyl bond has been hypothetically associated with an anti-ferroptotic regulatory function. Alternatively, peroxidation of polyunsaturated fatty acid (PUFA) in the sn-2-position of alkenyl-PEs can be pro-ferroptotic. Since 15-LOXs generate 15-HpETE-PEs as signals initiating the execution of ferroptotic death, we explored alkyl/alkenyl-ETE-PE as potential substrates of enzymatically catalyzed peroxidation. Using redox lipidomics, biochemical, biophysical and genetic approaches combined with molecular dynamics simulations, we established that both isoforms of 15-LOX (15-LOX-1 and 15-LOX-2) selectively oxidize alkyl/alkenyl-ETE-PE (but not alkyl/alkenyl-ETE-PC), forming 15-HpETE-PEs, triggering ferroptotic death, independently of the presence or absence of the vinyl bond. We showed that the LOX-catalyzed peroxidation rate of the sn-1 vinyl bond is ~500-fold lower than ~~of~~ sn-2-ETE-PE, thus excluding the antioxidant role of plasmalogens in ferroptosis. Using animal and human tissues and cells we established that 15-LOX-driven production of sn-1-alkenyl-sn-2-15-HpETE-

PE may act as a pathogenic factor in several acute and chronic diseases, including asthma, cancer, brain trauma and skin UVB-injury. Thus, 15-LOX-catalyzed bias towards oxidation of alkenyl-ETE-PE may represent a new therapeutic target.

2. The literature review section is insufficient and fails to provide a comprehensive background. It should include more recent and relevant studies to establish a strong foundation for the research problem.

In response to this important comment, we have enhanced the following background and added it to pages 13-14 of the revised manuscript:

Several recent genetic studies indicate that alkenyl-phospholipids are pro-ferroptotic (PMID: 33731874; PMID: 34167916, PMID: 37979715, PMID: 32939090), likely due to enzymatic enrichment of alkenyl-PE with oxidizable ETE at the sn-2 position (PMID: 36782012). In contrast, another group of reports has shown peroxisomal and ether-phospholipid deficiencies increase cellular vulnerability to ferroptosis (PMID: 39367496, PMID: 36178986). Finally, other publications state that alkenyl-phospholipids do not affect ferroptosis at all (PMID: 35809566). It is possible that these apparently inconsistent results reflect the importance of peroxidation of the sn-2 acyl chain (the oxidizability of which varies across cell lines) – rather than of the vinyl ether linkage – in ferroptosis. The vinyl ether bond of plasmalogens is oxidizable, however, (PMID: 9169616) and thereby prevents lipid oxidation in some contexts (PMID: 7980601, PMID: 9101253, PMID: 9895222), necessitating structural characterization of alkenyl/acyl-PE peroxidation products during ferroptosis.

3. The methodology is not well-justified. It is crucial to explain why the selected methods were chosen over other alternatives and how they are suitable for addressing the research question.

To address the major research goals of this project, we employed a comprehensive series of different methodological approaches and protocols adequately characterizing the multistage nature of the ferroptotic process with a particular focus on the enzymatic initiation of peroxidation by 15-LOX and subsequent propagation and execution of ferroptosis via formation of radical intermediates, and generation of oxidatively truncated electrophilic products and their adducts with proteins. We have employed genetic and pharmacological approaches to manipulate the expression and activity of 15-LOX and explore its role in the generation of primary hydroperoxy/hydroxy- peroxidation products in alkenyl-acyl- vs diacyl-lipids. We have pioneered the field of redox lipidomics (PMID: 33679610, PMID: 31071242, PMID: 30928338, PMID: 20950335, PMID: 16797512) and the use of different protocols of liquid chromatography-mass spectrometry (LC-MS). This permitted us to directly monitor the peroxidation process in its entity and the important individual peroxidized species. Notably, our redox-lipidomics techniques provide direct information on the oxidative modification of PUFA phospholipid substrates and formation of specific products. This is in sharp contrast to commonly used indirect techniques with fluorogenic model substrates which are inadequate for studies of ferroptosis – as has been emphasized in several recently published guidelines for assessments of ferroptotic analytic methodology (PMID: 35760871, PMID: 38908072). In addition, we applied computational modeling with extensive molecular dynamics simulations. Finally, we employed a variety of biochemical, biophysical, and cell biology models and performed several experiments to assess the significance of our findings in vivo and their potential relevance to aberrant metabolism in

disease conditions such as asthma, cancer, acute brain and skin injury. In combination all these modern techniques allowed us to discover that alkenyl/acyl-PE/diacyl-PE ratios define cellular susceptibility to ferroptosis mediated by preferential oxidation of the alkenyl species.

4. Figures and tables are not well-organized, and some are missing proper legends and labels. Ensure all data representations are clear and accompanied by sufficient explanations.

To improve the organization of the figures, we thoroughly went through all previous and new main and supplementary materials of the revised manuscript. Our goal was to be sure that all presented data have sufficiently detailed descriptions and explanations and correspond with the textual content of the manuscript. Please note that no results in the manuscript have been presented in tables.

5. The statistical analysis is weak or incomplete. Provide detailed information about the statistical tests used, including the rationale behind their selection and their relevance to the data.

We updated the statistical analysis section in the Methods section of the revised manuscript. Please see page 29 in the revised manuscript.

6. The results are not discussed in the context of existing literature. Compare the findings with similar studies to highlight the significance and novelty of the work.

Several paragraphs have been added to the Discussion section (along with new references) to more comprehensively compare our results with previously published data and more clearly document the significance and novelty of the current work

7. There are several grammatical and typographical errors. A thorough proofreading is needed to improve the overall readability and professionalism of the manuscript.

The text has been thoroughly proofread, and grammatical and typographical errors have been fixed.

8. The conclusion is too vague. Summarize the key findings more effectively and provide a clear statement on how the study advances knowledge in the field.

In response to this comment, we have updated the conclusion on page 17 of the revised manuscript:

In conclusion, the multistage process of ferroptotic lipid peroxidation is strongly regulated by the availability of polyunsaturated substrates and specific classes of phospholipids, particularly PUFA-PEs. Among them, PUFA-PE plasmalogens are preferred substrates due to more effective enzymatic peroxidation of their sn-2 chain by 15-LOX (PMID: 27842066, PMID: 38230815, PMID: 38309267).

While the sn-1 vinyl bond of plasmalogens can be oxidized by 15-LOX, the efficiency of this reaction is more than two orders of magnitude lower than peroxidation of sn-2-ETE-PE. Thus, the so-called "antioxidant" function of plasmalogens is not realized as a mechanism of ferroptosis regulation. Pro-ferroptotic 15-LOX-catalyzed generation of hydroperoxy-PUFA-PE from the plasmalogen sn-2-PUFA precursors may be an alternative pathway for the biosynthesis of pro- and anti-inflammatory lipid mediators (PMID: 27842066). A specific feature of this pathway includes initial peroxidation of PUFA-residues esterified into PUFA-PE plasmalogens. Subsequent hydrolysis of thus-formed oxygenated products may lead to the release of free oxygenated PUFA as lipid mediators.

9. Some references are outdated, and a few citations are missing. Ensure that all references are current and properly formatted according to the journal's guidelines.

As recommended, we updated the list of references in the manuscript and included several new important ones directly relevant to the role of plasmalogens in regulation of ferroptosis. All references have been formatted according to the Nature Communications guidelines.

10. The manuscript does not adequately address future research directions. Suggest specific areas where the study's findings could be further explored to strengthen the manuscript's impact. ** See Nature Portfolio's author and referees' website at www.nature.com/authors for information

We have added the following piece of text with future directions to the Discussion section (page 17):

Our work has demonstrated that PE-plasmalogens play an important role in regulation of ferroptosis. While sn-2-PUFA-PE plasmalogens sensitize cells to ferroptosis, non-oxidizable sn-2 saturated or mono-unsaturated PE-plasmalogens are negative regulators of the ferroptotic process. Because these propensities of plasmalogens are linked to their metabolism by 15-LOX, it will be important to determine whether other LOXs, e.g. 5-LOX and 12-LOX, display selectivity towards plasmalogen PUFA-PE species but not plasmalogen-PCs. Given that ferroptosis has been associated with pathogenesis of several major diseases, the opportunity of targeting ferroptotic death aimed at the production of oxidizable vs non-oxidizable plasmalogenic substrates may be a highly promising direction of future research in several chronic diseases (e.g., cancer, neurodegeneration) and acute injuries (e.g., brain trauma, radiation syndrome).

Once again, we thank the Reviewers for the very useful and constructive comments and overall positive response to our manuscript. We believe we have fully addressed all the comments and the manuscript is now acceptable for publication in Nature Communications

Kind regards,

Hülya Bayir
Valerian Kagan

Reviewer #1 (Remarks to the Author):

The authors have performed additional experiments to address several certain gaps and questions from reviewers. However, further questions remain. The reviewer's comments/questions are as follows:

1. The authors additionally show the level of diacyl- and alkenyl-PE in the plasma membrane of HBE cells, while HAECs were used for the determination of diacyl- and alkenyl-PE and PC in mitochondria and the cytosol. However, Fig. 1b and 1c showed different lipid profiles for these cells.

HAECs were obtained from human subjects, hence they were available in limited amounts. To minimize the required numbers of cells, we employed the protocol with fluorescamine-labeling of PE species in plasma membrane. This approach has been widely used for detection and quantitation of PE in plasma membrane of living cells (PMID: 87001). We utilized fluorescamine-labeled cells with inhibited aminophospholipid translocase. At the end of incubation, lipids were extracted and resolved by LC/MS. We found that the contents of alkenyl-ETE-PE species were significantly higher compared to their respective diacyl-ETE-PEs. Among all ETE-PEs, alkenyl-ETE-PE species – P-ETE-PE, PO-ETE-PE, S-ETE-PE and O-ETE-PE - were predominant and localized in plasma membrane of HAEC. We presented these new data in Figure 1f, Extended Data Figure 1e and described the results on page 5 of the revised manuscript.

In addition, we isolated mitochondria, ER and plasma membrane from HBE cells by density gradient centrifugation and performed analysis of diacyl-ETE and alkenyl-ETE-PE and -PC species. We found that distribution pattern for diacyl-ETE-PE and alkenyl-ETE-PE species was similar for plasma membrane, ER and mitochondria. Among those, alkenyl-P-ETE-PE and diacyl-S-ETE-PE were predominant species in isolated fractions. No significant differences in the contents of diacyl-P-ETE- and alkenyl-P-ETE-PE as well as diacyl-O-ETE- and alkenyl-O-ETE-PE were detected. Analysis of PC species revealed that diacyl-ETE-PC were the major ETE-PC species in plasma membrane, ER and mitochondria. We added the description of the method for isolation of organelles by differential centrifugation in the Methods section (page 21), presented new data in Extended Data Figure 2 and described the results on page 5 of the revised manuscript.

2. The enrichment of alkenyl-PE (especially alkenyl-ETE-PE) in several cell lines and tissues mainly resulted from TMEM164 as the authors mentioned? Are there any critical factors that influence this event?

The enzymes of de novo plasmalogen biosynthesis (e.g. AGPS, TMEM189) are not known to have selectivity towards ETE or other PUFA. To our knowledge, TMEM164 is the first plasmalogen biosynthetic enzyme which has been shown to have such selectivity (PMID: 36782012), suggesting that the sn-2 PUFA enrichment of alkenyl-PE is a result of acyl chain remodeling. While CoA-independent trans-acylase activity was first reported in 1983 (PMID: 6417134), TMEM164 was identified as the gene encoding this enzyme only two years ago (PMID: 36782012). As a result, there are few studies on TMEM164 regulation. Nonetheless, early reports suggest that inflammatory cytokines may increase CoA-independent trans-acylase activity (PMID: 7947995), while others found that substrate availability may regulate the activity of this enzyme (PMID: 11204449), implying that it is constitutively active. Similarly, sufficient

levels of ETE donors (e.g. diacyl-ETE-PC) are required for TMEM164 to synthesize alkenyl-ETE-PE. We discussed this in the revised manuscript on page 16.

The new data demonstrated that AGPS and TMEM189 (catalytic enzymes in ether lipid biosynthesis) did not affect ferroptosis sensitivity. Why is that? The authors state that "The depletion of TMEM189 causes proportional increases in alkyl species analogous to the alkenyl species which are depleted, maintaining levels of PUFA-containing alkyl/alkenyl-PE" (page 9). Does the observation that ferroptosis sensitivity is unchanged in AGPS and TMEM189-KO cells indicate that, under RSL3 treatment, either alkyl-PE or alkenyl-PE is sufficient? If so, does this suggest that the importance of alkenyl-PE is limited to conditions under LOX induction?

The effects of AGPS and TMEM189 knockdown suggest that while alkenyl-ETE-PE are preferred 15-LOX substrates, in live cells the amount of sn-2-ETE-PE – whether alkyl, alkenyl, or diacyl – is the primary determinant of sensitivity to GPX4-inhibition-induced ferroptosis. Compensation between different types of sn-2-ETE-PE is possible because known peroxidation substrates (e.g. diacyl-ETE-PE [PMID: 27842066, 27842070]) are massively in excess for ferroptosis execution. Further supporting the notion that sn-2 PUFA enrichment of PE plasmalogens is the key determinant of the contribution of plasmalogens towards ferroptosis sensitivity, many enzymes promoting ETE esterification are highly influential in ferroptosis – including ACSL4, LPCAT3, and FATP2. We discussed this in the revised manuscript on page 16.

Of note, in the current manuscript we presented results demonstrating that 15-LOX-2 induced oxidation of alkenyl-S-ETE PE is almost two times more effective than diacyl-S-ETE-PE in liposomes. This preferential peroxidation of alkenyl-S-ETE PE was not observed when peroxidation of phospholipids was initiated non-enzymatically by a lipophilic initiator of peroxyl radicals, 2,2'-azobis (2,4-dimethylvaleronitrile) (AMVN).

If so, does this suggest that the importance of alkenyl-PE is limited to conditions under LOX induction?

We thank the Reviewer for this insightful comment. We agree that the "bottle neck" will be different for enzymatic (LOX) and non-enzymatic (HO•, AMVN•) driven peroxidation. Our presented effects with plasmalogens are most relevant to LOX-dependent processes – which we have demonstrated by a number of genetically and physiologically induced factors (IL-13, TBI, etc.) – though other groups have also reported a role for ether lipids in RSL3-induced ferroptosis (PMID: 32939090, 33731874).

To experimentally test the contribution of TMEM164-mediated PUFA-PE plasmalogen synthesis towards ferroptosis sensitivity, we used siRNA to knock down (KD) TMEM164 in A375 melanoma cells. We found that in contrast to AGPS KD, TMEM164 KD decreased sensitivity to RSL3-induced ferroptosis. Given the relatively short timeframe of these experiments (<96 hours), siRNA knockdown cannot be expected to completely deplete the phospholipid products of these enzymes. Thus, TMEM164 KD may deplete PUFA-PE plasmalogens more effectively than AGPS KD due to remodeling being the predominant pathway of PUFA-PE plasmalogen biosynthesis. Indeed, lipidomics analysis confirmed that TMEM164 KD increased the ratio of diacyl-ETE-PC to alkenyl-ETE-PE. We added the description of the method in the Methods section (page 25) and presented the new results in Figure 4k-m and described them in the revised manuscript on page 10.

3. Although the site and localization of ferroptosis is not the goal of this current study, it is still unclear why the author measured alkenyl-PE in the mitochondria as a representative organelle.

In our study we were using HAEC in which we induced expression of 15-LOX-1 by IL13 and detected oxidized alkenyl-ETE-PE species, including alkenyl-HpETE-PE. A significant accumulation of alkenyl-S-HpETE-PE was detected in the cytosolic fraction versus the mitochondrial fraction upon treatment with IL13. The absence of significant changes in the levels of mitochondrial alkenyl-S-HpETE-PE is concordant with the low expression of 15-LOX-1 detected in mitochondria of IL13-treated cells. Our results demonstrate that while the content of alkenyl-ETE-PE in mitochondria is comparable with that obtained in cytosol, their oxidation in HAEC was fully dependent on the IL13-induced expression of 15-LOX-1.

How does mitochondria relate to ether-PL?

Mitochondria are one of the major sites for iron utilization and production of reactive oxygen species, fueling the lipid peroxidation process that may in some cells define ferroptosis (PMID: 38366038). Ether lipids, including alkenyl-PE species, are crucial for mitochondrial membrane structure and redox balance (PMID: 39303982) and comprise up to 40% of the total mitochondrial PE pool (PMID: 38129691). In addition to de novo synthesis, PE can also be formed in the inner mitochondrial membrane through decarboxylation of PS (PMID: 27650064). However, PS decarboxylase primarily uses PS as its substrate to produce diacyl-PE, not alkenyl-PE (PMID: 23124206). It has been demonstrated that ferroptosis is associated with dramatic morphological changes of mitochondria including membrane rupture (PMID: 31810634) and generation of pro-ferroptotic oxygenated PE species in mitochondria (PMID: 34102574). Some cells are extremely sensitive to ferroptotic cell death. We have reported that in H9c2 cardioblasts, RSL3 – a typical inducer of ferroptosis – resulted in generation of oxygenated PE and, damage mitochondria within 1hr; it caused cell injury and ferroptotic death very rapidly, in 3-4 hrs vs tens hrs in other types of cells (PMID: 34102574; PMID: 37245287, PMID: 36870109). Moreover, 15-LOX levels and activity are significantly upregulated in mouse cardiomyocyte mitochondria during stress conditions like ischemia-reperfusion, heart failure, and chemotherapy-induced cardiotoxicity. Furthermore, increased 15-LOX expression/activity drives mitochondrial dysfunction, promotes ferroptosis, and causes mitochondrial damage (PMID: 36987924). Taking into account that the content of alkenyl-PE in mitochondria is high, and their oxidation is dependent on the level of 15-LOX, we wanted to attract the attention of the research community to this interesting aspect of mitochondria-initiated ferroptosis. Of course, more work will be needed for detailed characterization of this ferroptotic pathway. We discussed this in the revised manuscript on page 17.

If the authors wanted to demonstrate their levels in various organelles, there are several major sites for ferroptosis which more related to alkenyl-PE (such as ER or peroxisomes).

The ferroptotic process can be initiated in different organelles dependently on the sufficiency of oxidizable substrates, catalysts, and dysregulation of controlling mechanisms, including thiols, iNOS/NO•, and oxidoreductases coupled with endogenous antioxidants (e.g., CoQ10-H2). For example, disorganization of mitochondria is likely to be involved in pro-ferroptotic activity.
A recent study demonstrated the important involvement of mitochondria-associated membranes (MAMs) - connecting ER- and mitochondria- in ferroptosis. Details of ER ferroptotic reactions and the role of ER-based initiation sites and the spreading of the ferroptotic "wave" are currently under thorough scrutiny. The specificity and effectiveness of different hydroperoxy-

phospholipids as inducers of ferroptosis is also not sufficiently studied although a variety of individual molecular species of hydroperoxy-PE seem to be universally effective (PMID: 38230815). Lately, hydroperoxy-derivatives of other phospholipids, like hydroperoxy-PA, have been also found to exert pro-ferroptotic activity (PMID: 38309267). We agree with the Reviewer that exploring the ether lipids of peroxisomes and ER during ferroptosis would be an interesting topic of future studies. Although plasmalogen biosynthesis begins in peroxisomes and these organelles are a major producer of reactive oxygen species (PMID: 22178243), unbiased explorations have not implicated peroxisomes as a common initiation site of pro-ferroptotic lipid peroxidation (PMID: 36747055). We discussed this in the revised manuscript on page 17.

4. The author used the PE externalization and labeling method to determine diacyl- and alkenyl-PE in the plasma membrane. To measure total lipid contents in the plasma membrane, is this protocol appropriate for the current study compared to other protocols (e.g., PMID: 22555243)? So that the levels of PC can be also measured.

We are thankful to the Reviewer for pointing to a relevant paper (PMID: 22555243). This paper, however, describes a protocol for the generation of giant plasma membrane vesicles primarily useful for the study of proteins, not lipids. The method includes several steps including blebbing and consequent vesiculation in the presence of paraformaldehyde (PFA)/dithiothreitol (DTT) and N-ethylmaleimide. These treatments/reagents may interfere with the process of ferroptosis execution. For example, as a strong reductant, DTT causes reduction of hydroperoxy-phospholipids into respective hydroxy-phospholipids. In addition, as a strong nucleophile, DTT prevents the formation of adducts of proteins with oxidatively truncated strongly electrophilic PE oxidation products (PMID: 37245287). In addition, PFA/DTT causes PE depletion in giant vesicles by promoting its covalent cross-linking to nearby membrane proteins, especially when used with PFA which cross-links proteins, creating stable complexes that effectively remove PE from the lipid extractable pool (PMID: 21709267). Because our goal in this study was to analyze and quantitatively assess diacyl- and alkenyl-PE molecular species in plasma membrane, we chose to avoid the use of this method. Instead, we isolated plasma membrane from HBE cells by differential gradient centrifugation and performed analysis of ETE-containing diacyl- and alkenyl-PE and PC species. Please see our response to comment #1 for a more detailed description of this experiment.

5. The authors performed additional HT-22 stretching experiments. However, the Reviewer recommended that the role of alkenyl-HpETE-PE in ferroptosis upon Fer-1 treatment and 15-LOX-2 KD in TBI should be performed in CCI mice model (same as the utilization of genetically manipulated 15-LOX in PMN-MDSC).

We thank the Reviewer for this recommendation. Indeed, the very low level of 15-LOX in healthy brain (particularly in neurons) (PMID: 30531185), increases many-fold after acute brain injury (CCI) (PMID: 30531185, PMID: 40947949). Thus, targeting pro-ferroptotic 15LOX activity may be protective against CCI induced injury. Notably, our previous in vitro work has identified Fer-1 as an effective 15-LOX inhibitor (PMID: 33126055). However, Fer-1 demonstrated a relatively poor efficiency in penetrating blood-brain barrier (BBB), negatively affecting its therapeutic potential towards neuronal 15-LOX (PMID: 39984310; PMID: 39913870). With this in mind, we chose to employ an alternative inhibitor, a polyphenolic flavonoid compound, baicalein (5,6,7-trihydroxyflavone), known to effectively suppress 15-LOX activity (PMID: 16500106), and act as a strong anti-ferroptotic radical scavenger (PMID: 27037021, PMID:

8215413, PMID: 30531185). Importantly, CCI resulted in accumulation of oxidized diacyl-ETE-PE and alkenyl-ETE-PE, increased expression of 15-LOX and acyl-CoA synthetase long-chain family member 4 (ACSL4, the enzyme that generates substrate for the esterification of arachidonic/adrenic acid into PE), and depletion of glutathione in the ipsilateral cortex (PMID: 30531185). These effects were fully prevented by post-injury administration of baicalein indicating that the 15-LOX pathway might be a valuable therapeutic target after traumatic brain injury. In addition, post-injury administration of baicalein attenuated oxidation of arachidonic/adrenic acid-containing-PEs and improved spatial memory acquisition versus vehicle. In behavioral experiments, we also found that baicalein treatment improved spatial memory acquisition (compared with vehicle controls) (PMID: 30531185). We presented these new data in the Figure 7j, described them in the text and discussed it in revised manuscript on pages 13 and 18, respectively.

6. More data (e.g., IL-13) linking 15-LOX expression, alkenyl-PE, and ferroptosis in asthmatic patients could improve clinical relevance and translational insight for this study.

FeNO (fractional exhaled nitric oxide) is one of the most common biomarkers for Type-2 airway inflammation in asthma (PMID: 24337046). Therefore, to enhance "clinical relevance and translational insight of the study," we performed comparative analysis of the levels of alkenyl-S-HpETE-PE with FeNO in a group of patients. We established that there is a strong positive correlation between alkenyl-S-HpETE-PE levels and FeNO• (r=0.62 p<0.002). Given the previously published relationship of FeNO to 15-LOX-1(PMID: 29053969) in human asthma, this is not surprising. Of relevance, however, 7 of the severe asthma patients were on T2-targeted monoclonal antibody therapy, with the differences by asthma severity or FeNO not impacted by their use. These comparisons suggest the alkenyl-S-HpETE-PE levels are identifying or contributing to a T2-associated factor which drives asthma severity and appears to be poorly responsive to T2-targeted monoclonal antibody therapy. We presented these new results in Extended Data Figure 10h and described and discussed them on pages 14 and 19 of the revised manuscript, respectively.

Other comments

Several typographical errors and labeling inconsistencies remain throughout the revised manuscript. Examples include missing labels for Fig. 8g and Extended Data Figs. 9f–9g, and the incorrect notation "stretch + Fer-1" in Fig. 7f. The manuscript would benefit from thorough proofreading prior to resubmission.

We are thankful to the Reviewer for pointing to several graphical typos and errors. The MS has been thoroughly proofread and necessary corrections made.

No additional comments from Reviewer 2.

Reviewer #3 (Remarks to the Author):

The revised version of the manuscript includes all remarks and modifications indicated. The

main concerns of the manuscript have been solved. In my opinion, the provided version is now suitable for publication.

We appreciate the reviewer's positive comments, and we are pleased that our previous revision has addressed the comments adequately.

Once again, we thank the Reviewers for the very useful and constructive comments and overall positive response to our manuscript. We believe we have fully addressed all comments, and the manuscript is now acceptable for publication in Nature Communications

Kind regards,

Hülya Bayir
Valerian Kagan
